# Supplementary material for: Using mobile sequencers in an academic classroom
Source: eLife. 2016 Apr 7;5:e14258. doi: 10.7554/eLife.14258 (PMC4869913; doi:10.7554/eLife.14258)
Supplement: Supplementary file 1. — DOI: http://dx.doi.org/10.7554/eLife.14258.005 [file elife-14258-supp1.docx]

**Supplemental Note 1**

**Class website information**

**Course objectives:**

At the end of the course the student should be able to:

**Theoretical:**

- Describe the different types of DNA sequencing technologies.
- Understand the broad scale of applications and algorithms for DNA sequencing.
- Describe Nanopore-sequencing technology in detail.
- Contrast the ethical concerns and opportunities the current DNA sequencing revolution faces.

**Hands-on:**

- Operate the handheld sequencer (the MinION) and generate data themselves.
- Understand real-world challenges in DNA sequencing generation and wrangling.
- Gain experience in finding and developing bioinformatics tools to interpret and analyze sequencing data.
- Critically think about the analysis of DNA sequencing data.

## 2015 Syllabus

Week Date Topic

1 11-Sep Intro to DNA and DNA sequencing technologies

2 18-Sep Applications of DNA sequencing: Human genetics

3 25-Sep Applications of DNA sequencing technologies: Metagenomics

4 2-Oct Application of DNA sequencing: Forensics. Ethics of genomics

5 9-Oct Mobile health

6 16-Oct Mobile sequencing

7 23-Oct Hackathon I: “From Snack to Sequence”

8 30-Oct Analysis pipelines(1)

9 6-Nov Presentations for hackathon I

10 13-Nov Hackathon II: “CSI Columbia”

11 20-Nov Analysis pipelines (2)

12 27-Nov [No class]

13 4-Dec Presentations for hackathon II

14 11-Dec DNA as storage devices or computers

**Assignments and grading**

***Reading assignments***

You are expected to read the paper and understand the main concepts and terms before the class.

***Presentations***

The class has a few lessons that include team presentations. The length of each presentation is 10min and will be delivered by one member of the team. To encourage fairness and participation, the presenter will be randomly selected at the beginning of the presentation.

***Coding/Written assignments***

Teams are expected to code their own assignments. It is OK to brainstorm high-level ideas with other teams. It is OK to consult online forums. However, the submitted code should be fully written by members of the team. No exceptions. To maximize impact, all code should be submitted under the GNUv2 license.

**Grades**

• Participation in class discussions: 25%

• Hackathon1: 25% (10% presentation + 15% code submission)

• Hackathon2: 25% (10% presentation + 15% code submission)

• Final project: 25% (**see supplemental note 5**)

Copyright:

© 2016 Zaaijer et al. This teaching material is provided under the Creative Commons Attribution-Share Alike 4.0 International License
