# Supplementary material for: Using mobile sequencers in an academic classroom
Source: eLife. 2016 Apr 7;5:e14258. doi: 10.7554/eLife.14258 (PMC4869913; doi:10.7554/eLife.14258)
Supplement: Supplementary file 2. — DOI: http://dx.doi.org/10.7554/eLife.14258.006 [file elife-14258-supp2.docx]

**Supplemental Note 2**

Protocol for setting up a Hackathon using the MinION:

***Sample preparation for MinIONs***

For “from snack to sequence”: Prior to the hackathon, food ingredients for which the genome is included in the NCBI database were selected from the supermarket. The DNA of seven food products was isolated using standard procedures, including four raw products (tomato, kale, beef, and bacon) and three that were cooked (salmon, shrimp, and mussel). The DNA samples were fragmented using Covaris g-TUBEs and further processed by end-repair and dA tailing using the NEBNext Ultra II End Repair/dA-tailing Module. The sequencing libraries were prepared according to version 6 of Oxford Nanopore Technologies Protocol (MAP006). DNA concentration was measured by Qubit Fluorometric Quantitation (Thermo Fisher). The composition of the DNA library samples prepared for the 5 groups is indicated below:

| **Students** | **Ingredient** | **ratio** |
| --- | --- | --- |
| group1 | beef | 80% |
|  | tomato | 20% |
| group2 | bacon | 80% |
|  | salmon | 20% |
| group3 | kale | 10% |
|  | mussel | 60% |
|  | shrimp | 30% |
| group4 | beef | 20% |
|  | tomato | 80% |
| group5 | bacon | 20% |
|  | salmon | 80% |

For “CSI Columbia”, DNA samples for Craig Venter (NS12911) and the HapMap consortium (NA12890) were purchased from the Coriell Institute. Genomic DNA information for Yaniv Erlich is available online at <https://dna.land/consent>. Genomic DNA from Yaniv Erlich was extracted from saliva using the Qiagen QIAamp® DNA Blood Mini Kit. DNA samples were enriched for fragment size by gel electrophoresis and isolation of fragments >3kb (Qiagen® Gel extraction kit). These fragments were sheared using Covaris g-TUBE size ~6 kb. The generation of the DNA libraries was done as described for “from snack to sequence”. Two days prior to the hackathon the library was prepared and tested for functionality. The libraries can be stored at 4^o^C for up to 3 days.

***MinIONs in the classroom:***

In addition to the MkI MinIONs, ONT sponsored the class by supplying fresh flow cells a few days before the start of the hackathon. The MinION flow cells contain a port where the sample is applied. This port is the opening to a channel that pushes the applied reagents towards the pore-chamber. A new flow cell contains a small quantity of air at the opening that needs to be removed otherwise it will be pushed toward the pore-chamber and perturb the accessibility of the DNA strands to the pores. Removal of the air bubble requires attention and possibly help from instructors.

On the hackathon day we pre-prepared the fuel mix (required for priming the MinION flow cell) an hour before the hackathon start. The library mix was freshly prepared 10 minutes before loading of the samples.

***Computers and software***

*MinKnow*

Oxford Nanopore Technologies provided the class with 5 MkI MinIONs. The software interface MinKnow runs specifically on Windows computer systems at the time of writing. The Windows computer must have a USB 3.0 port and sufficient storage space for the data generated (we used up to 40 Gbytes). The computer requires installation of MinKnow and USB 3.0 driver. Running the MinION configuration menu in MinKnow tests correct installation of the software. MinKnow ran using the menu “MAP_48Hr_Sequencing_Run_SQK_MAP006”.

Computers should be able to run for up to 48 hours. We advise verification of the computer sleep-mode before starting a run.

*Basecalling*

Basecalling is performed via the cloud service Metrichor. Metrichor can be downloaded and installed on the workstation. Installation requires presetting the directory used for uploading raw squiggle data and downloading the base-called sequencing data. Moreover, the proper setting of the user key allows following of the yield of the sequencing run in real-time. For both hackathons the “2D basecalling for SQK-MAP006” recipe was used, which requires the base-called read to be 2D (i.e. successful passing of both template and complement) with an average Quality Score >9.

*Data Transfer*

The base-called data was synchronized to the student computers via BitTorrent. Selecting a folder creates a folder-specific key, which can be provided to the students. Via a manual connection on the personal laptop, the student can paste the key. This instantly starts synchronization between workstation and personal laptop. Before the hackathon it is advisable to notify students that they require a minimum of ~20 GB of storage on their computers (this number depends on the productivity of the flow cell, the DNA library and the duration of the run). A possible alternative is the use of an external hard drive. During both hackathons, one computer would not connect to the MinION and one computer stopped generating data after going into sleep mode. These issues should be considered when setting up the operating system.

Copyright:

© 2016 Zaaijer et al. This teaching material is provided under the Creative Commons Attribution-Share Alike 4.0 International License
