# Supplementary material for: Using mobile sequencers in an academic classroom
Source: eLife. 2016 Apr 7;5:e14258. doi: 10.7554/eLife.14258 (PMC4869913; doi:10.7554/eLife.14258)
Supplement: Supplementary file 3. — DOI: http://dx.doi.org/10.7554/eLife.14258.007 [file elife-14258-supp3.docx]

**Supplemental Note 3**

Hackathon #1 | Snack to Sequence

Please **read** and **follow** these instructions:

**October 25^th^ 11:59pm:** Email [szaaijer@nygenome.org](mailto:szaaijer@nygenome.org) to notify that you were able to synchronize you computer using BitTorrent.

**October 30^th^ Noon:** first submission ‘Quality Assessment MinION reads’.

**November 6^th^** **Noon:** second submission ‘Snack to sequence pipeline’.

**November 6^th^**: presentations ‘Snack to sequence’. Each team will have 10 min to present. Two presenting members will be selected randomly at the beginning of class.

**For both submissions submit to** szaaijer@nygenome.org and yaniv@cs.columbia.edu with subject line [Hackathon#1, assignment number, groupID]

- Submit the written portion of your homework.
- Submit all code written by the group in their original extensions. Any programming language is acceptable, although Python is preferred.
- Your code should have a [GPLv2](https://www.gnu.org/licenses/old-licenses/gpl-2.0.txt) license.
- Submission can be done by emailing a link to a git repo. If the team does not know how to use git, they can email a tar ball of the code.

**Late submission policy:**

Failures to meet deadlines will result in 25% grade reduction for each late day.

**Assignment 1** Quality Assessment MinION reads

Convert the data from fast5 files to fasta files and fastq using ‘Poretools’.

Analyze the following parameters:

1 Calculate the number of 1D and 2D reads classified as ‘failed’ versus the number of 1D and 2D reads classified as ‘passed’. Calculate the fraction of reads that are 2D called in both the ‘pass’ and ‘fail’ folders.

2 Calculate average reads per active channel (remember you wrote the number of active pores down for group 1 during the hackathon). Which channel in the flowcell produced the most reads? How many?

3 Plot the cumulative nucleotides sequenced as a function of time for both ‘failed’ and ‘passed’ 2D reads in separate graphs.

4 How many hours would you have to sequence in order to cover the human genome once? (Only using 2D reads that passed the quality filters.)

5 Metrichor uses a base-calling algorithm that gives the accuracy with which the sequencing platform could identify the particular base (FASTQ files include quality scores). The quality scores are based on ASCII, which is a character encoding system that maps a number to a character. Calculate the base-calling quality mean and standard deviation for 2D reads for both the ‘failed’ and ‘passed’ reads, and compare using a student’s t-test.

6 In addition, compare the median base quality for the ‘passed’ 2D reads from the first hour with the median base quality of the last hour of that same sequencing run. Briefly comment on your results.

7 Plot a histogram the length distribution of 1D reads (template and complement) and the 2D reads in the failed folder. Do the same for the ‘passed’ reads.

8 Identify the longest read you obtained for: template, complement, and 2D from the passed reads. State the number of nucleotides for each.

9 Analyze whether there is a correlation between sequence length and timing of a DNA strand passing through the pore. Plot the obtained sequence length over time for 2D reads. Briefly comment on your results. Plot the pace of the strand sequencing (sequence length per duration in pore) for 2D reads classified as ‘failed’ versus reads classified as ‘passed’.

10 Define the nucleotide composition of both 2D sequences classified as ‘passed’ and as ‘failed’ (calculate the percentage of G,C,T, and As in the results).

11 Build a model that predicts the time to sequence a segment based on the input sequence. You can use any type of classifier or features you want. Report the cross correlation r^2.

Your submitted code should be able to replicate the output of your report. Document your code. You can write a separate program for each question. The naming of your code should be groupX_report1_questionY, where X is your group number and Y is the question number

**Assignment 2** Snack to sequence pipeline

1 Develop an analysis pipeline for:

- - Identification of food ingredients in your sample.
  - Once you identified the ingredients, quantify the ratios in your dish.
  - Do you find any bacteria?

*Bonus points* if you can develop a simple web interface/app that takes MinION reads and generates a visual real time analysis.

2 After how many minutes in a MinION run would you be able to state what the composition of your food was and in what ratio?

3 Filter the sequences for one of the food components.

- From those sequences, make a confusion matrix. A confusion matrix takes a known reference sequence, and tests the classification of your reads.
- Based on the alignments obtained, filter only the deletions and insertions. Of the deletions and insertions found, calculate the size distribution and the nucleotide composition.

**Presentation:** Snack to sequence pipeline

The presentation should include the following items:

1. Report the output of the sequencer and the longest read.
2. Present the number of errors and quality of the sample.
3. Present the classifier and features of reading speed and performance.
4. Strategy for identifying Sophie’s food.
5. What biological ingredients you find in her food.
6. Suggestion for a follow up question.

Copyright:

© 2016 Zaaijer et al. This teaching material is provided under the Creative Commons Attribution-Share Alike 4.0 International License
