# Supplementary material for: Using mobile sequencers in an academic classroom
Source: eLife. 2016 Apr 7;5:e14258. doi: 10.7554/eLife.14258 (PMC4869913; doi:10.7554/eLife.14258)
Supplement: Supplementary file 4. — DOI: http://dx.doi.org/10.7554/eLife.14258.008 [file elife-14258-supp4.docx]

**Supplemental Note 4**

Hackathon #2 | CSI Columbia

**Assignment 3: QC and alignment**

1    Calculate the number of 1D and 2D reads classified as ‘failed’ versus the number of 1D and 2D reads classified as ‘passed’. Calculate the fraction of reads that are 2D called in both the ‘passed’ and ‘failed’ folders.

2    Calculate average reads per active channel (remember you wrote the number of active pores down for group 1 during the hackathon). Which channel in the flow-cell produced the most reads? How many?

3    Plot the cumulative nucleotides sequenced as a function of time for both ‘failed’ and ‘passed’ 2D reads in separate graphs.

4 Plot a histogram of the length distribution of 1D reads (template and complement) and the 2D reads in the failed folder. Do the same for the ‘passed’ reads.

5    Identify the longest read you obtained for: template, complement, and 2D from the passed and failed reads. State the number of nucleotides for each.

6 Align the reads to the human genome (hg19) using BWA-MEM ONT. Report how many reads aligned.

7 Report a confusion matrix of your reads.

8 Open question: suggest three strategies to reduce the number of errors in the reads.

Your submitted code should be able to replicate the output for your report. Document your code. You can write a separate program for each question. The naming of your code should be groupX_report1_questionY, where X is your group number and Y is the number of question (or questions).

**Assignment 4**

Try to find as much as you can about the person you sequenced:

- Who is this person?
- Ancestry?
- Sex?
- Phenotypic traits?
- Go wild!

Helpful links:

- <https://genome.ucsc.edu/>
- <http://www.1000genomes.org/>
- <https://imputationserver.sph.umich.edu/index.html>
- <http://pritchardlab.stanford.edu/structure.html>
- <https://opensnp.org/>

**Presentation:** CSI Columbia

The presentation should include the following items:

1. Report the output of the sequencer and the longest read.
2. Present QC of the data.
3. Present the error frequency, and confusion matrix.
4. Present the pipeline used to identify the traits.
5. Present all traits you could find.

Copyright:

© 2016 Zaaijer et al. This teaching material is provided under the Creative Commons Attribution-Share Alike 4.0 International License
