# Supplementary material for: Using mobile sequencers in an academic classroom
Source: eLife. 2016 Apr 7;5:e14258. doi: 10.7554/eLife.14258 (PMC4869913; doi:10.7554/eLife.14258)
Supplement: Supplementary file 5. — DOI: http://dx.doi.org/10.7554/eLife.14258.009 [file elife-14258-supp5.docx]

**Supplemental Note 5**

**Final project:**

Task: Suggest a potential application for mobile sequencers

Your essay should include the following sections:

1. TL;DR: summarize your idea in <140 characters.
2. Introduction: what is the need and how it is currently done.
3. Your idea (can be a service, device, application, etc…). Be creative!
4. Potential engineering challenges to overcome.
5. Summary.

Mechanics:

- Groups of two students
- Include 5-10 references (not included in word limit)
- One figure that presents your idea graphically (e.g. cartoon).
- Length: 1,200-1,500 words.

Grading is based on your creativity, imagination, clearness, understatement of the need, and understanding the technical challenges.

Copyright:

© 2016 Zaaijer et al. This teaching material is provided under the Creative Commons Attribution-Share Alike 4.0 International License
