# Supplementary material for: Using mobile sequencers in an academic classroom
Source: eLife. 2016 Apr 7;5:e14258. doi: 10.7554/eLife.14258 (PMC4869913; doi:10.7554/eLife.14258)
Supplement: Supplementary file 6. — DOI: http://dx.doi.org/10.7554/eLife.14258.010 [file elife-14258-supp6.pptx]

## Slide 1
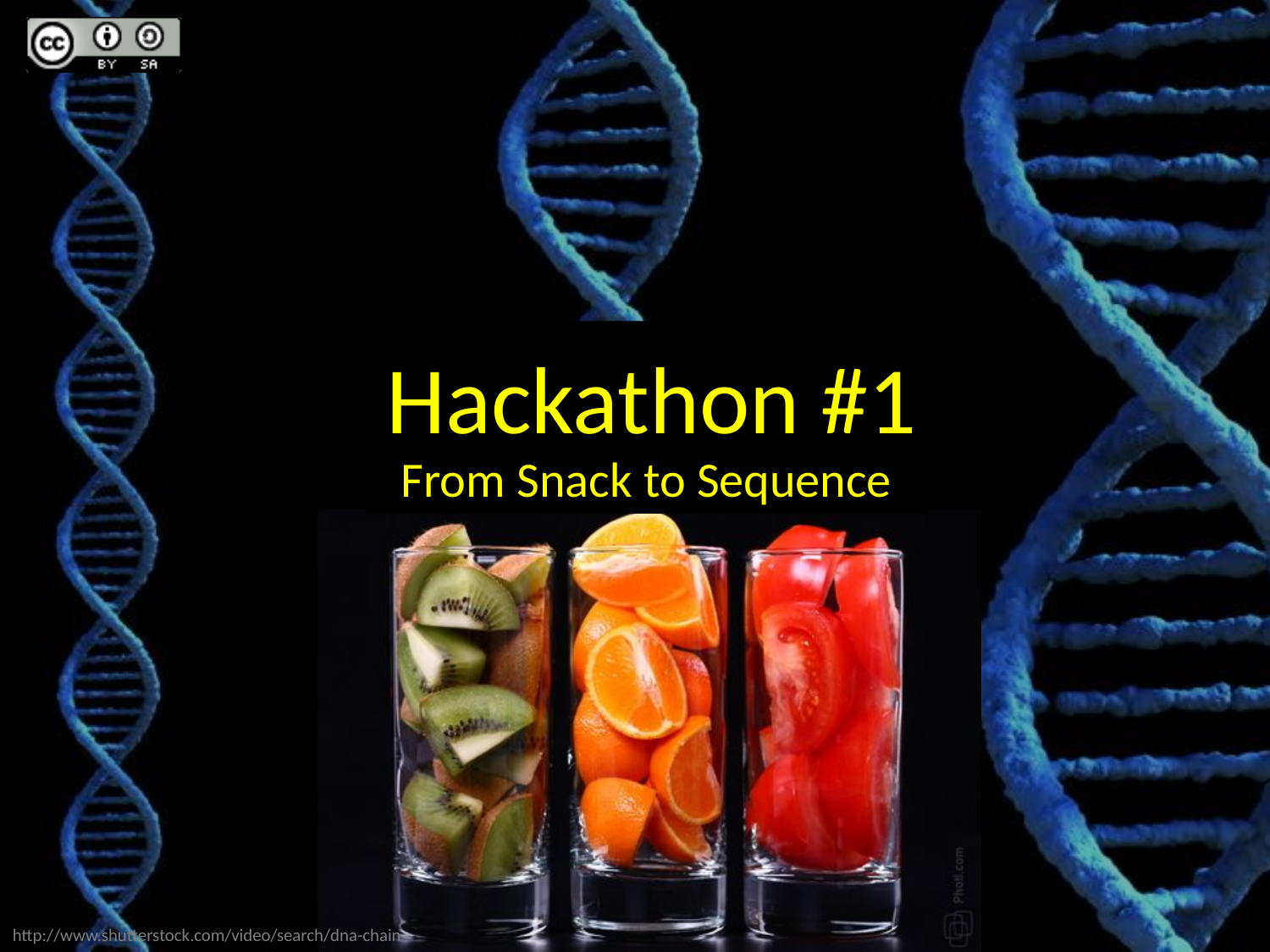

# Hackathon #1
From Snack to Sequence
http://www.shutterstock.com/video/search/dna-chain

## Slide 2
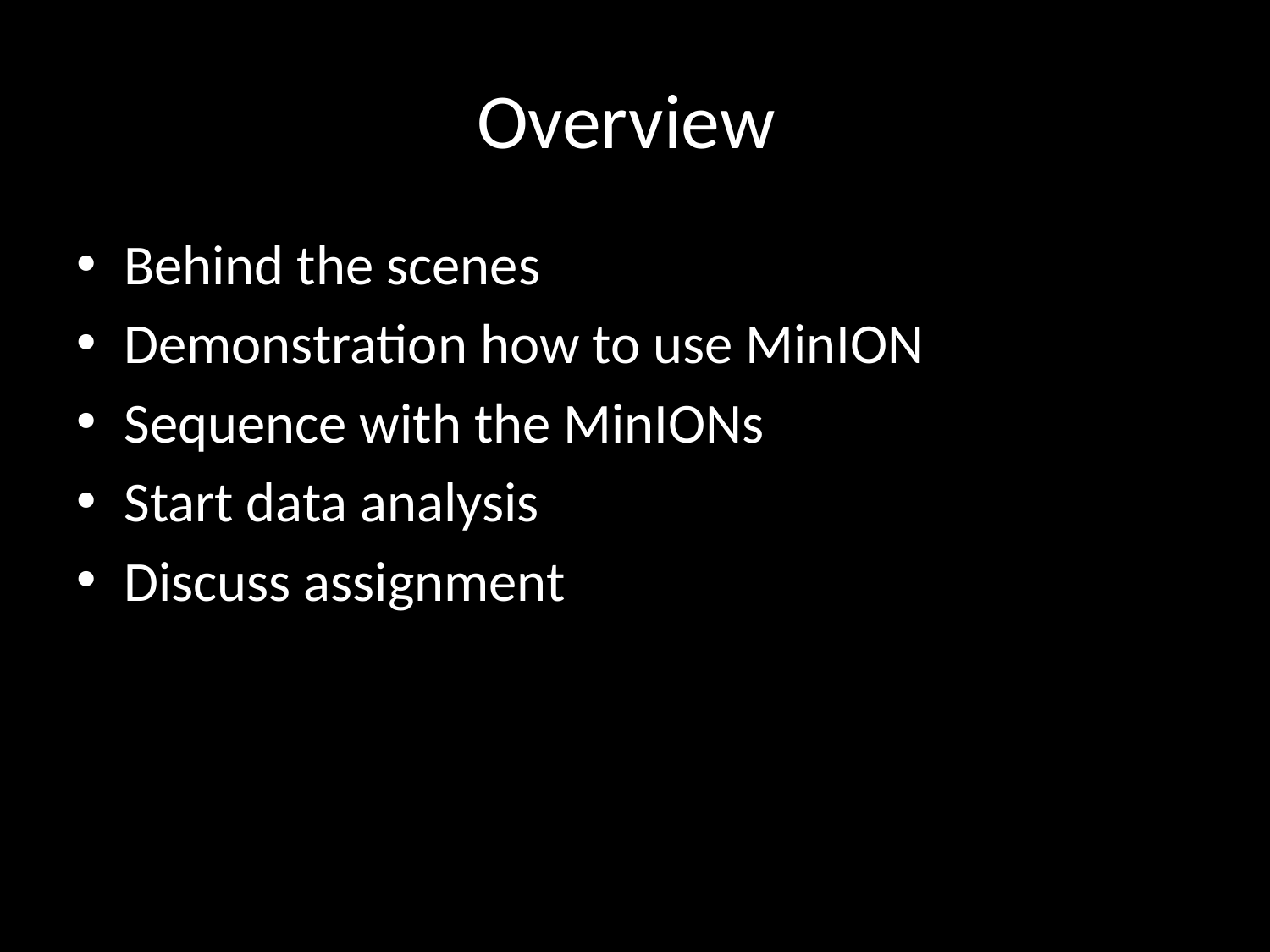

# Overview
Behind the scenes
Demonstration how to use MinION
Sequence with the MinIONs
Start data analysis
Discuss assignment

## Slide 3
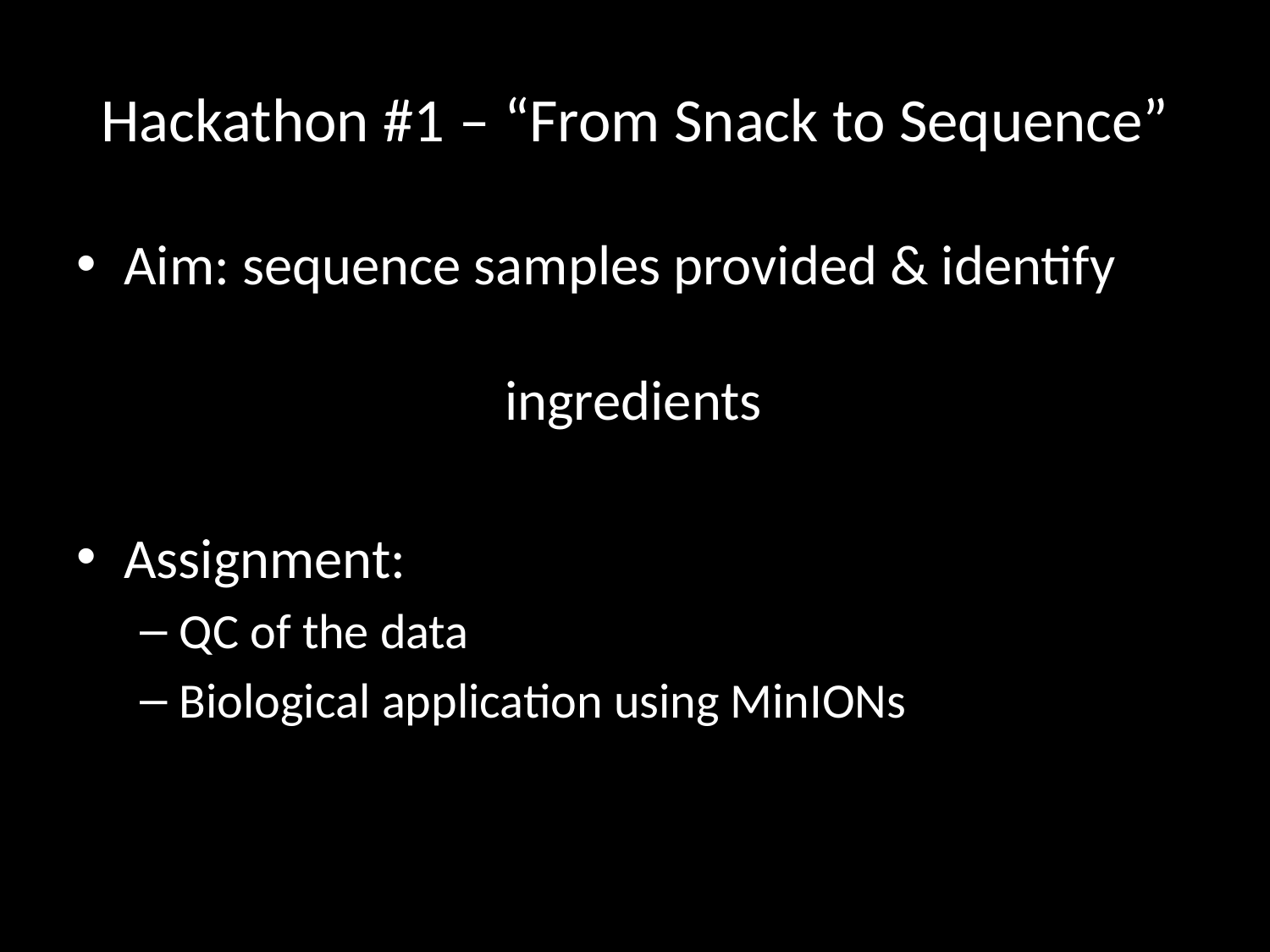

# Hackathon #1 – “From Snack to Sequence”
Aim: sequence samples provided & identify 												ingredients
Assignment:
QC of the data
Biological application using MinIONs

## Slide 4
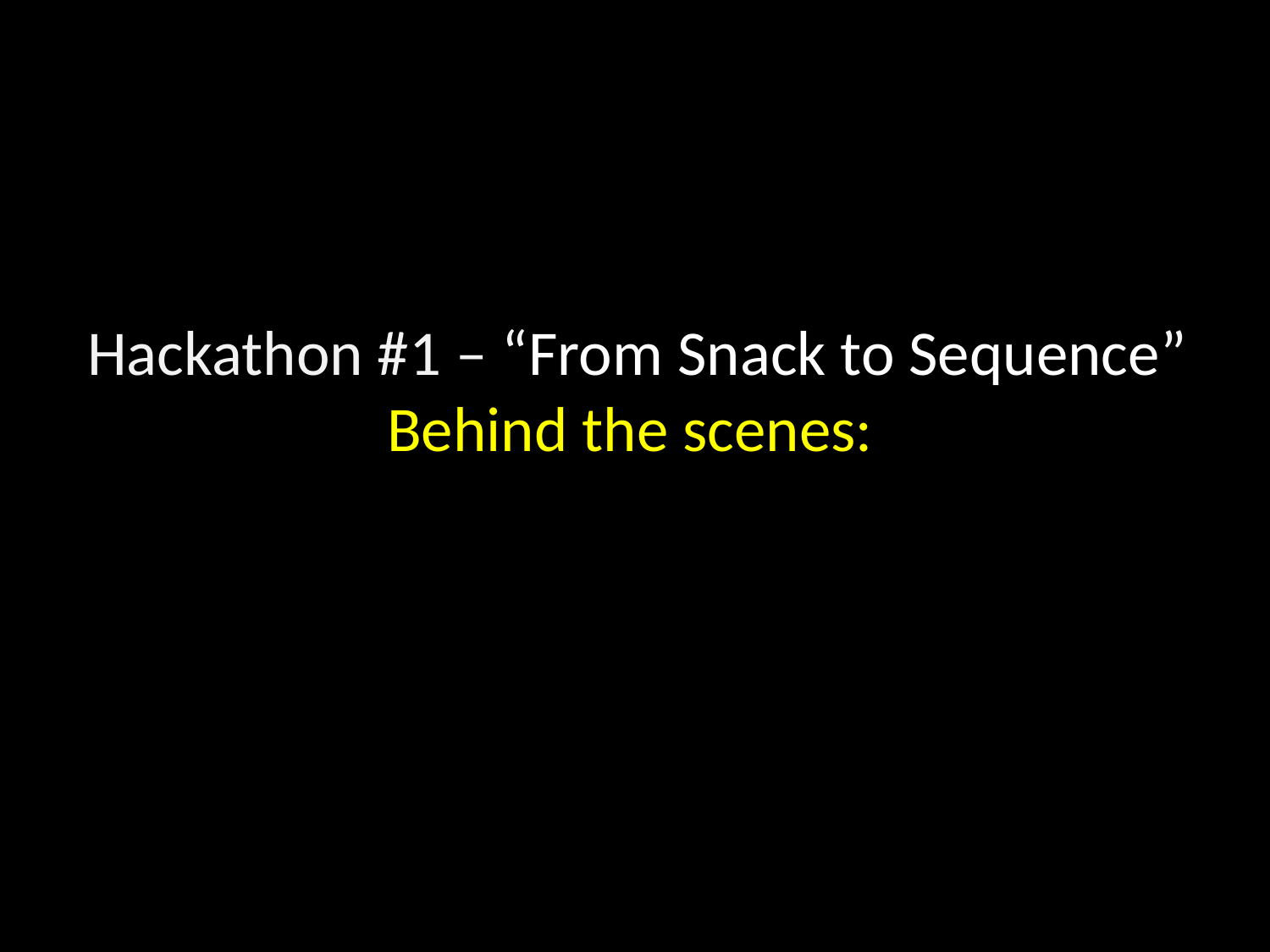

# Hackathon #1 – “From Snack to Sequence” Behind the scenes:

## Slide 5
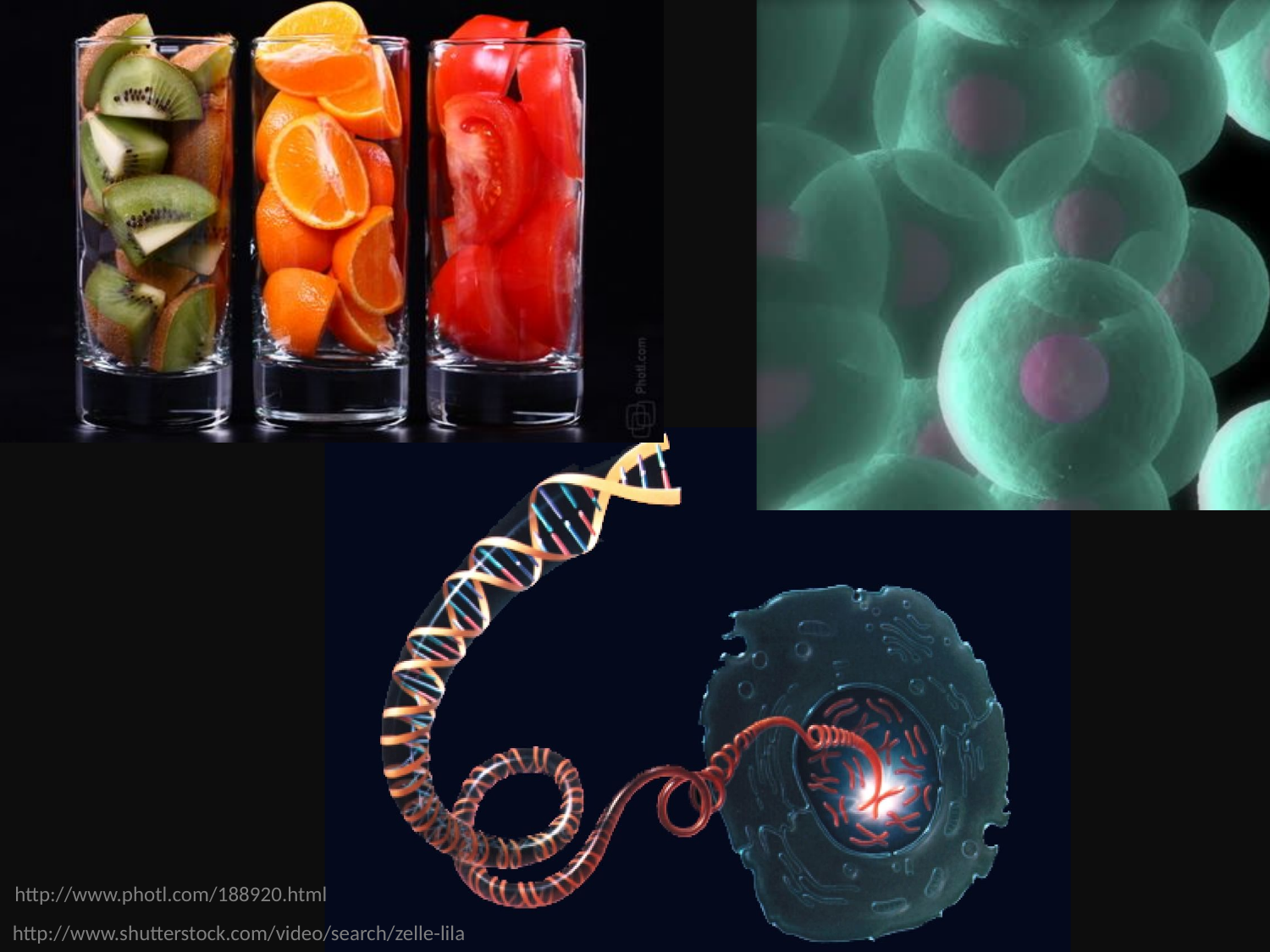

http://www.photl.com/188920.html
http://www.shutterstock.com/video/search/zelle-lila

## Slide 6
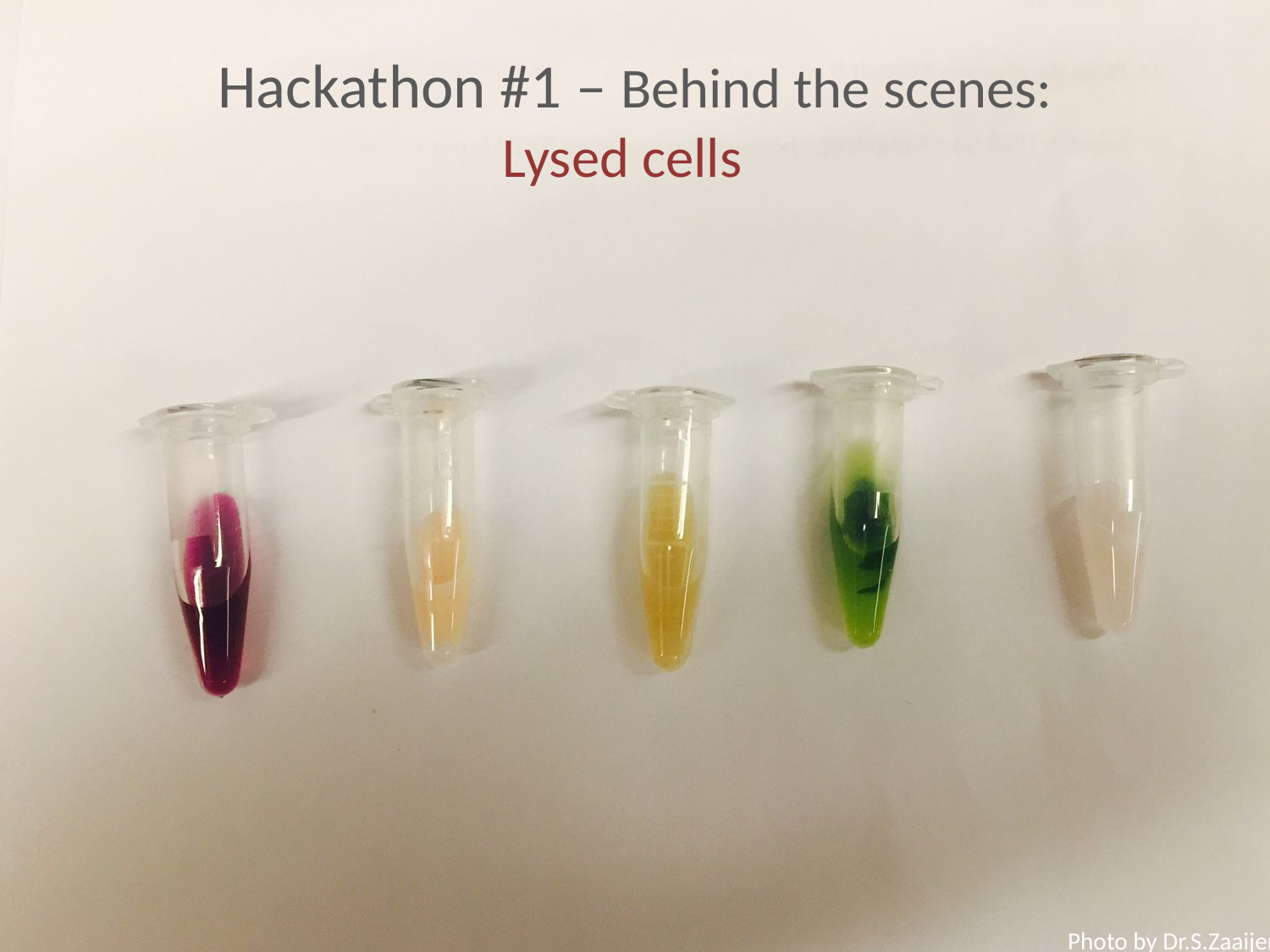

# Hackathon #1 – Behind the scenes: Lysed cells
Photo by Dr.S.Zaaijer

## Slide 7
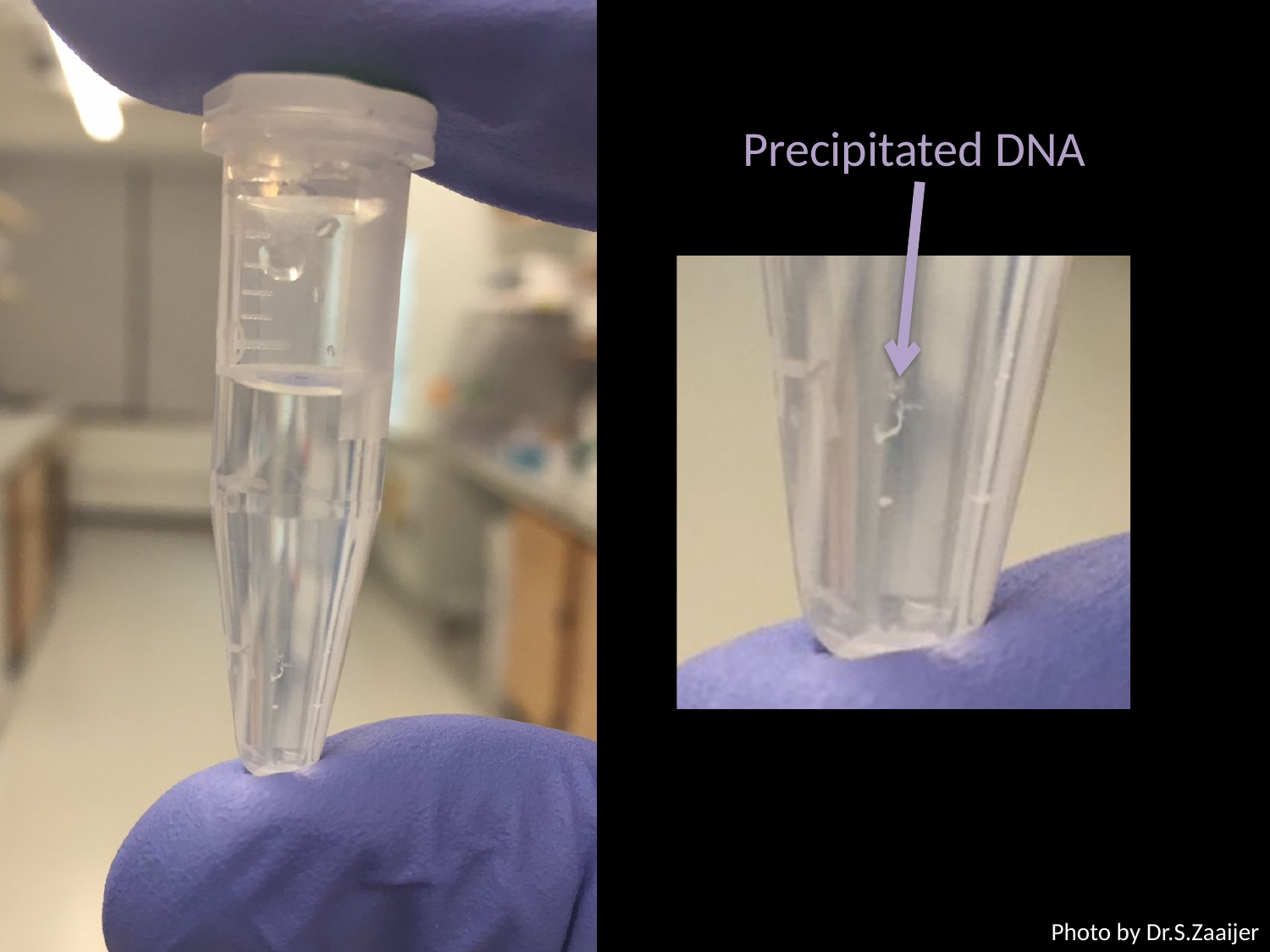

Precipitated DNA
Photo by Dr.S.Zaaijer

## Slide 8
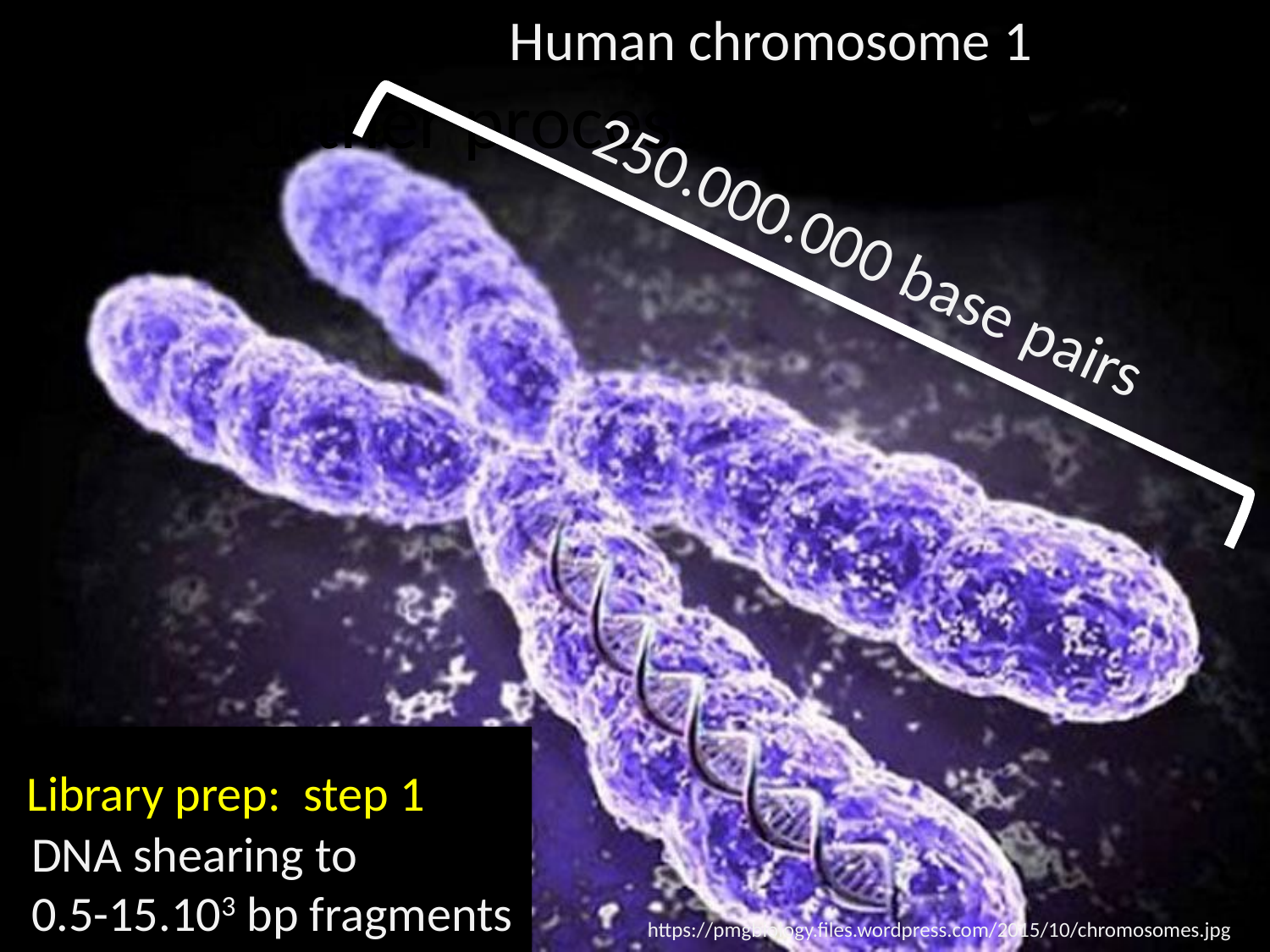

Human chromosome 1
# Further processing of DNA
250.000.000 base pairs
Library prep: step 1
DNA shearing to
0.5-15.103 bp fragments
https://pmgbiology.files.wordpress.com/2015/10/chromosomes.jpg

## Slide 9
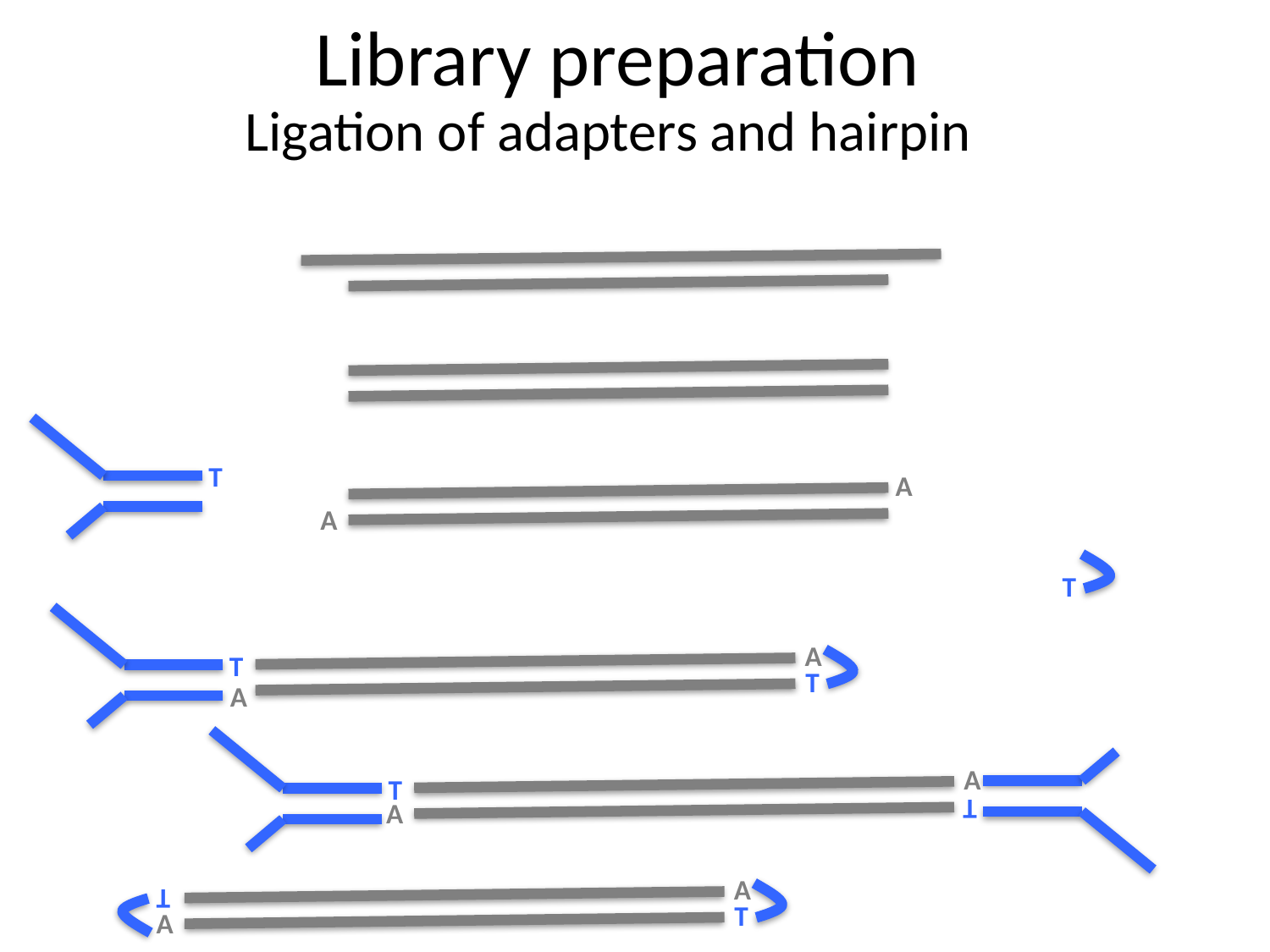

# Library preparation
Ligation of adapters and hairpin
T
A
A
T
T
A
T
A
T
T
A
A
A
T
T
A

## Slide 10
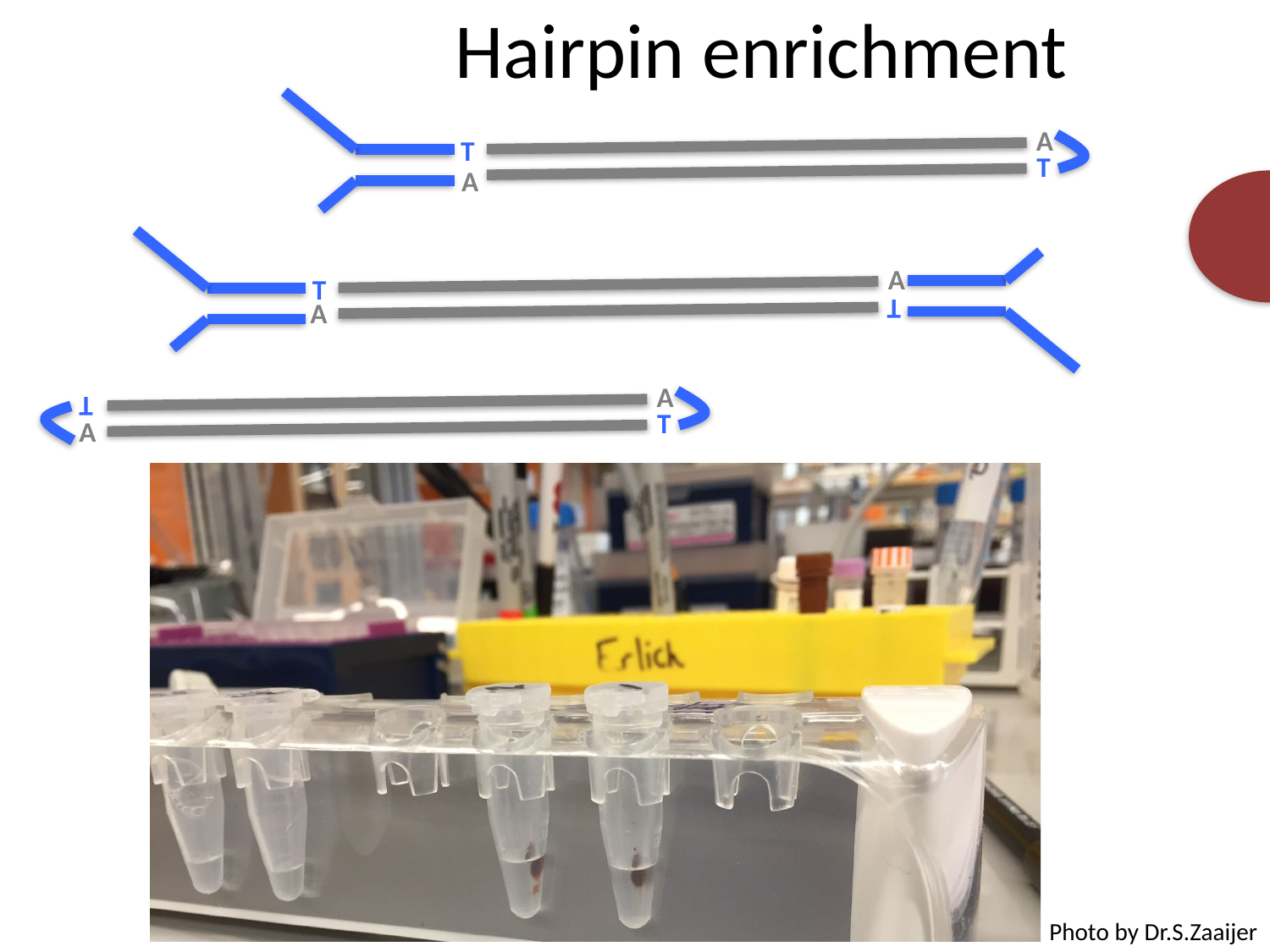

# Hairpin enrichment
T
A
T
A
T
T
A
A
A
T
T
A
Photo by Dr.S.Zaaijer

## Slide 11
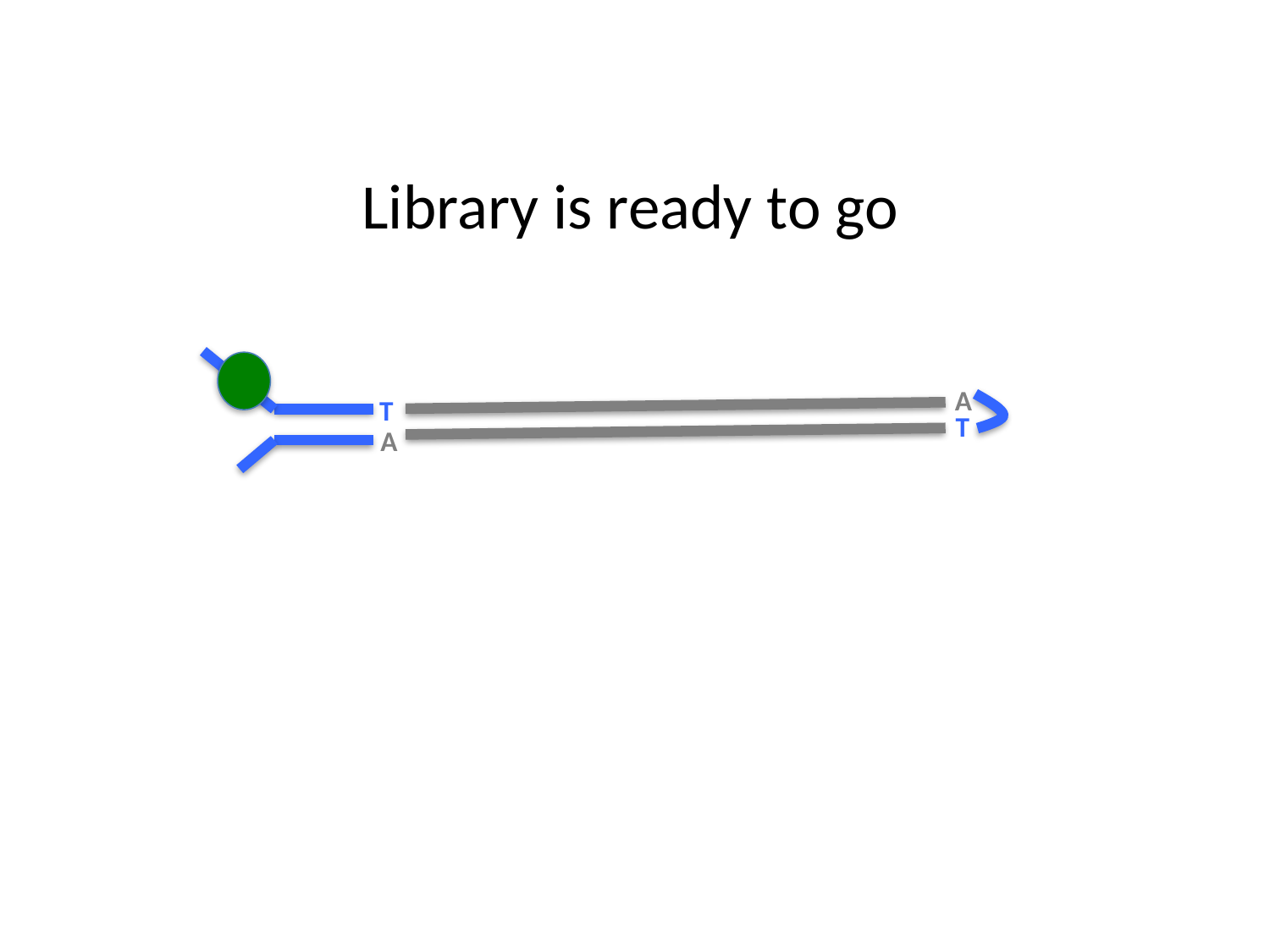

Library is ready to go
T
A
T
A

## Slide 12
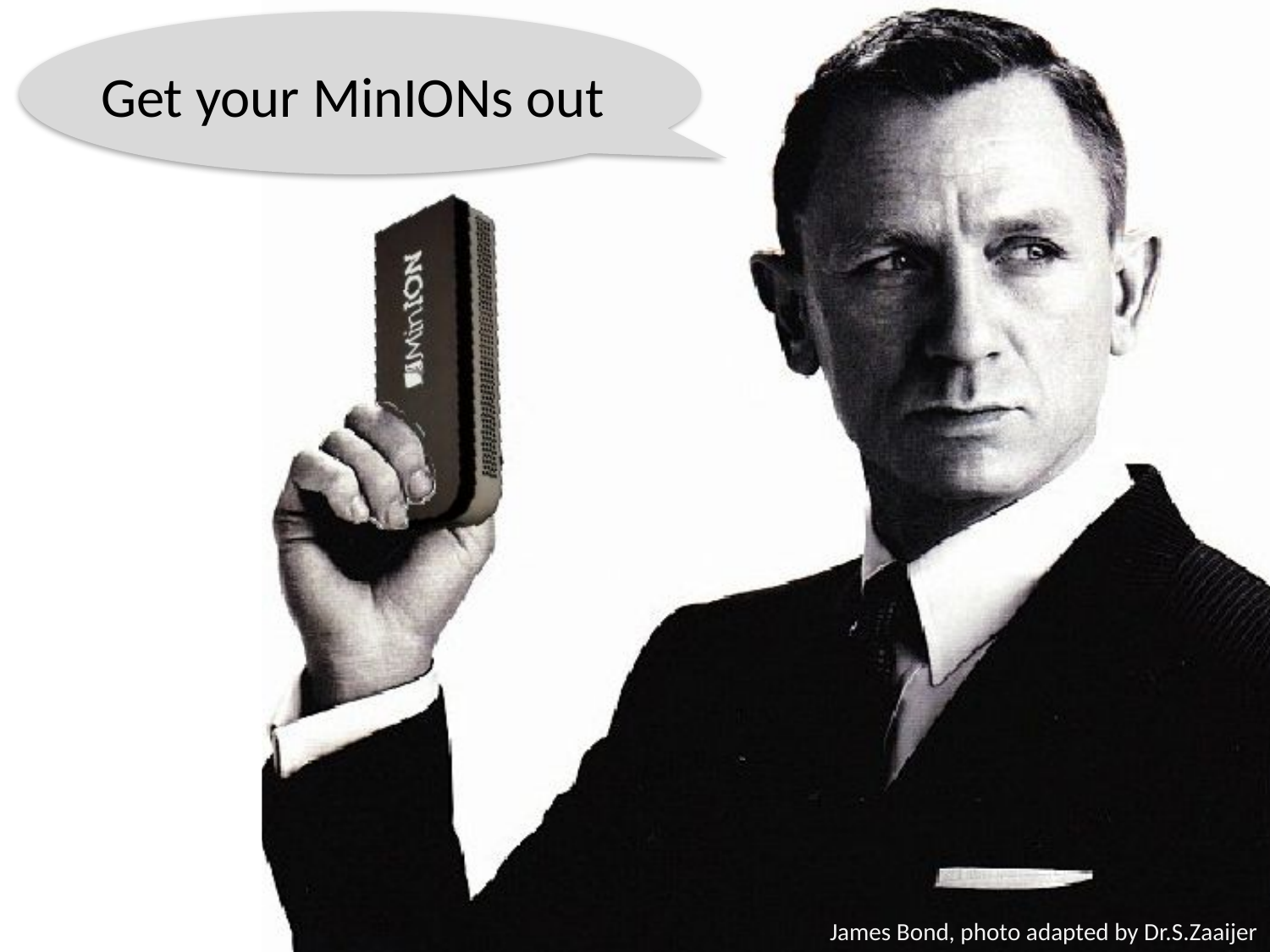

# Get your MinIONs out
James Bond, photo adapted by Dr.S.Zaaijer

## Slide 13
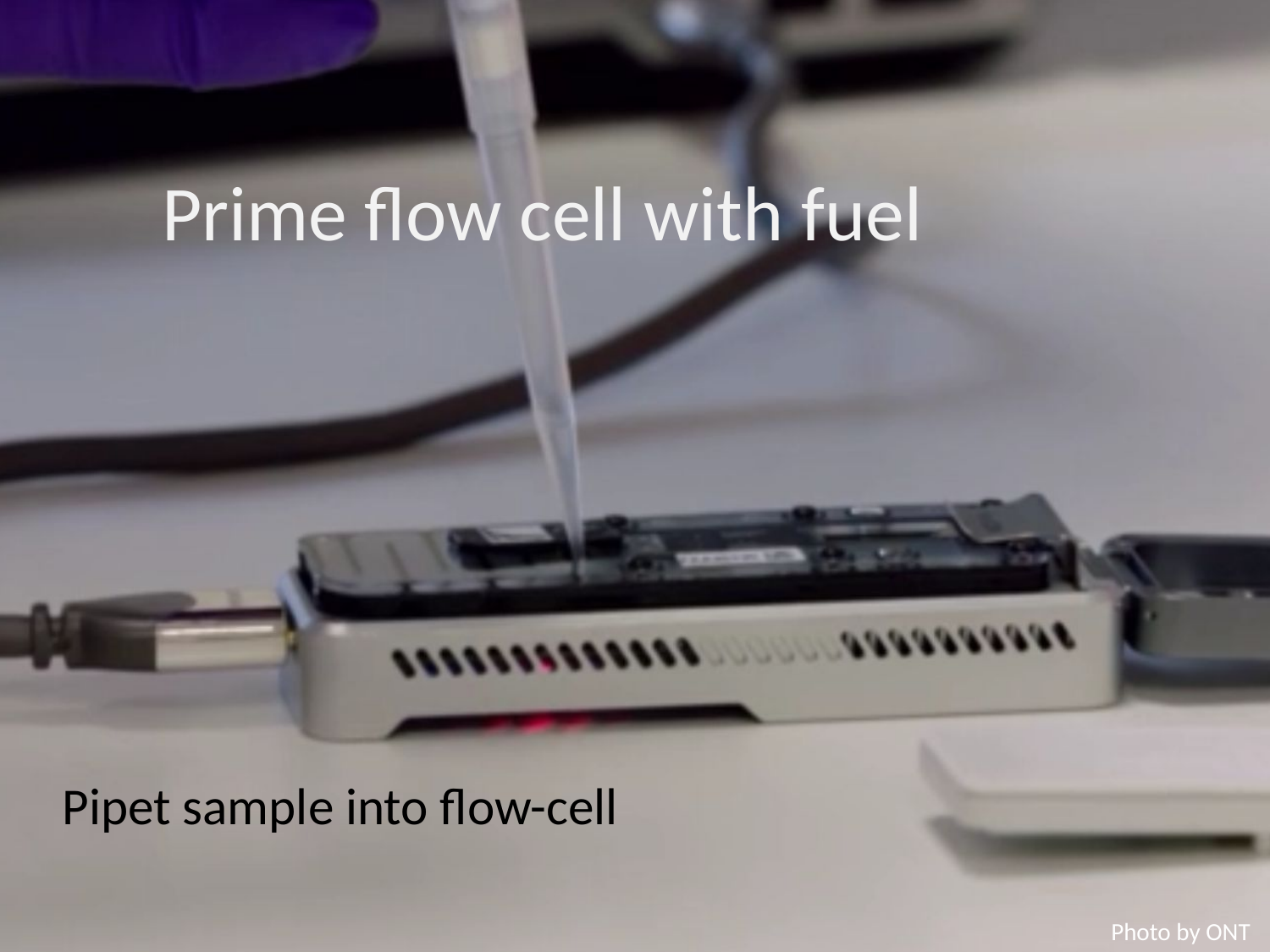

Prime flow cell with fuel
Pipet sample into flow-cell
Photo by ONT

## Slide 14
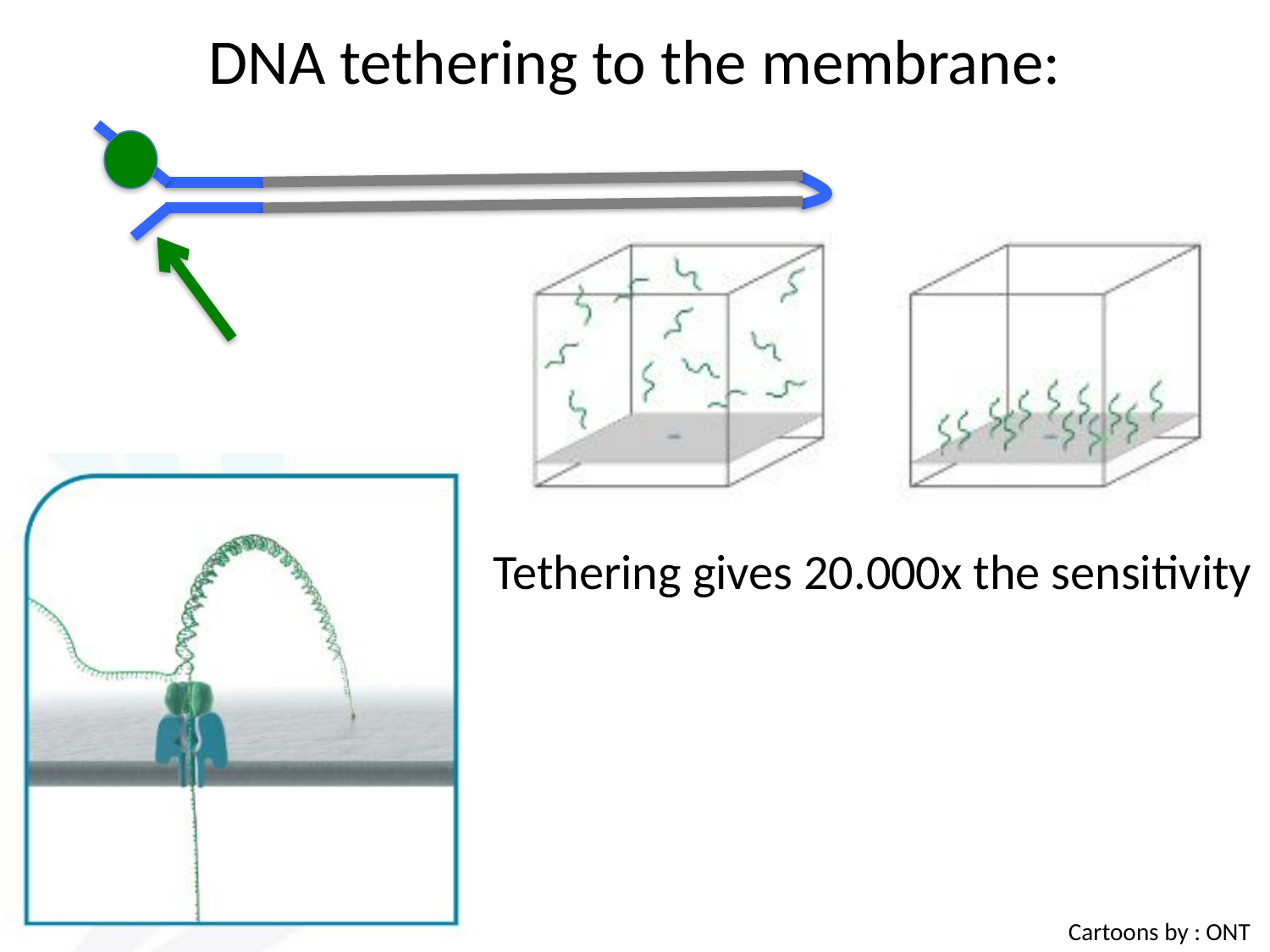

# DNA tethering to the membrane:
Tethering gives 20.000x the sensitivity
Cartoons by : ONT

## Slide 15
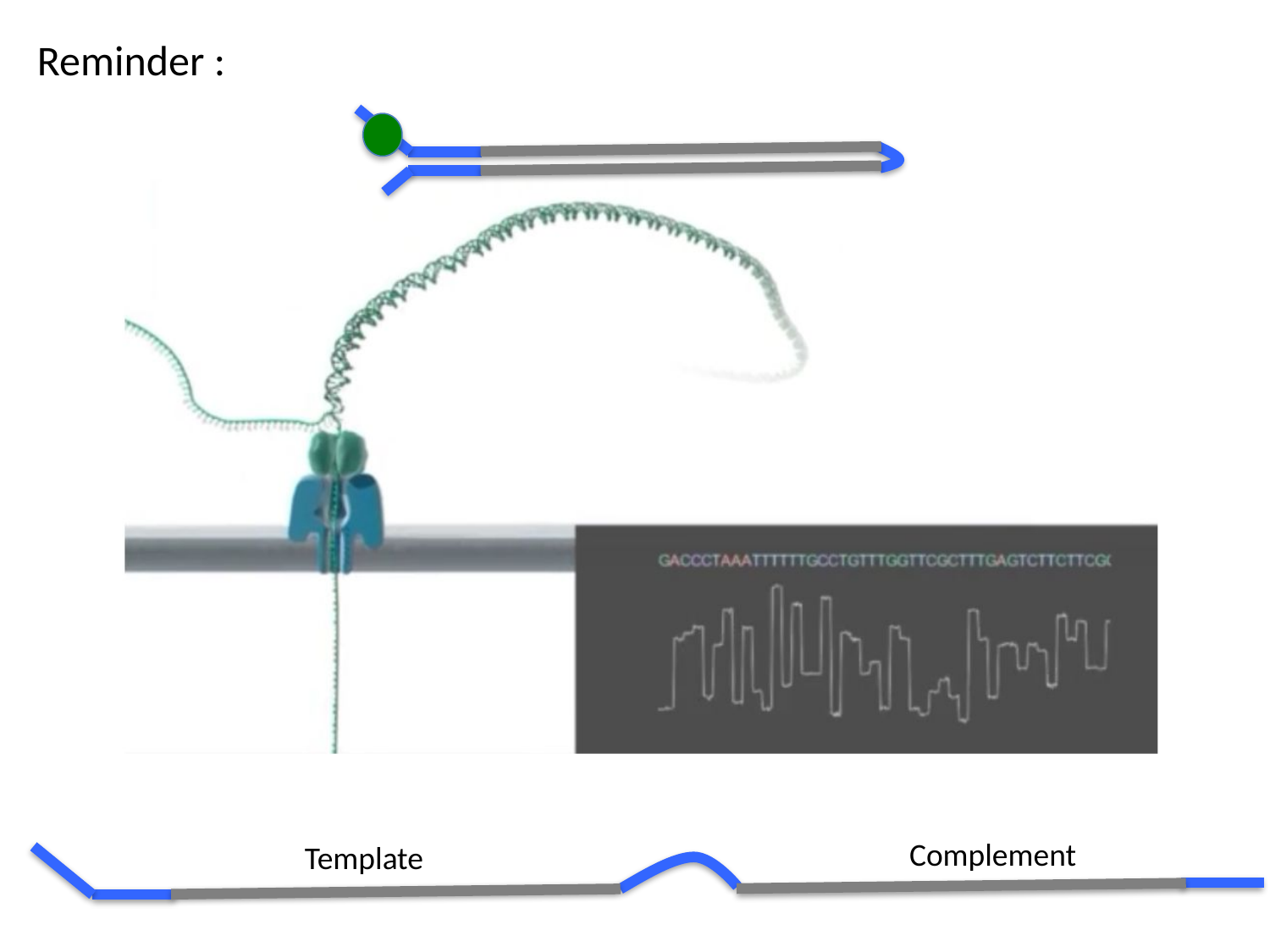

Reminder :
Complement
Template

## Slide 16
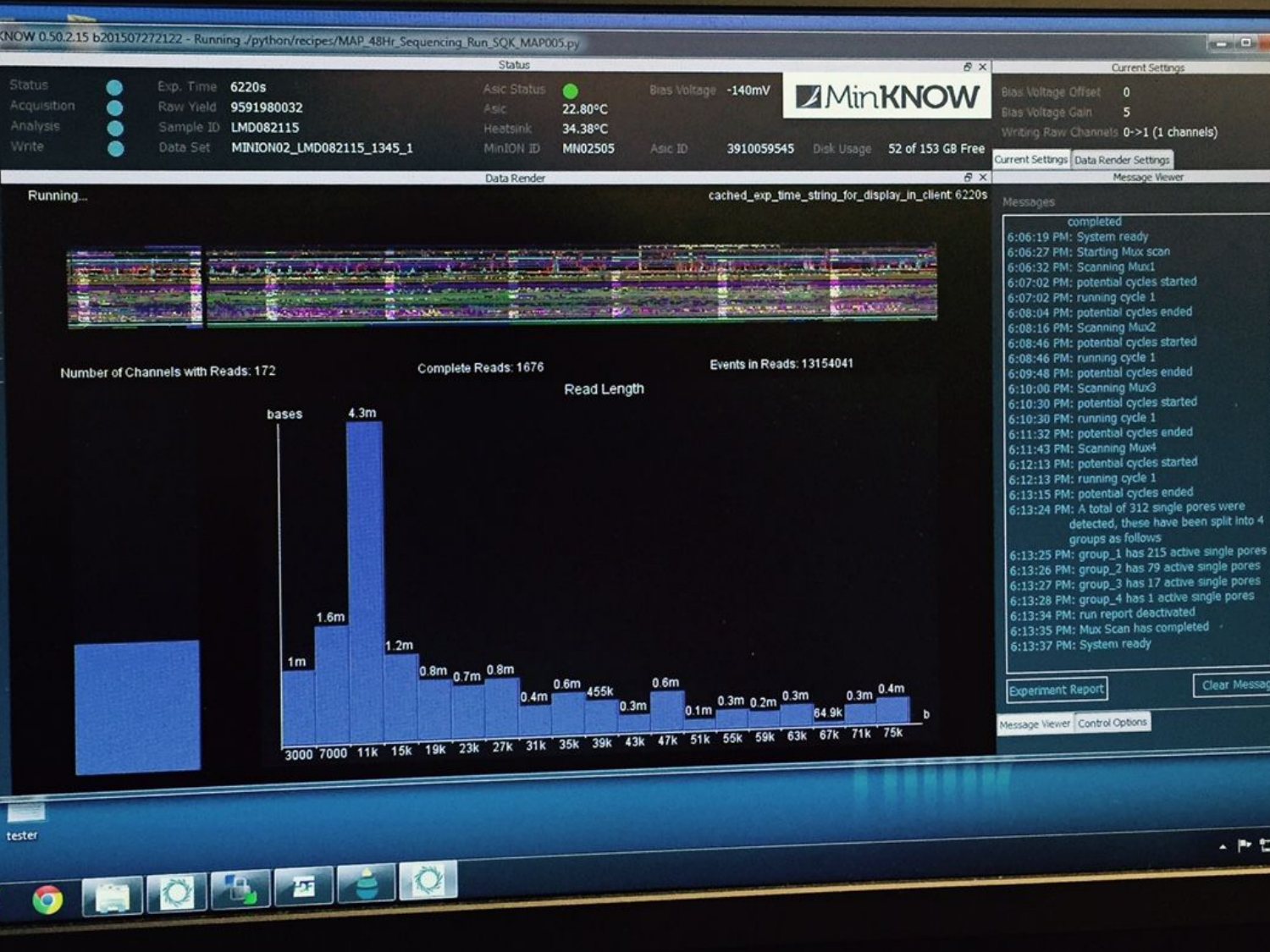

## Slide 17
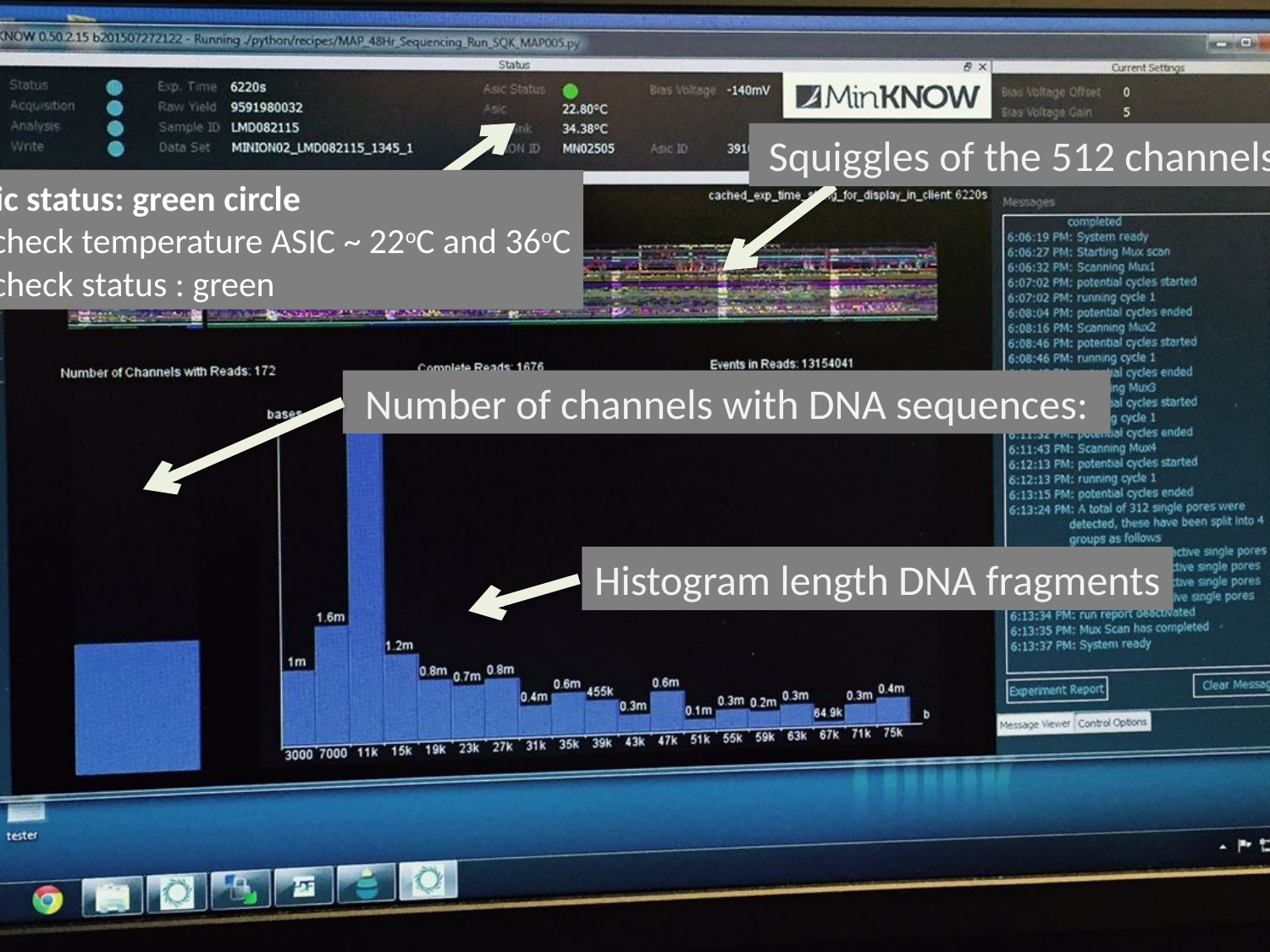

Squiggles of the 512 channels
Check Asic status: green circle
	check temperature ASIC ~ 22oC and 36oC
	check status : green
 Number of channels with DNA sequences:
Histogram length DNA fragments

## Slide 18
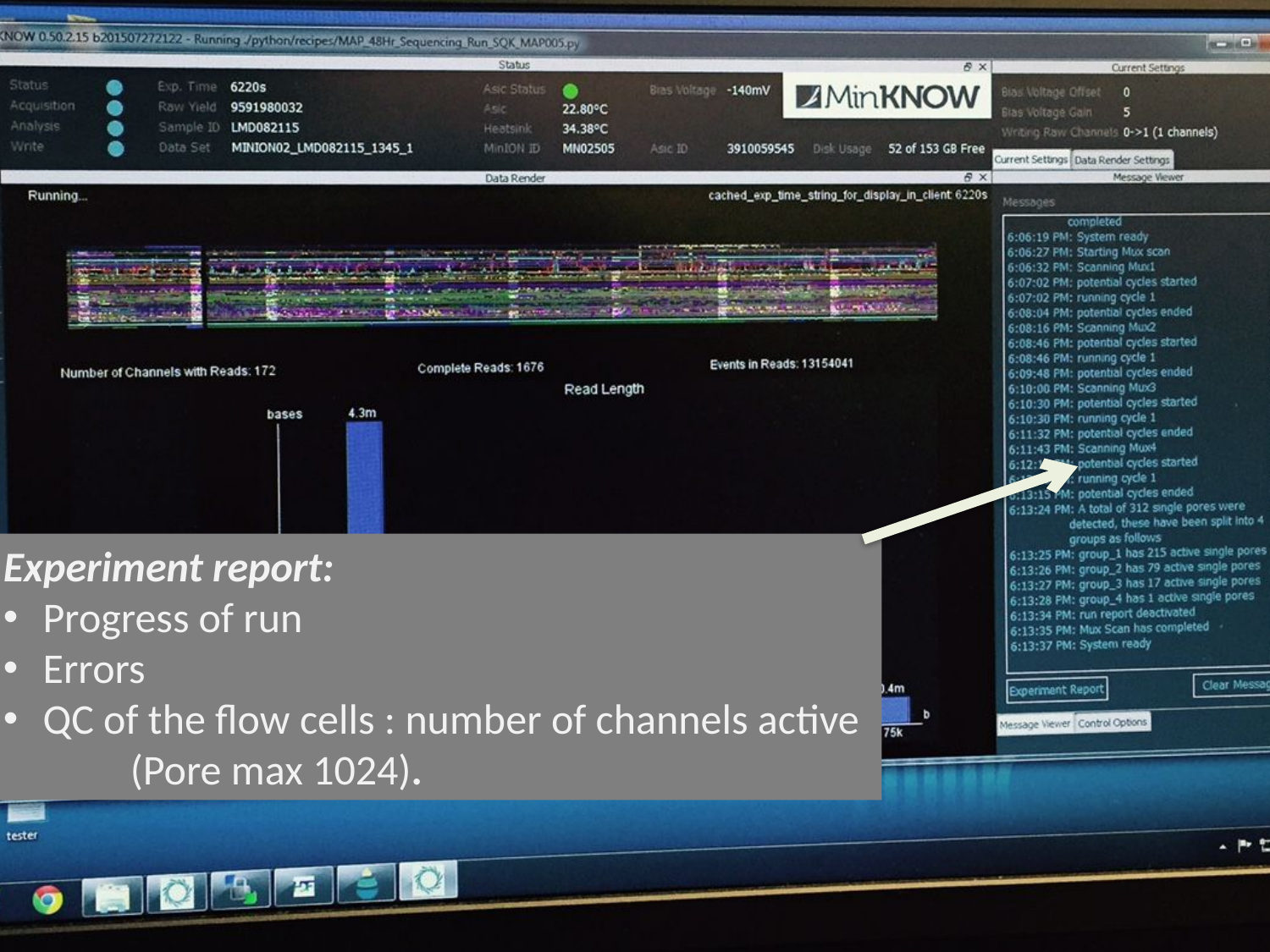

Experiment report:
Progress of run
Errors
QC of the flow cells : number of channels active
	(Pore max 1024).

## Slide 19
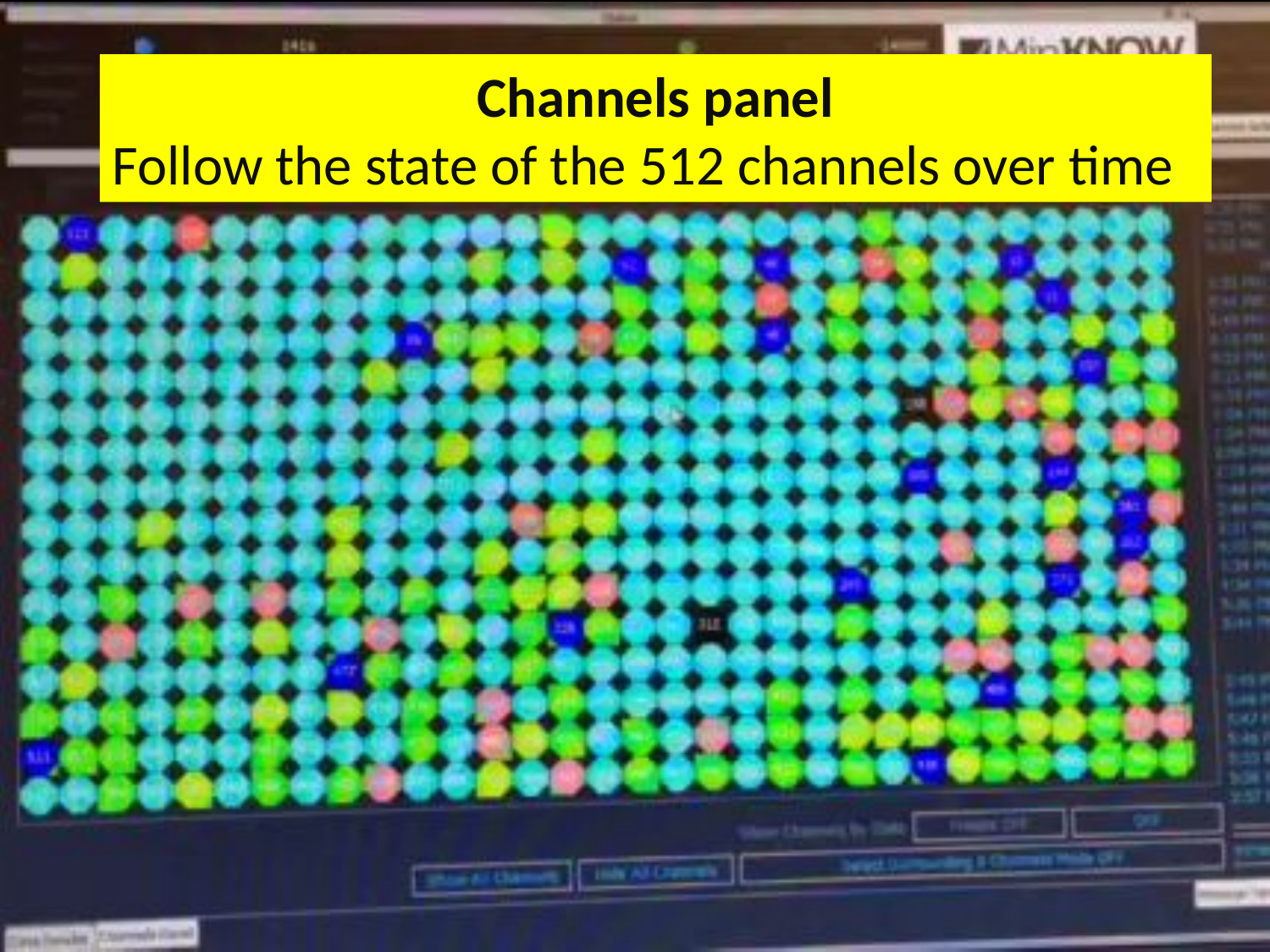

# minKNOW
Channels panel
Follow the state of the 512 channels over time

## Slide 20
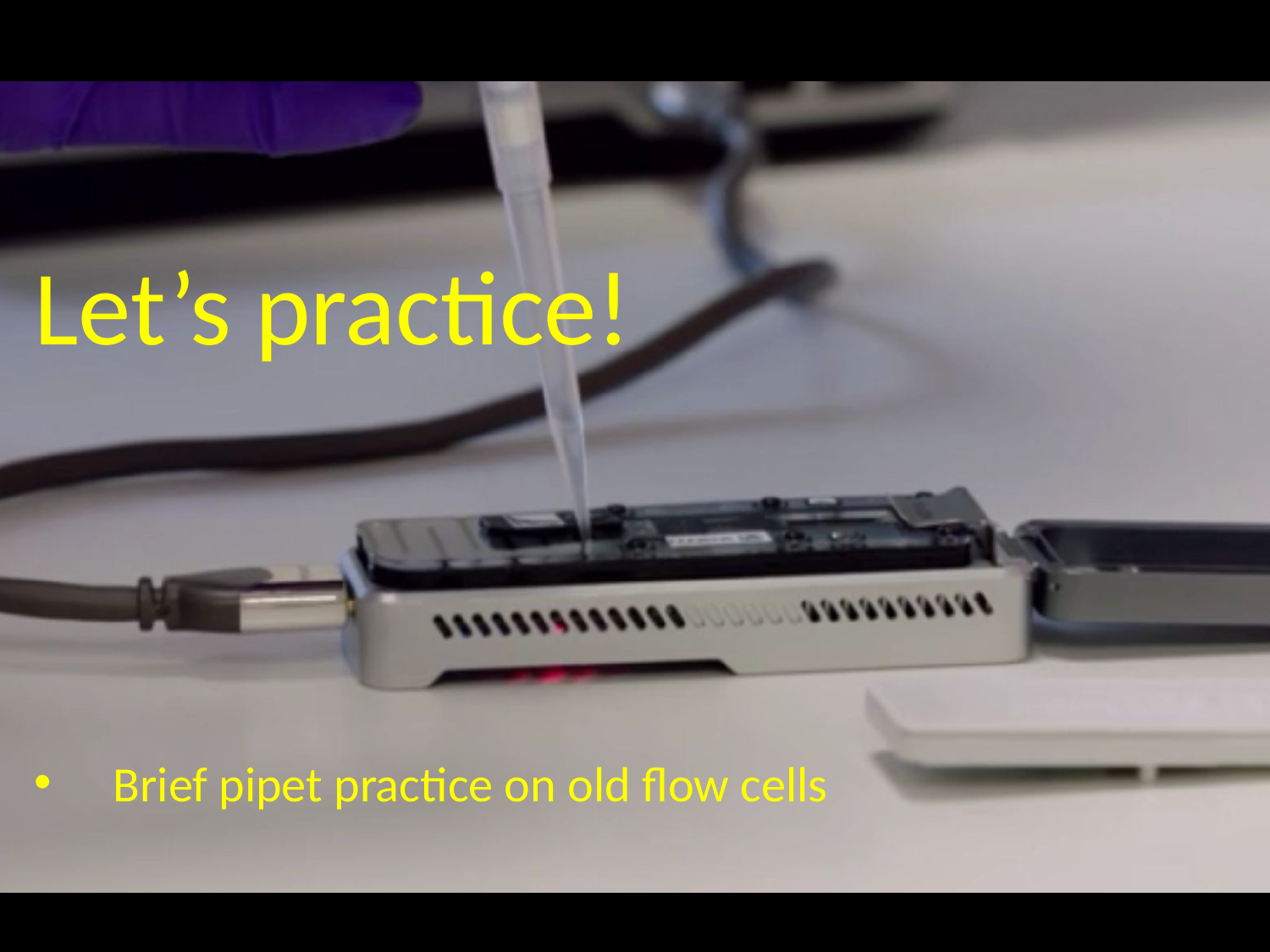

# Let’s practice!
Brief pipet practice on old flow cells

## Slide 21
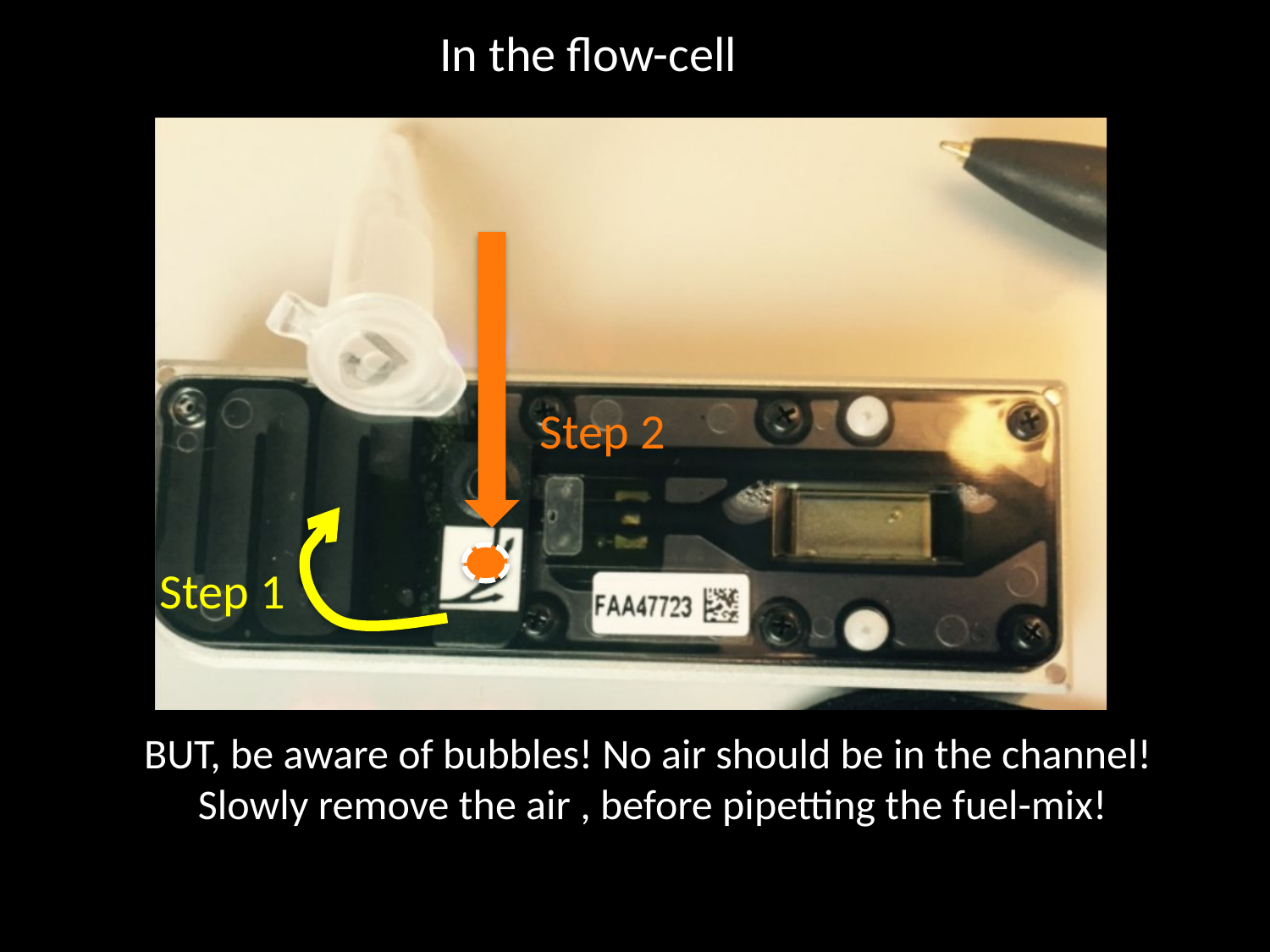

In the flow-cell
Step 2
Step 1
BUT, be aware of bubbles! No air should be in the channel!
Slowly remove the air , before pipetting the fuel-mix!

## Slide 22
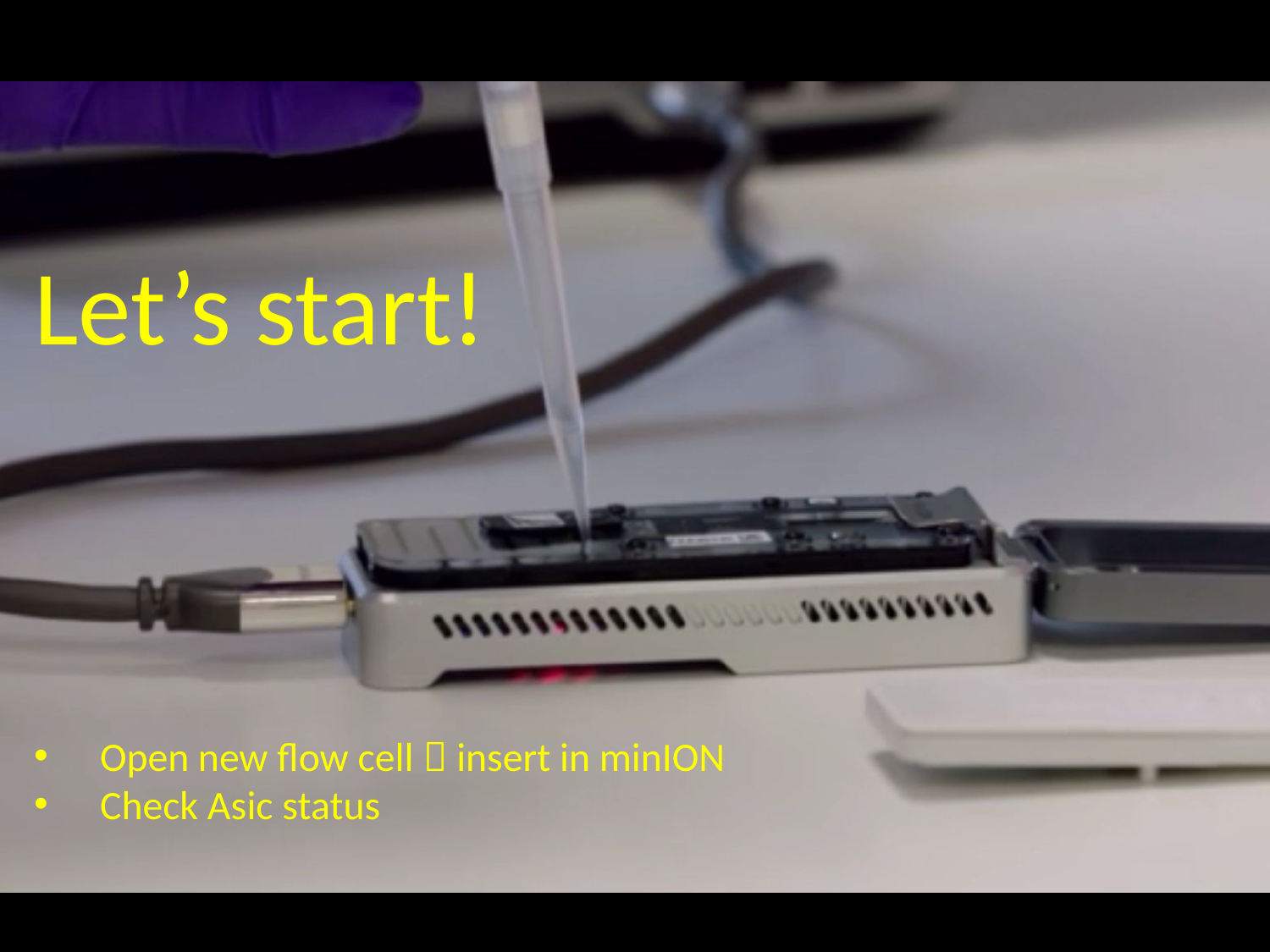

# Let’s start!
Open new flow cell  insert in minION
Check Asic status

## Slide 23
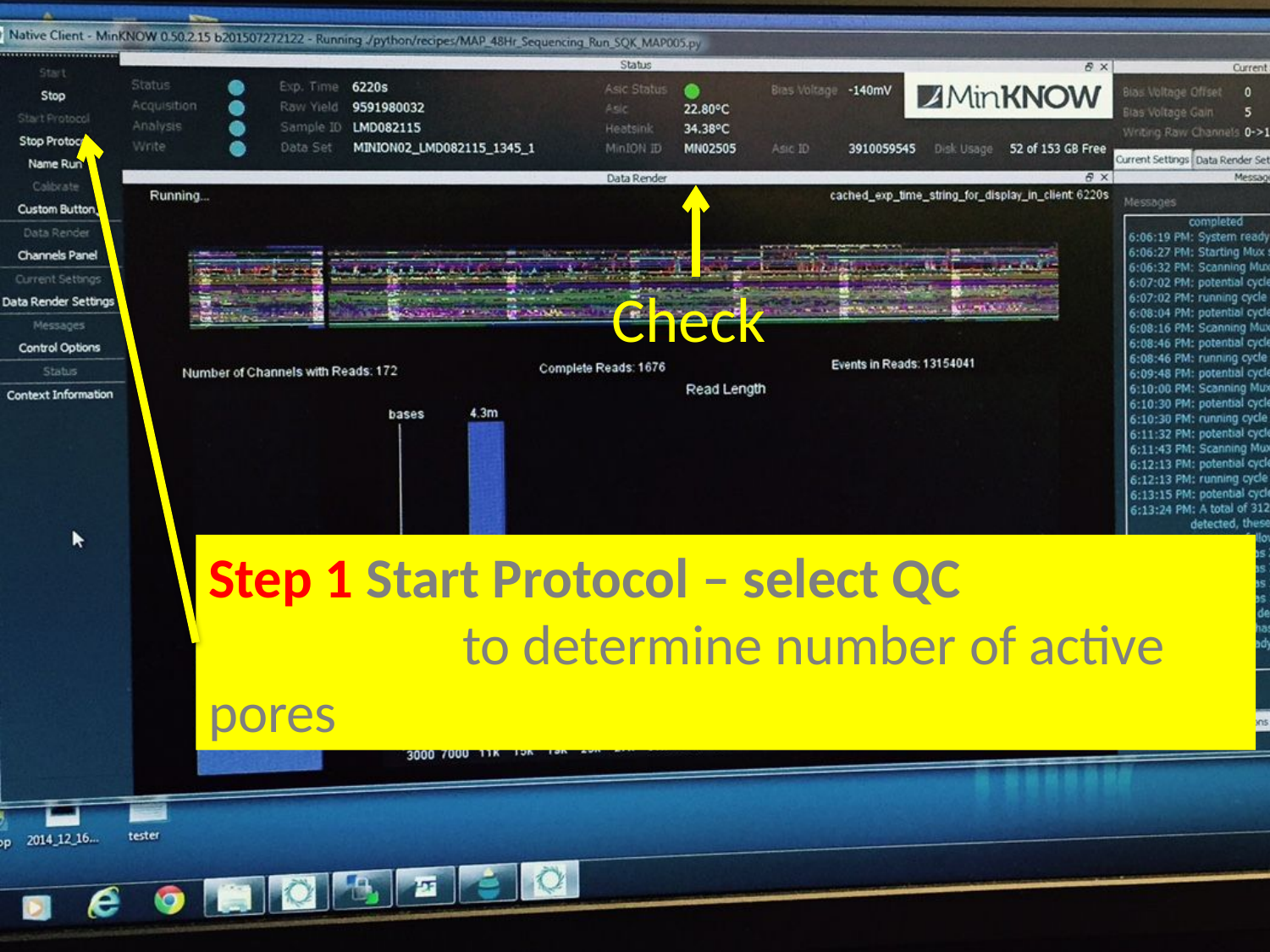

Check
Step 1 Start Protocol – select QC
		to determine number of active pores

## Slide 24
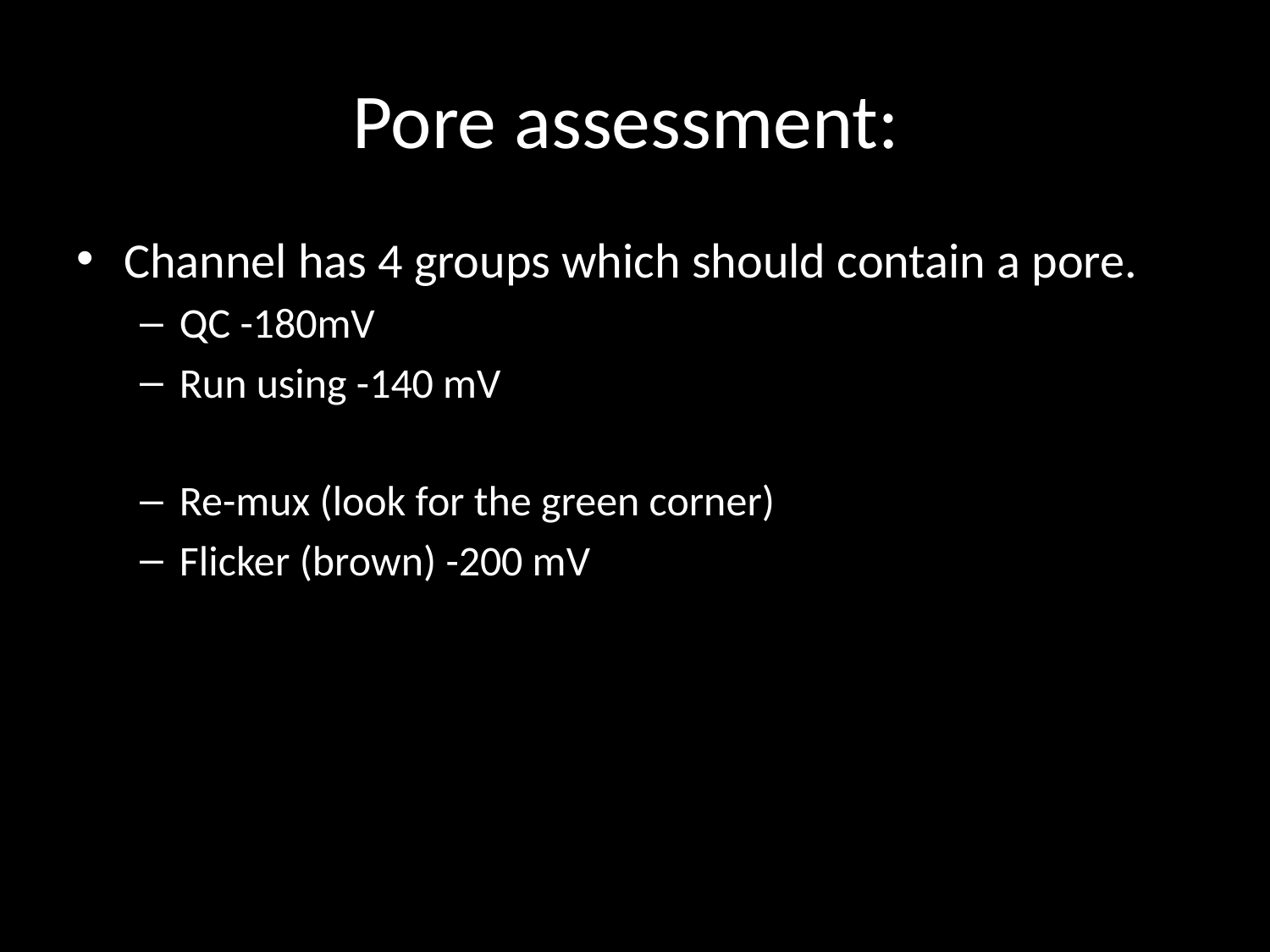

# Pore assessment:
Channel has 4 groups which should contain a pore.
QC -180mV
Run using -140 mV
Re-mux (look for the green corner)
Flicker (brown) -200 mV

## Slide 25
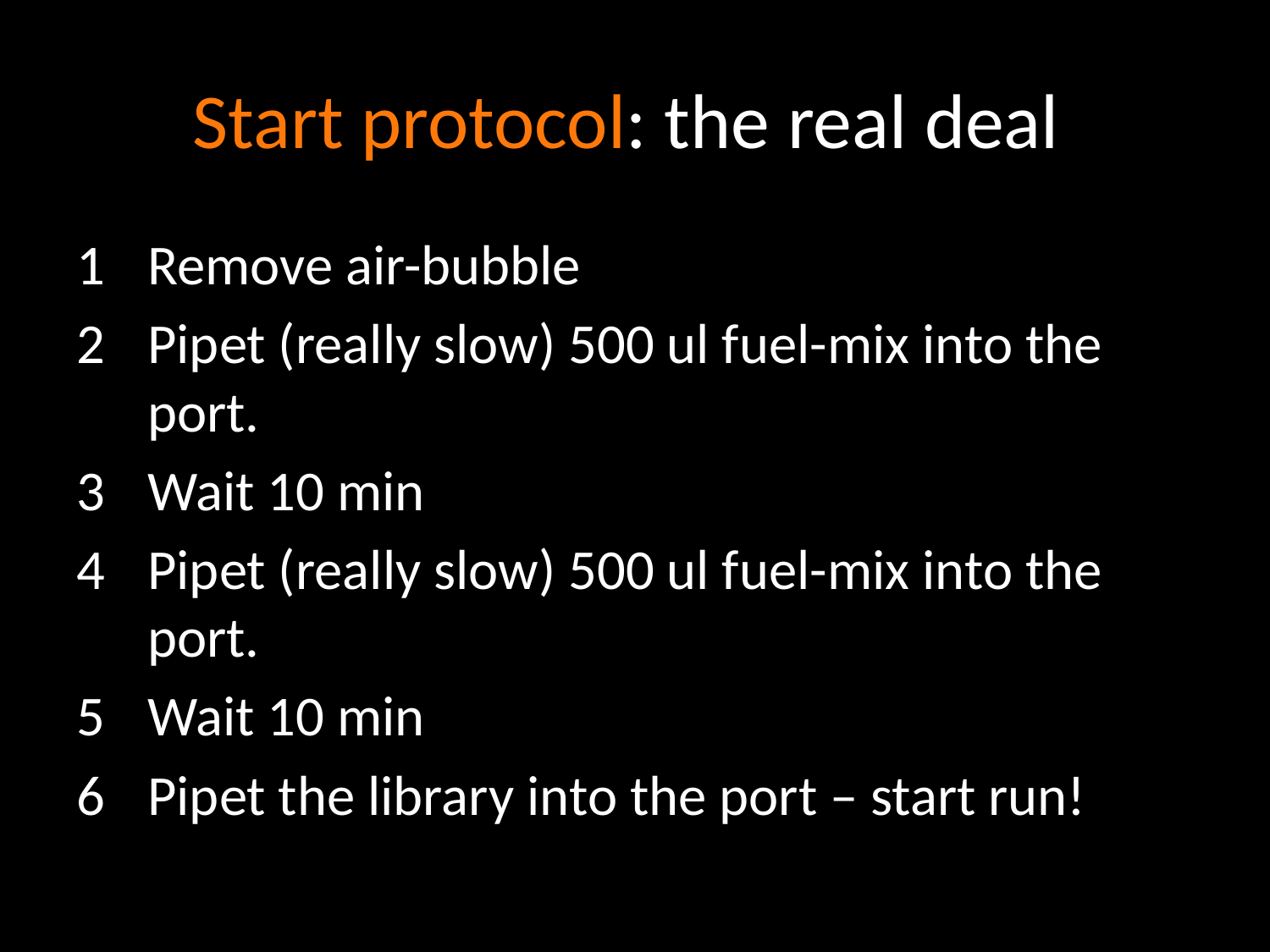

# Start protocol: the real deal
Remove air-bubble
Pipet (really slow) 500 ul fuel-mix into the port.
Wait 10 min
Pipet (really slow) 500 ul fuel-mix into the port.
Wait 10 min
Pipet the library into the port – start run!

## Slide 26
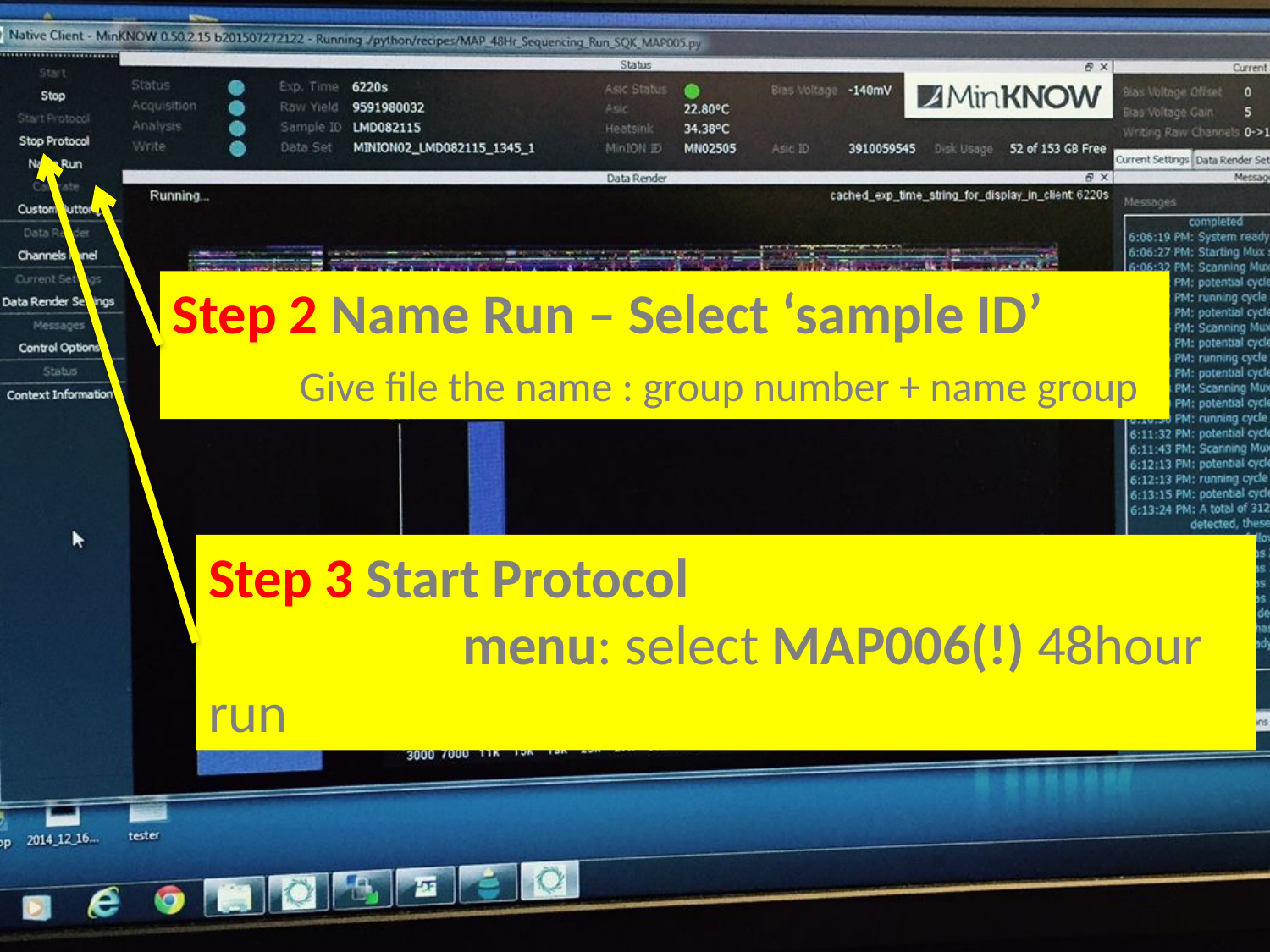

Step 2 Name Run – Select ‘sample ID’
	Give file the name : group number + name group
Step 3 Start Protocol
		menu: select MAP006(!) 48hour run

## Slide 27
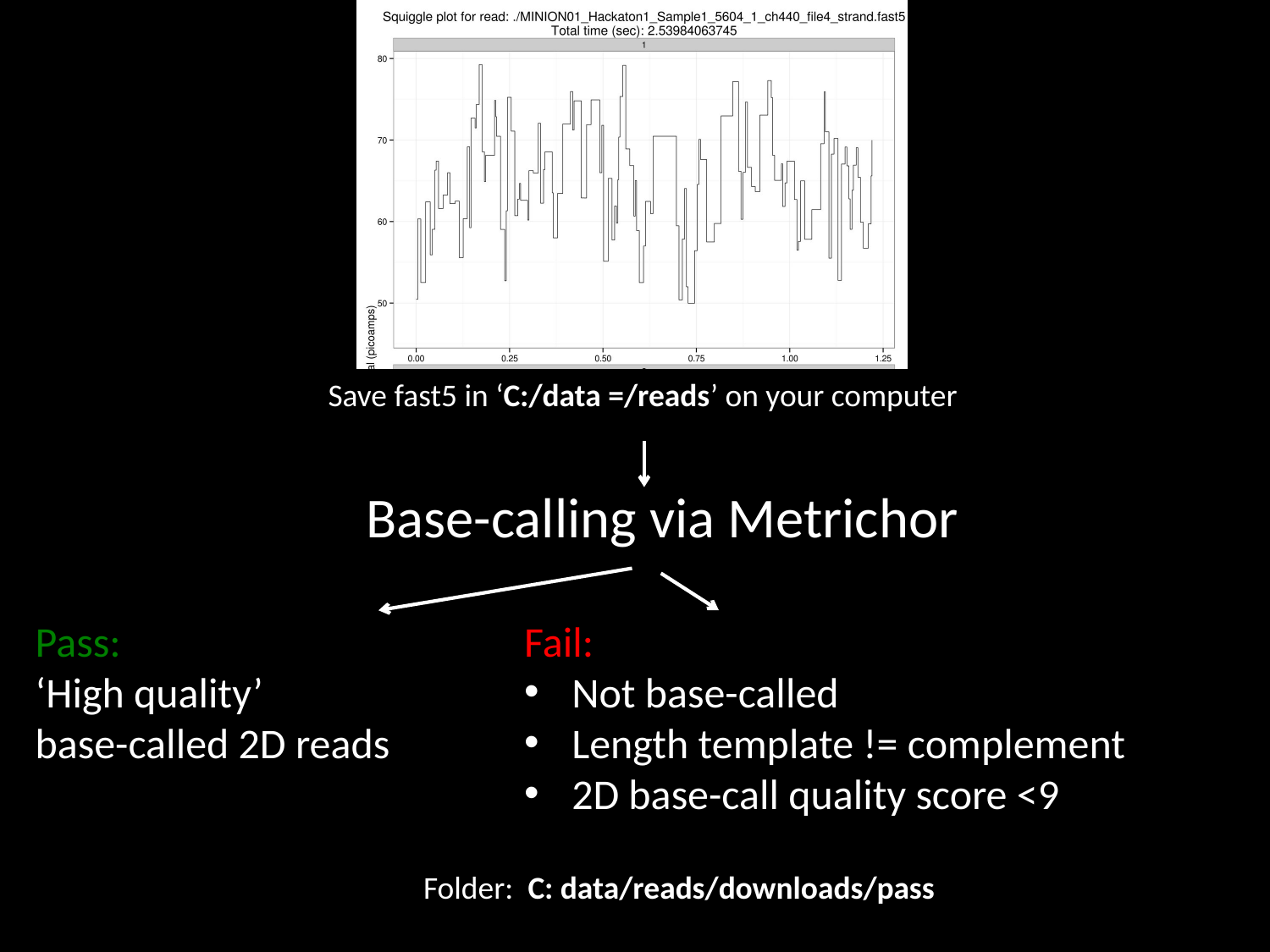

Save fast5 in ‘C:/data =/reads’ on your computer
Base-calling via Metrichor
Pass:
‘High quality’
base-called 2D reads
Fail:
Not base-called
Length template != complement
2D base-call quality score <9
Folder: C: data/reads/downloads/pass

## Slide 28
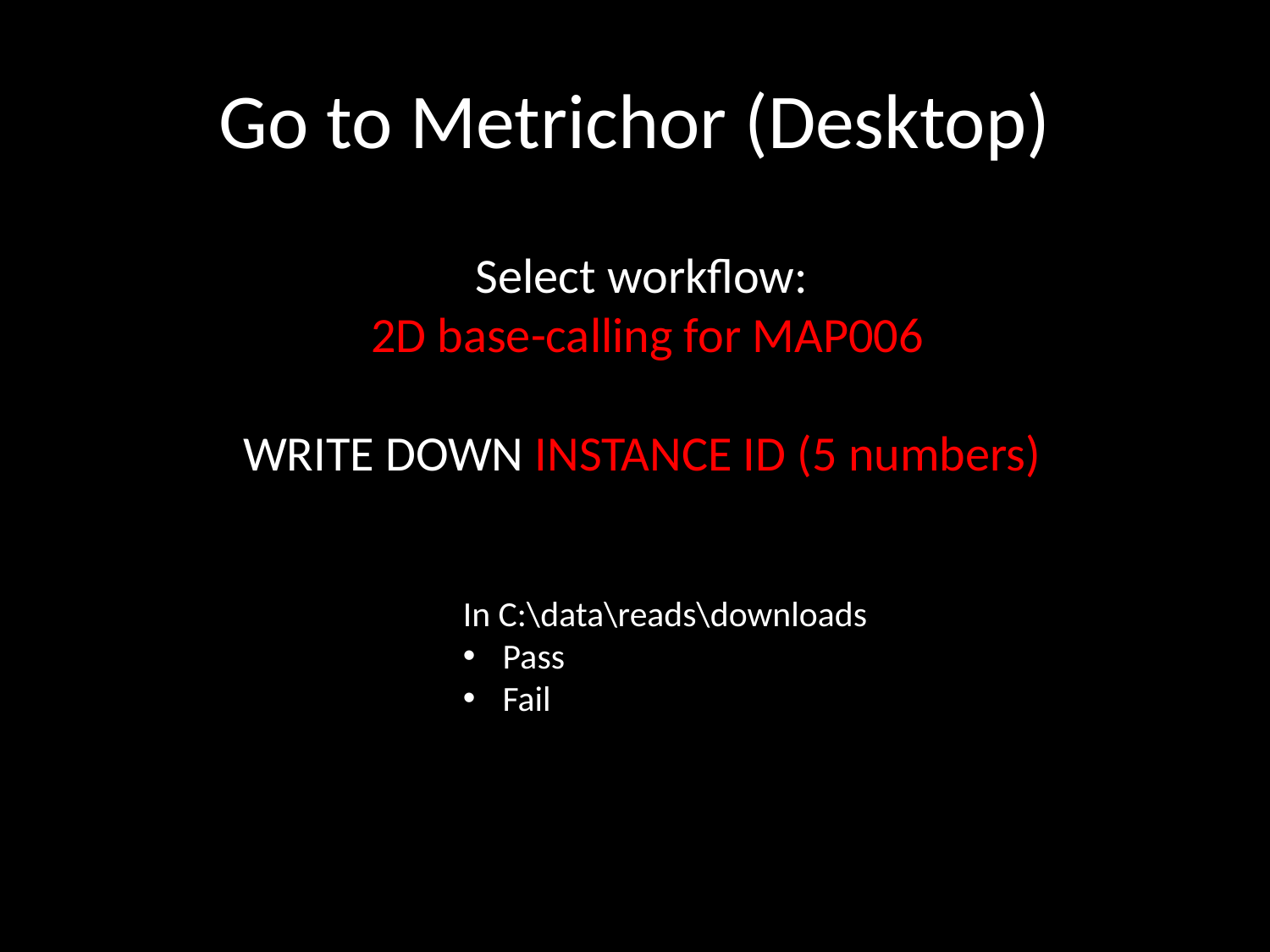

# Go to Metrichor (Desktop)
Select workflow:
2D base-calling for MAP006
WRITE DOWN INSTANCE ID (5 numbers)
In C:\data\reads\downloads
Pass
Fail

## Slide 29
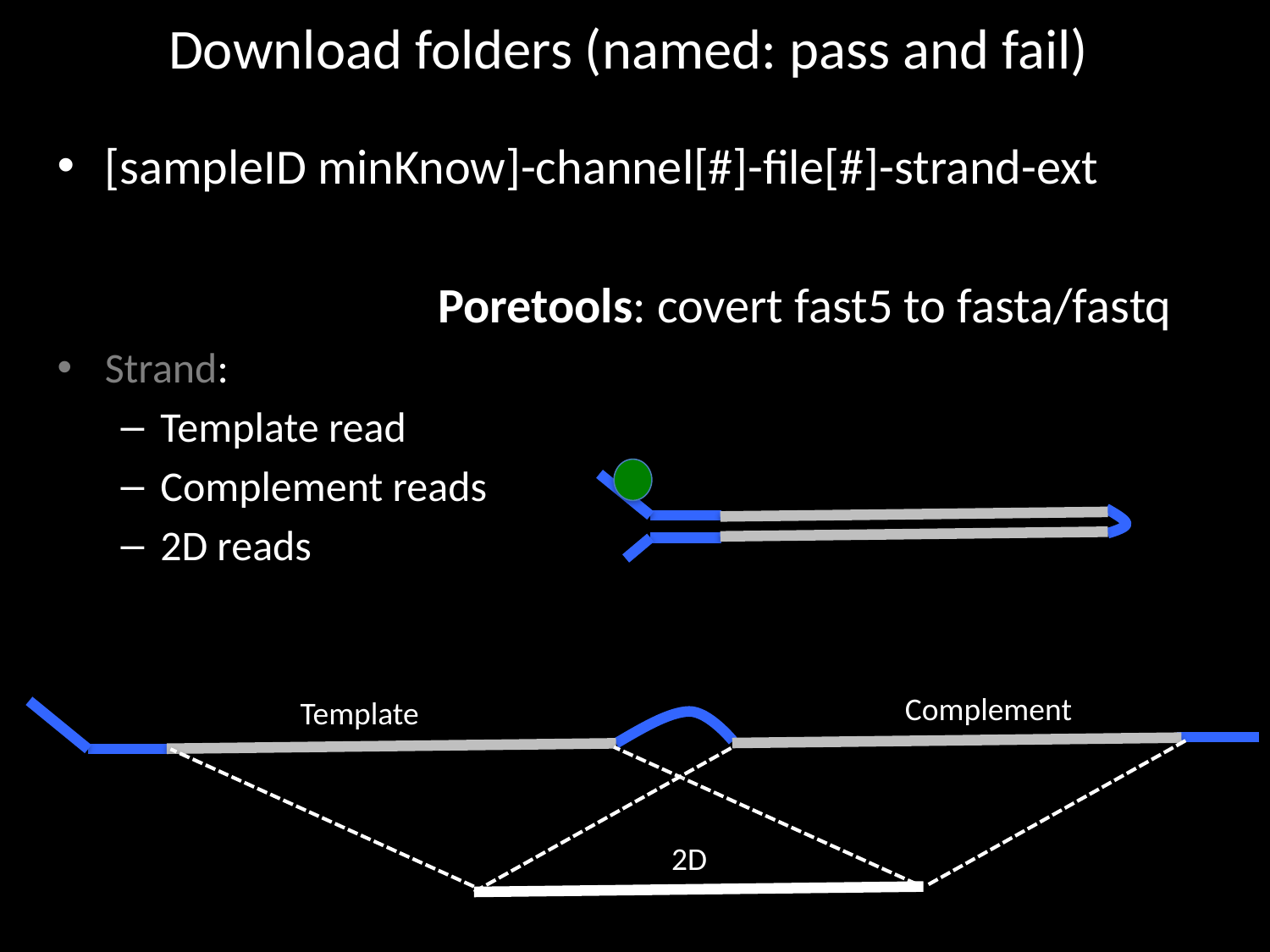

# Download folders (named: pass and fail)
[sampleID minKnow]-channel[#]-file[#]-strand-ext
			Poretools: covert fast5 to fasta/fastq
Strand:
Template read
Complement reads
2D reads
Complement
Template
2D

## Slide 30
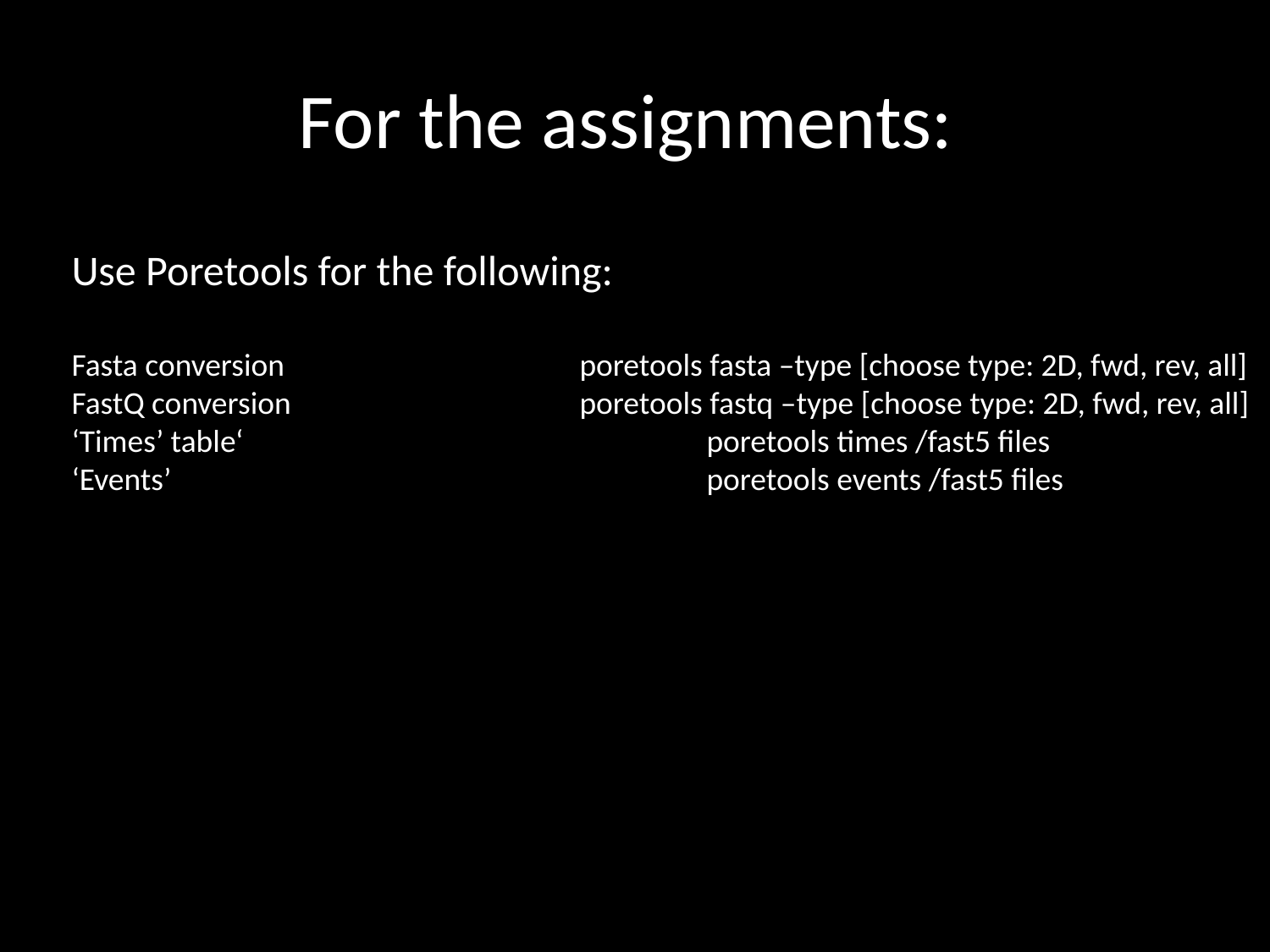

# For the assignments:
Use Poretools for the following:
Fasta conversion 			poretools fasta –type [choose type: 2D, fwd, rev, all]
FastQ conversion 			poretools fastq –type [choose type: 2D, fwd, rev, all]
‘Times’ table‘ 				poretools times /fast5 files
‘Events’ 					poretools events /fast5 files
