# Supplementary material for: Using mobile sequencers in an academic classroom
Source: eLife. 2016 Apr 7;5:e14258. doi: 10.7554/eLife.14258 (PMC4869913; doi:10.7554/eLife.14258)
Supplement: Supplementary file 7. — DOI: http://dx.doi.org/10.7554/eLife.14258.011 [file elife-14258-supp7.pptx]

## Slide 1
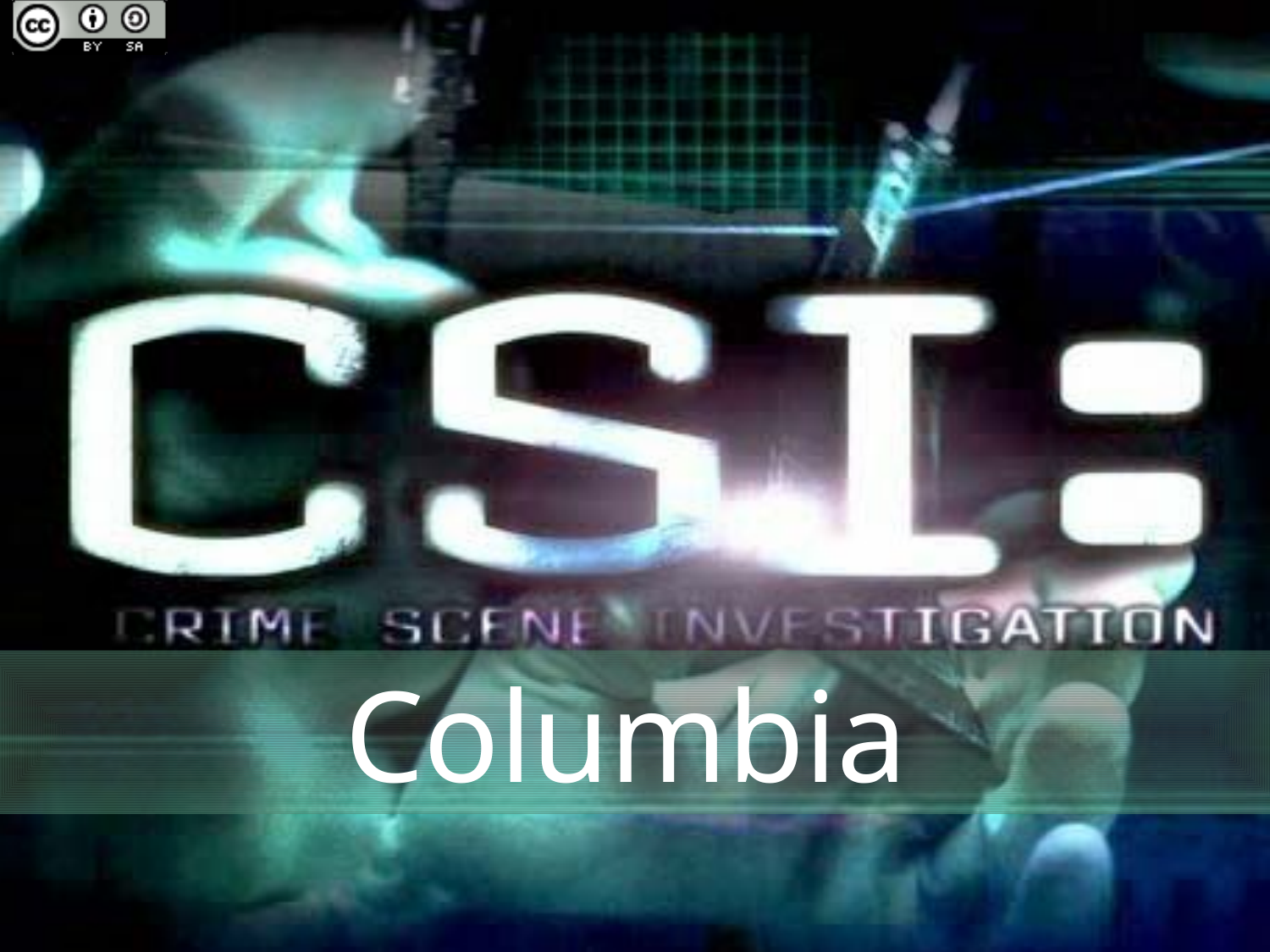

# Columbia

## Slide 2
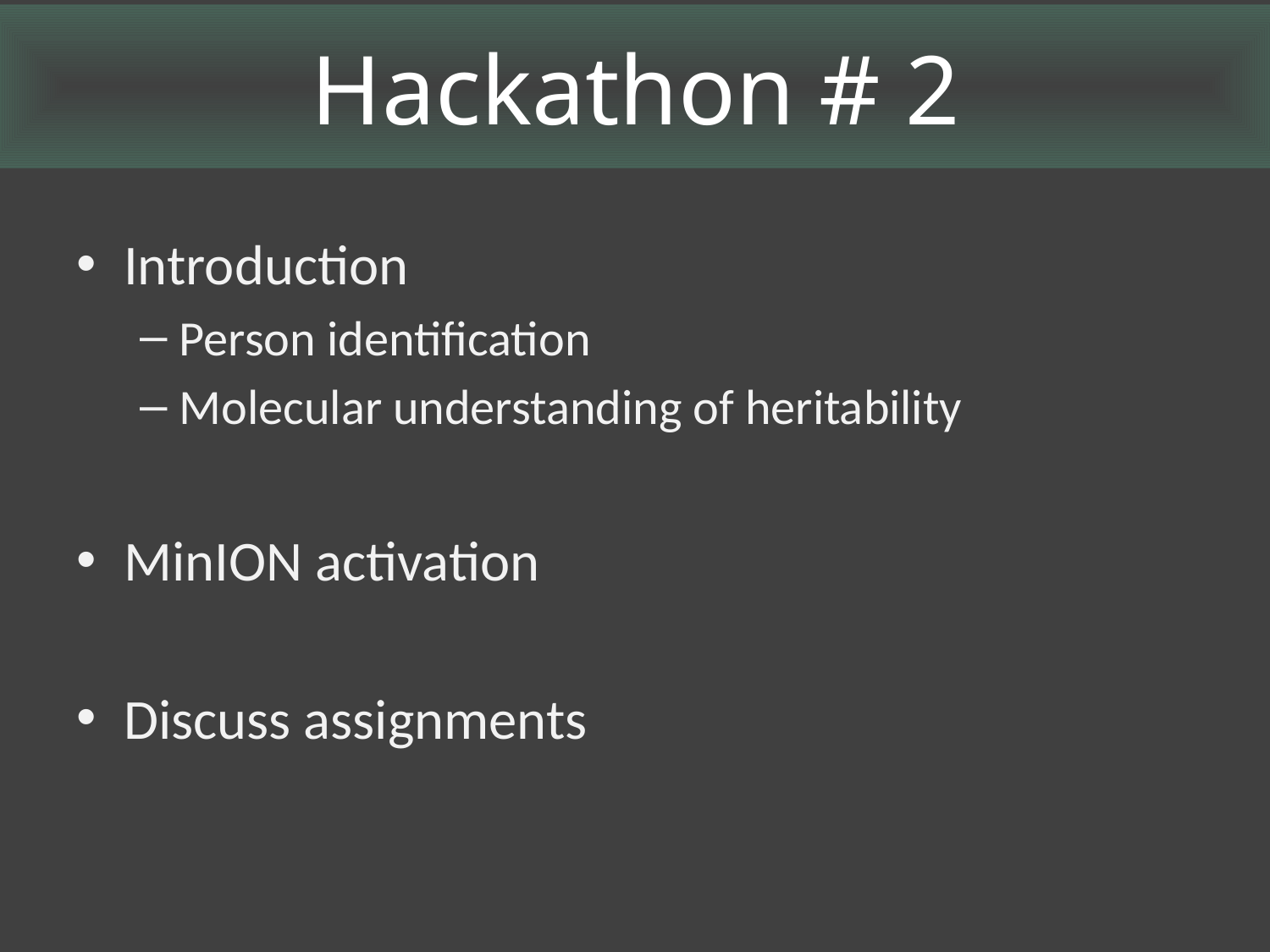

Hackathon # 2
Introduction
Person identification
Molecular understanding of heritability
MinION activation
Discuss assignments

## Slide 3
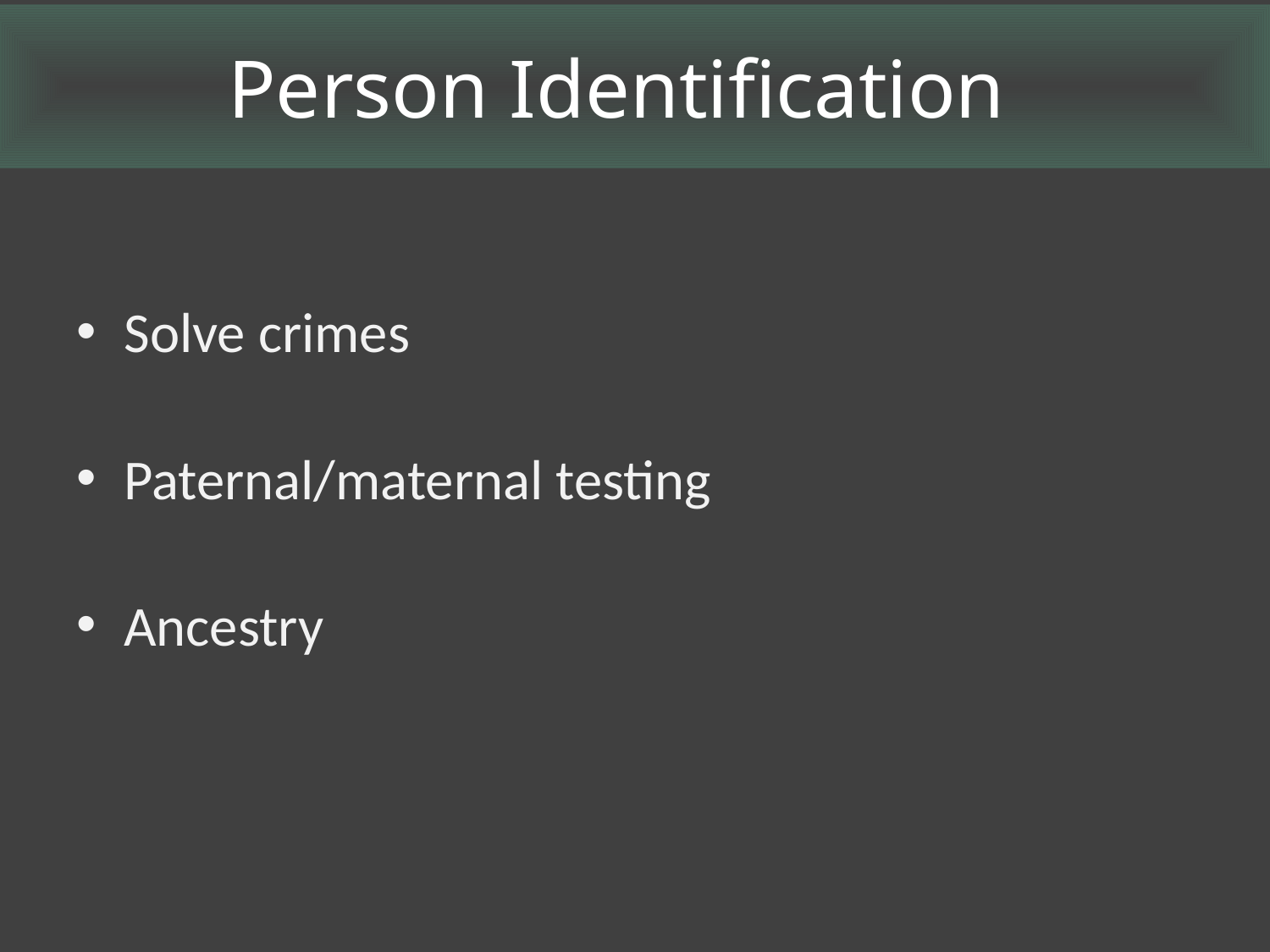

Person Identification
Solve crimes
Paternal/maternal testing
Ancestry

## Slide 4
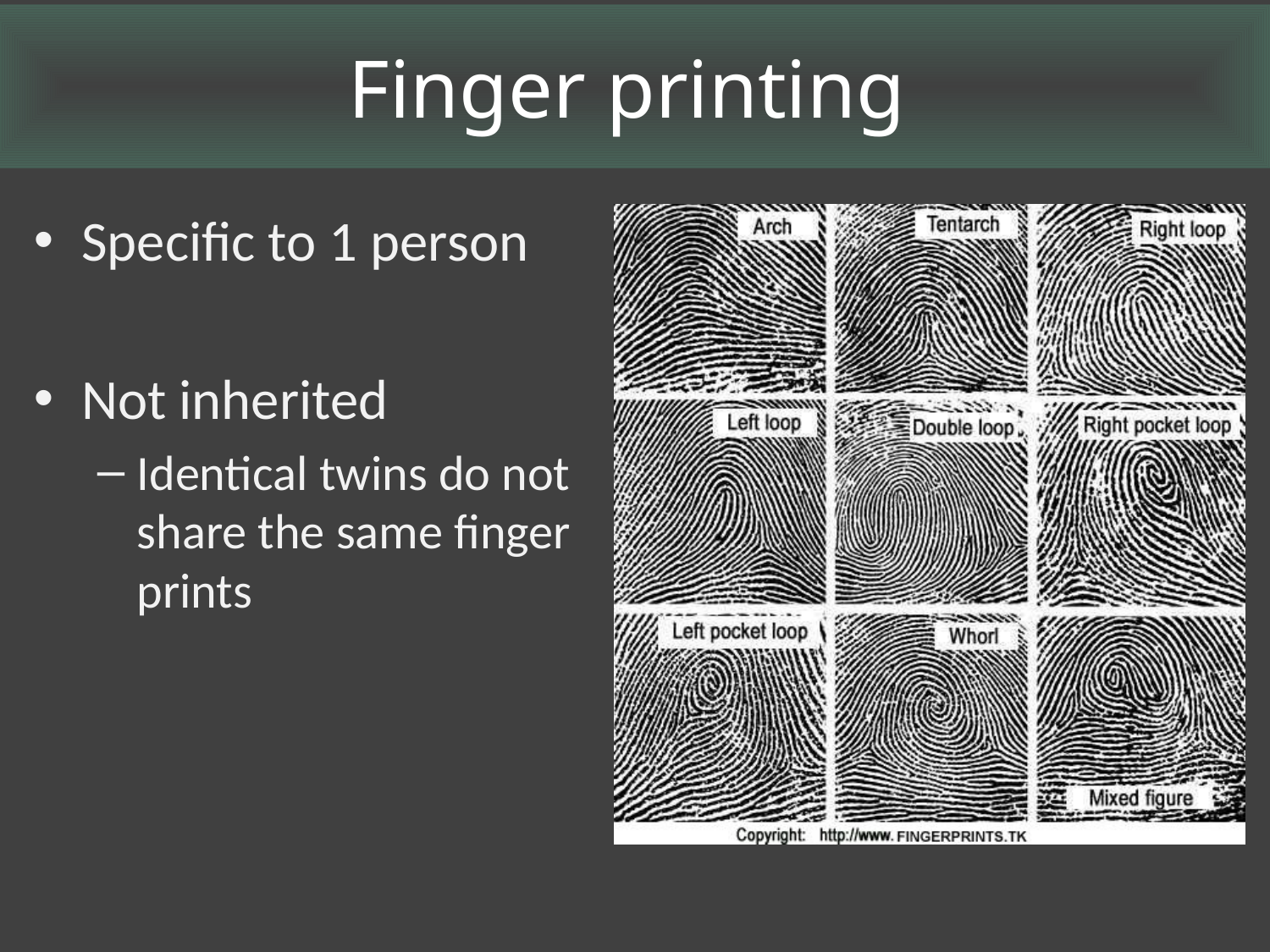

Finger printing
Specific to 1 person
Not inherited
Identical twins do not share the same finger prints

## Slide 5
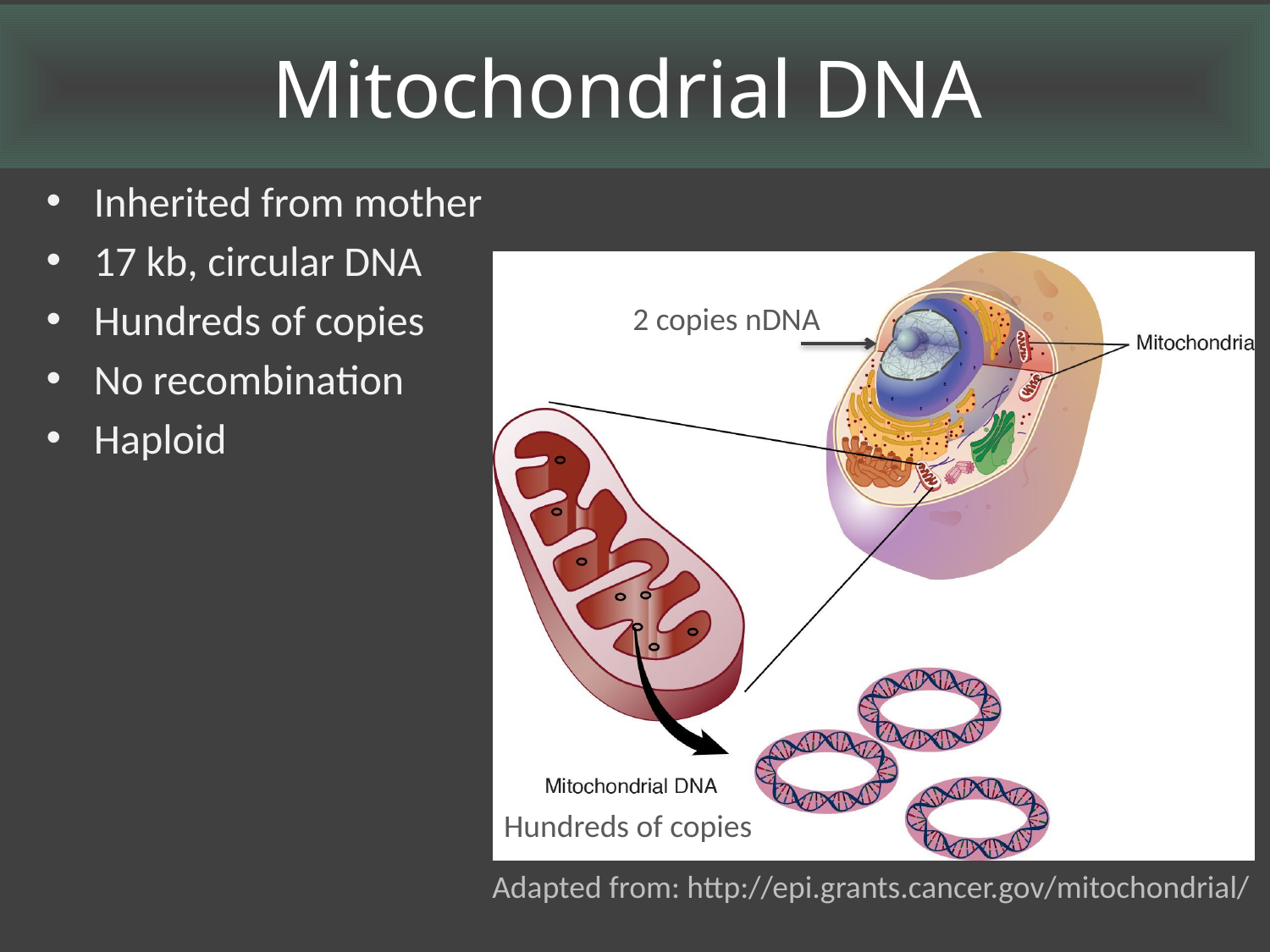

Mitochondrial DNA
Inherited from mother
17 kb, circular DNA
Hundreds of copies
No recombination
Haploid
2 copies nDNA
Hundreds of copies
Adapted from: http://epi.grants.cancer.gov/mitochondrial/

## Slide 6
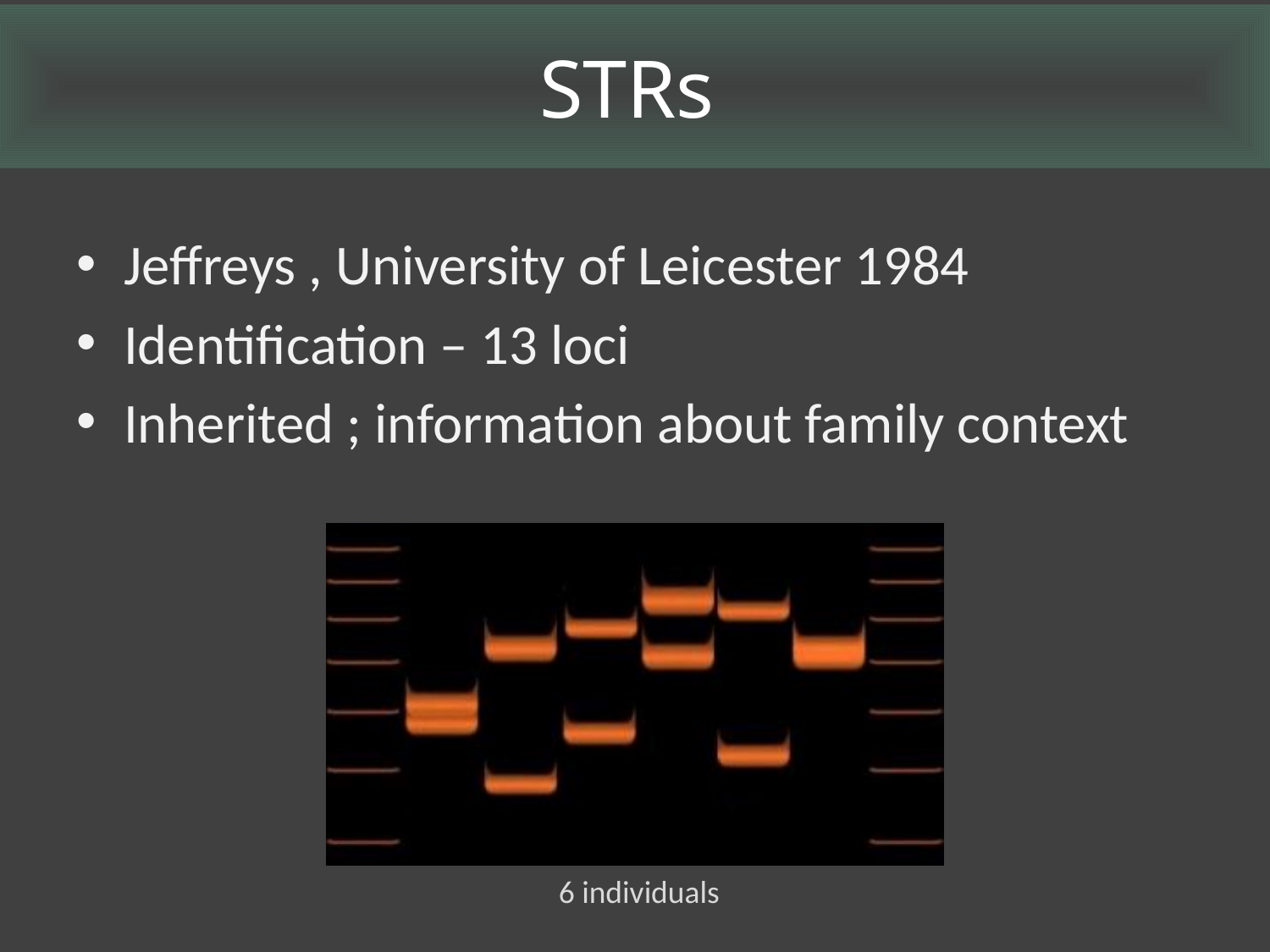

STRs
Jeffreys , University of Leicester 1984
Identification – 13 loci
Inherited ; information about family context
6 individuals

## Slide 7
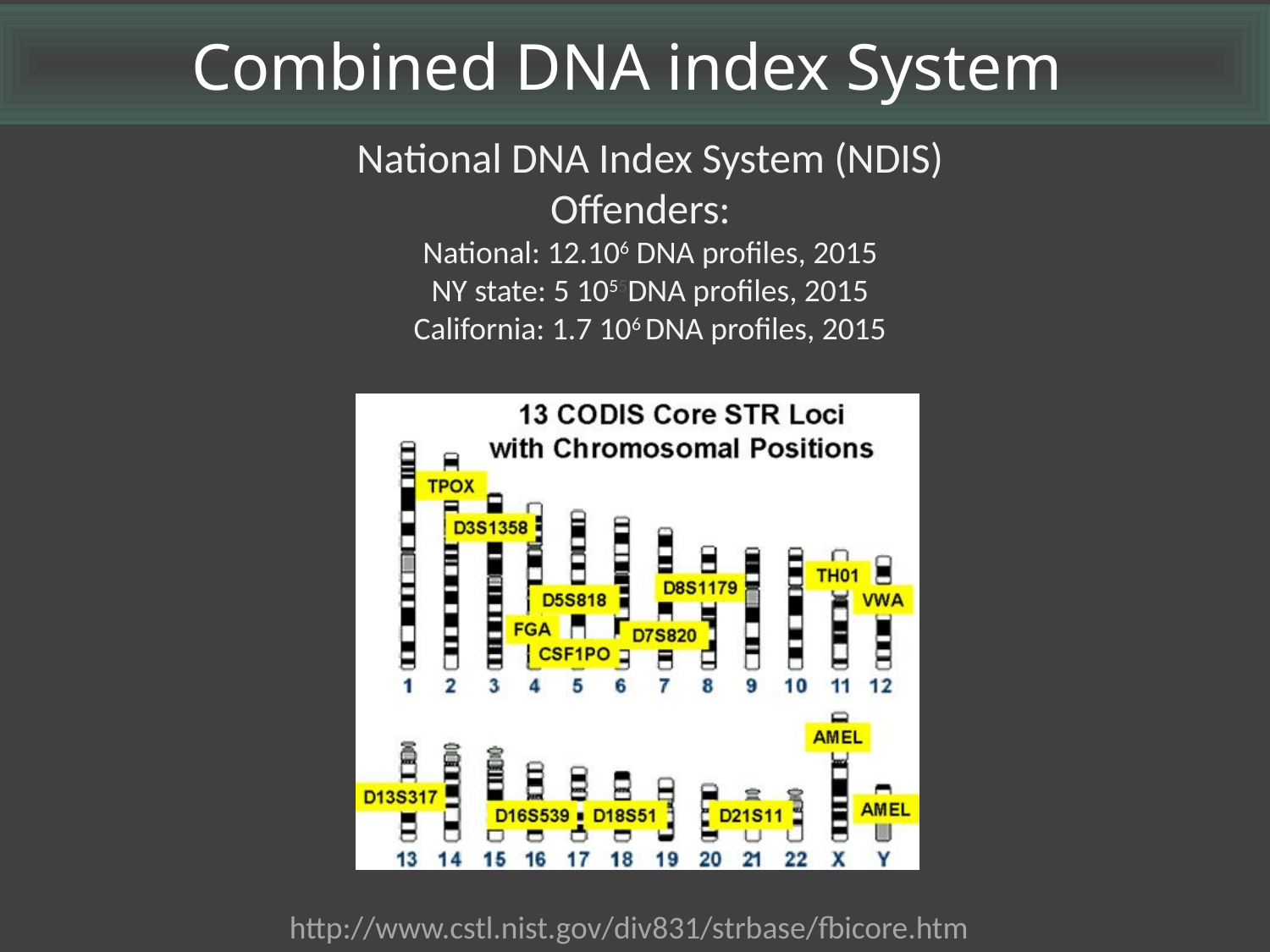

Combined DNA index System
National DNA Index System (NDIS)
Offenders:
National: 12.106 DNA profiles, 2015
NY state: 5 1055DNA profiles, 2015
California: 1.7 106 DNA profiles, 2015
http://www.cstl.nist.gov/div831/strbase/fbicore.htm

## Slide 8
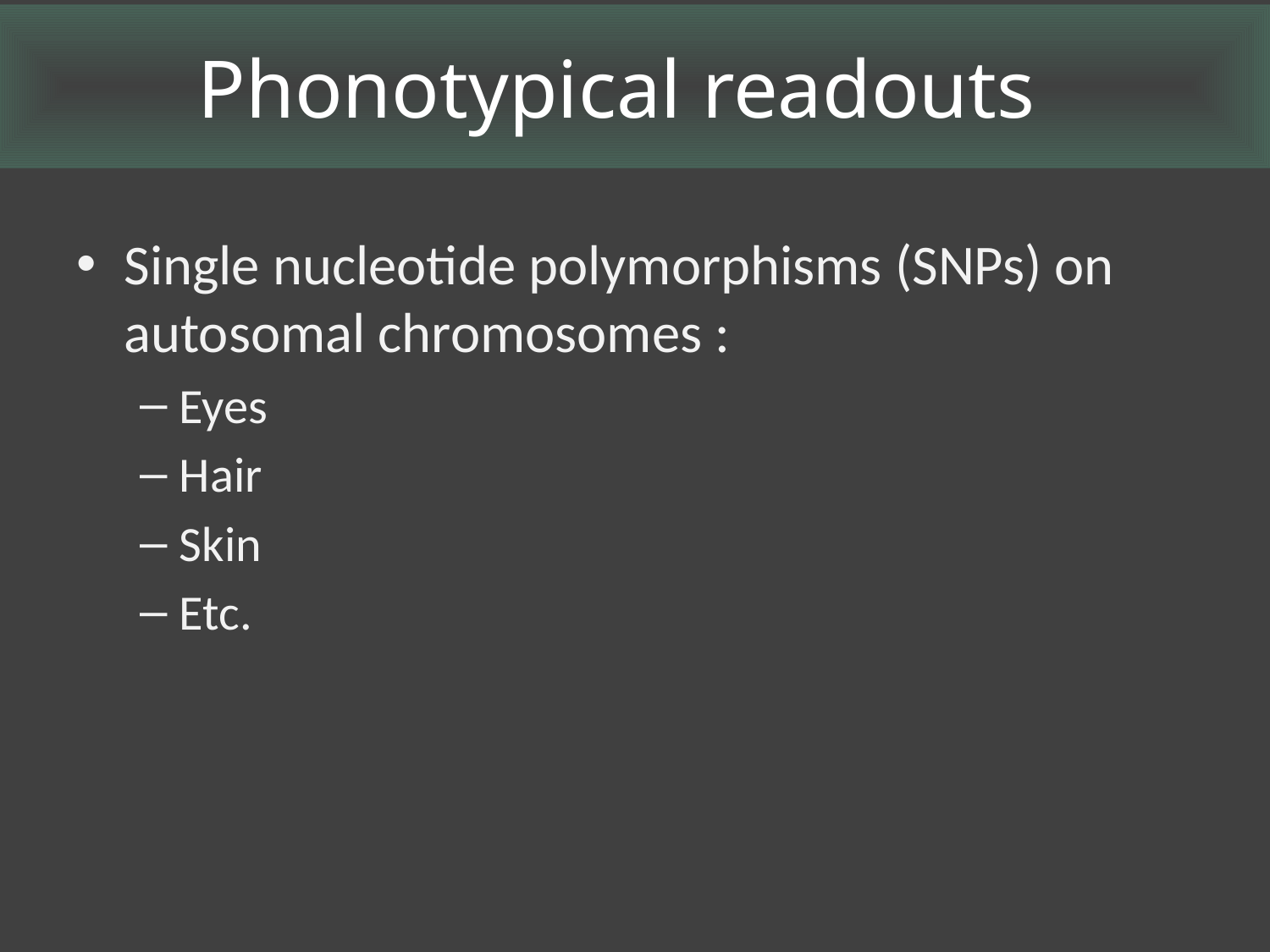

Phonotypical readouts
Single nucleotide polymorphisms (SNPs) on autosomal chromosomes :
Eyes
Hair
Skin
Etc.

## Slide 9
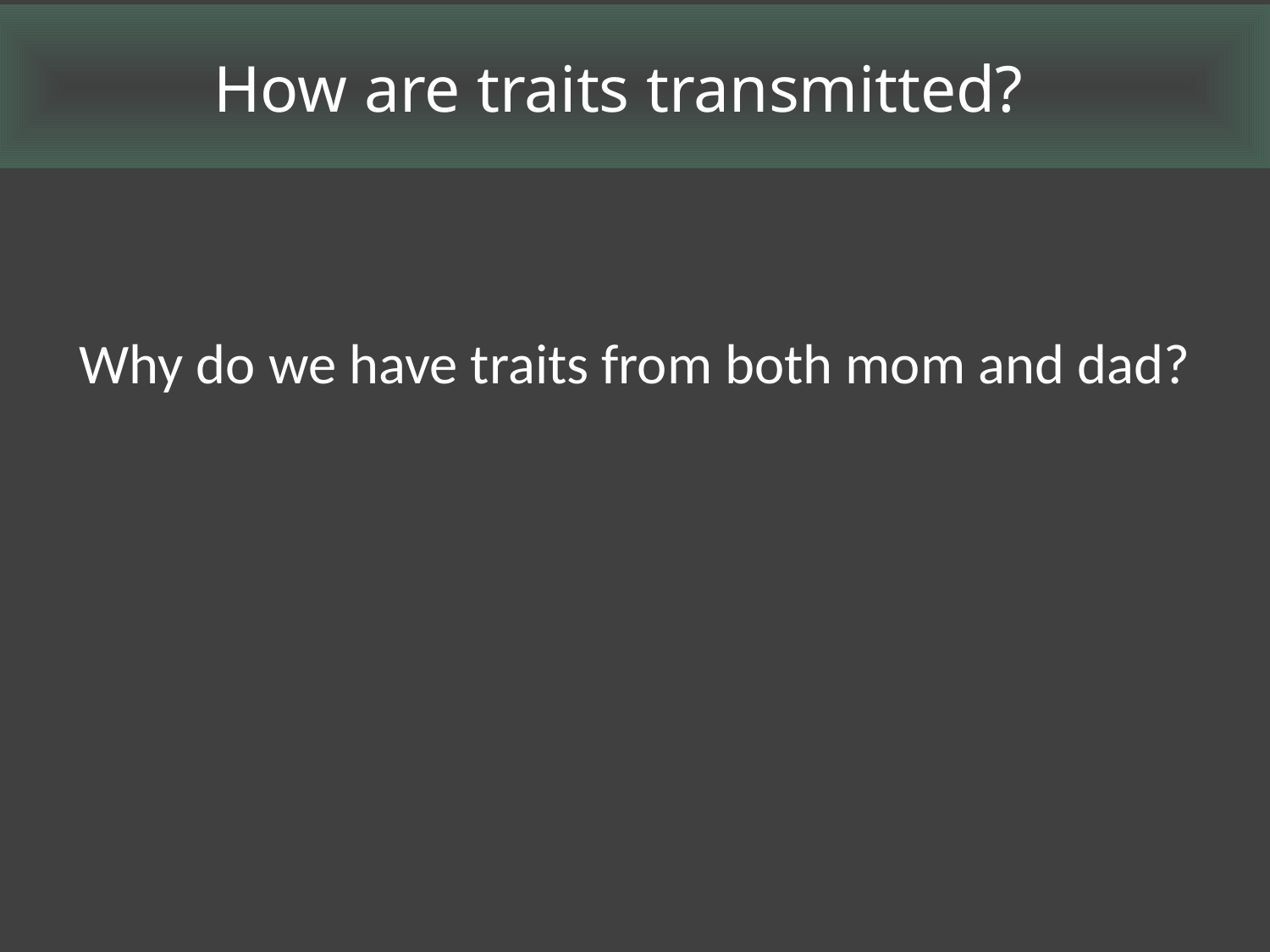

How are traits transmitted?
Why do we have traits from both mom and dad?

## Slide 10
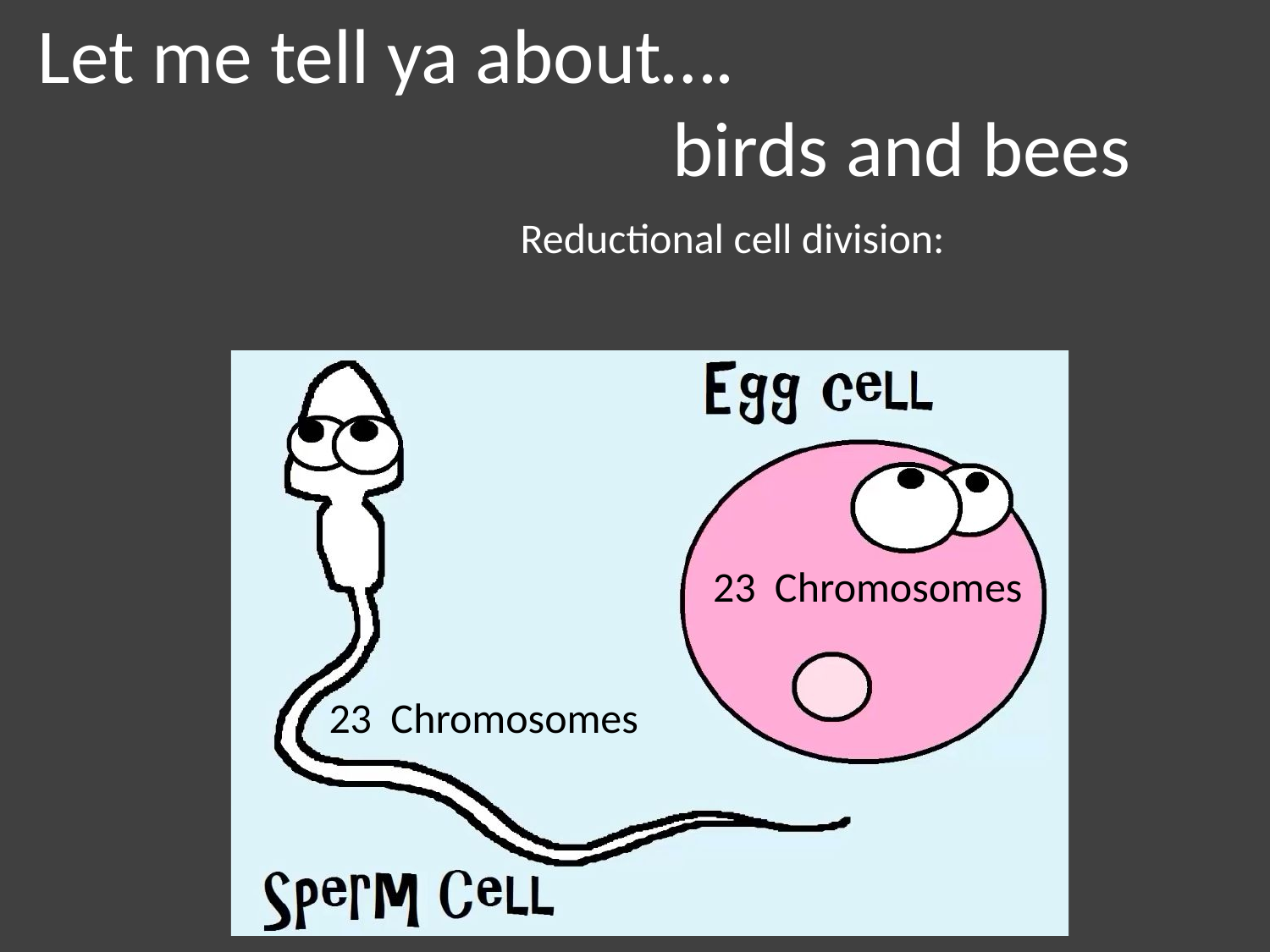

Let me tell ya about….
					birds and bees
Reductional cell division:
23 Chromosomes
23 Chromosomes

## Slide 11
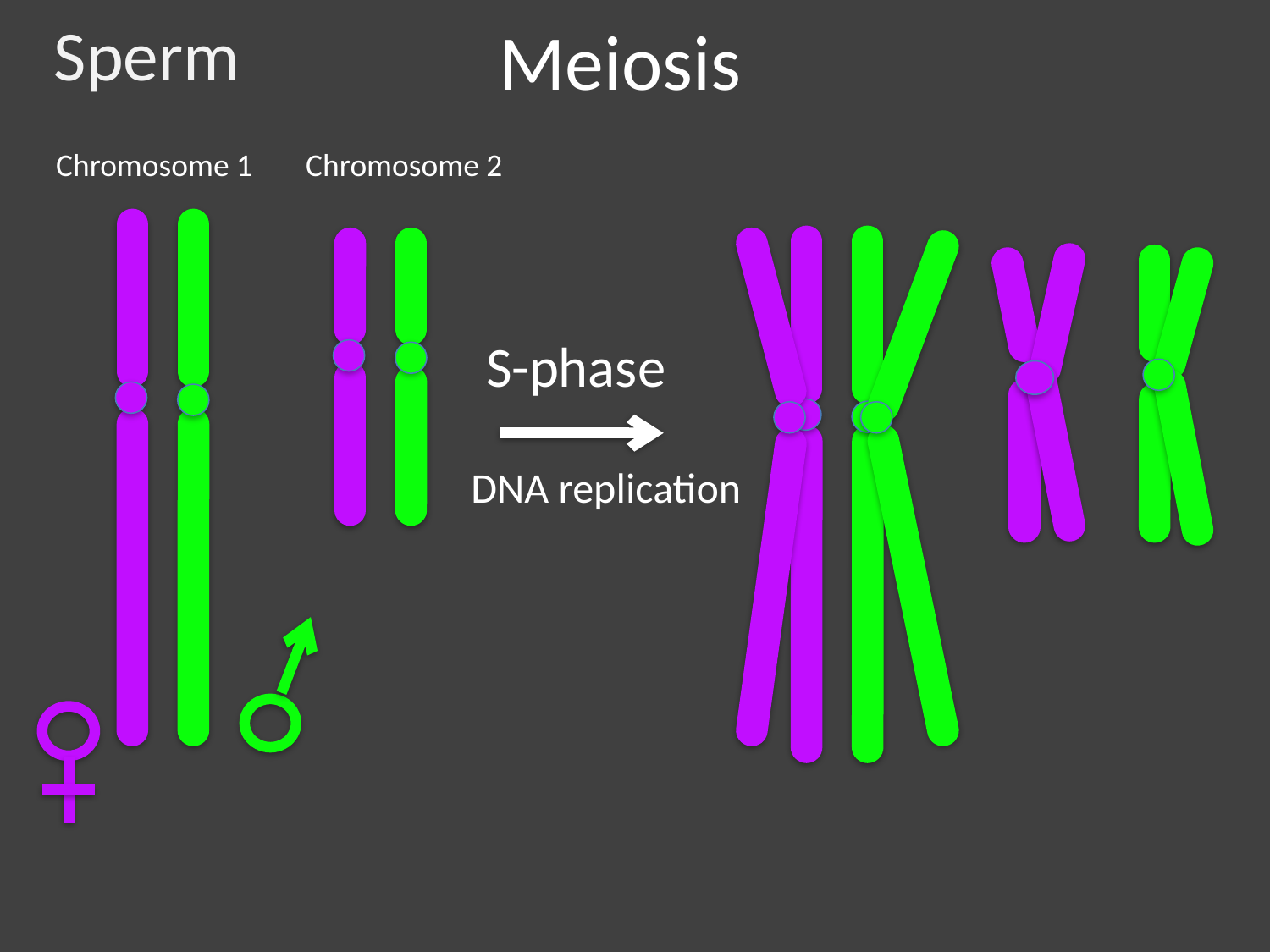

# Meiosis
Sperm
Chromosome 1
Chromosome 2
S-phase
DNA replication

## Slide 12
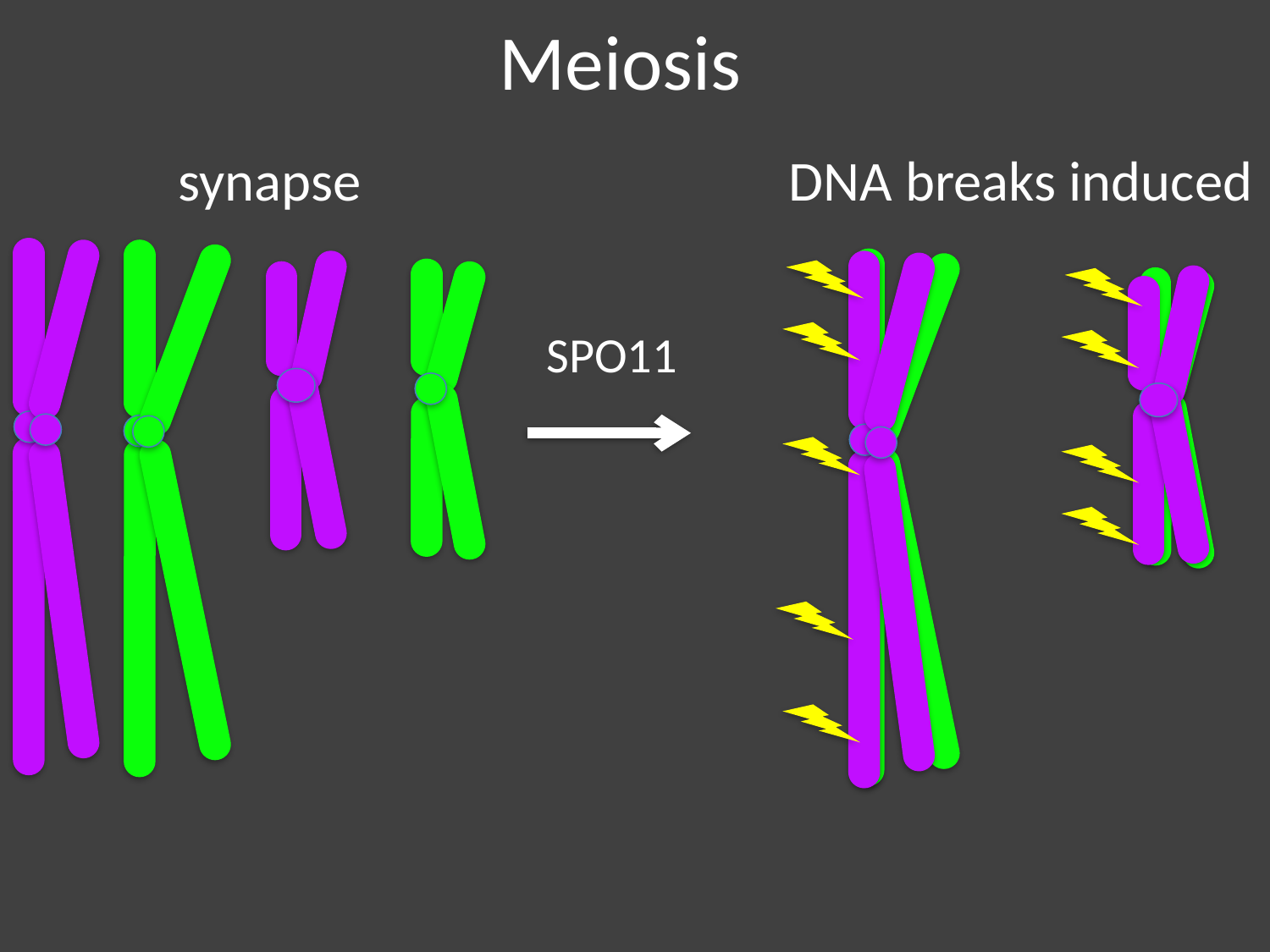

# Meiosis
synapse
DNA breaks induced
SPO11

## Slide 13
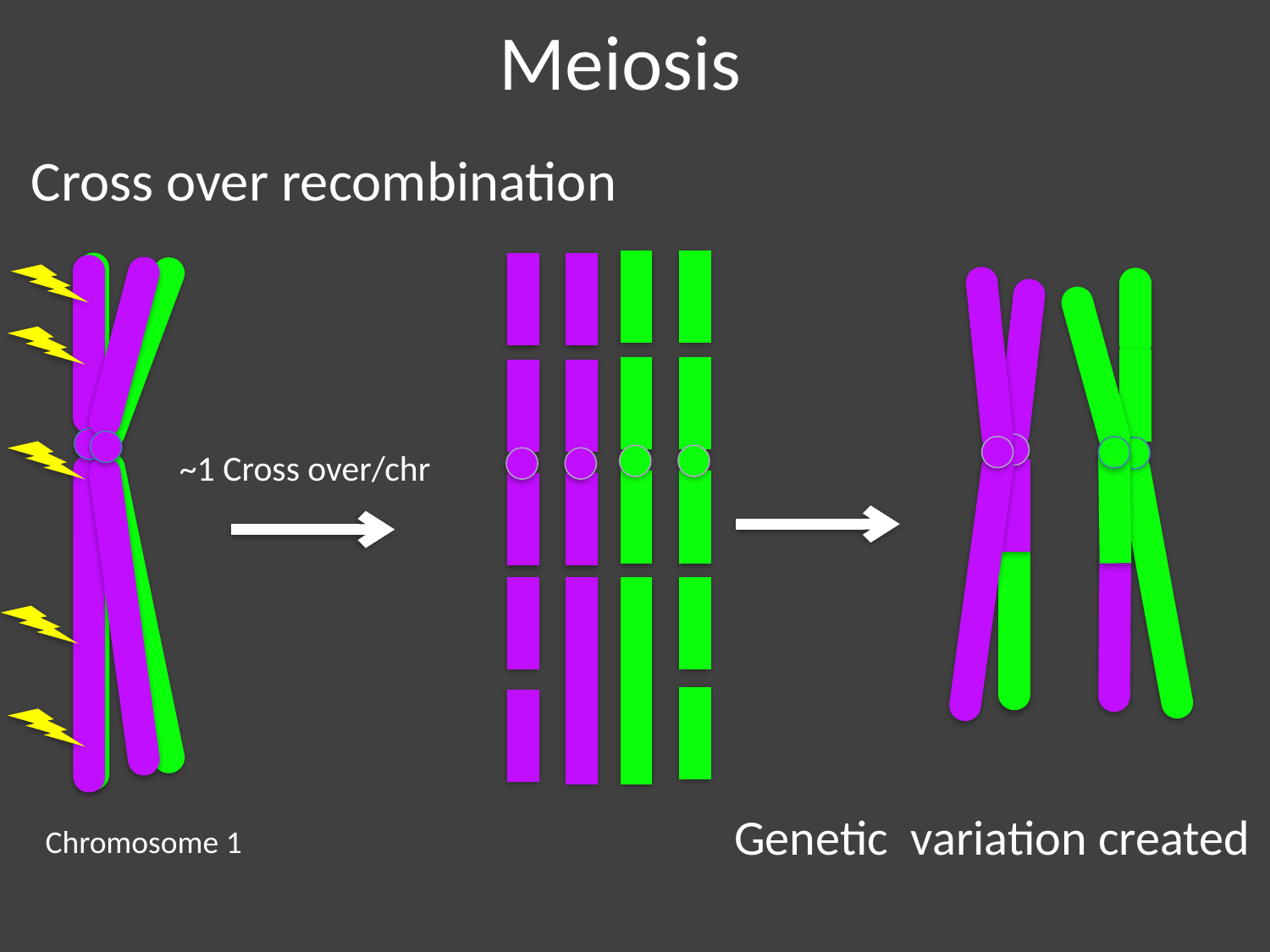

# Meiosis
Cross over recombination
~1 Cross over/chr
Genetic variation created
Chromosome 1

## Slide 14
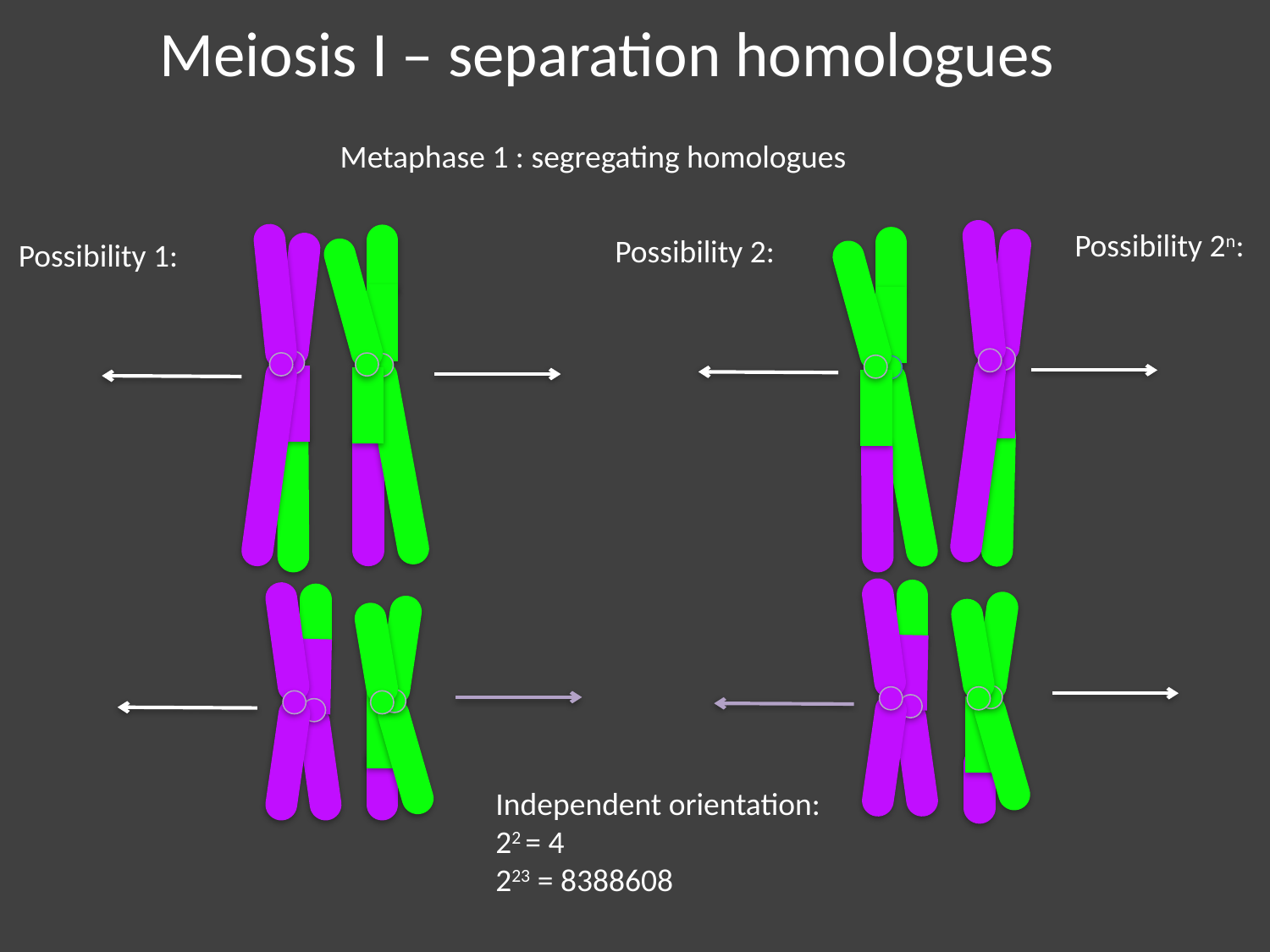

# Meiosis I – separation homologues
Metaphase 1 : segregating homologues
Possibility 2n:
Possibility 2:
Possibility 1:
Independent orientation:
22 = 4
223 = 8388608

## Slide 15
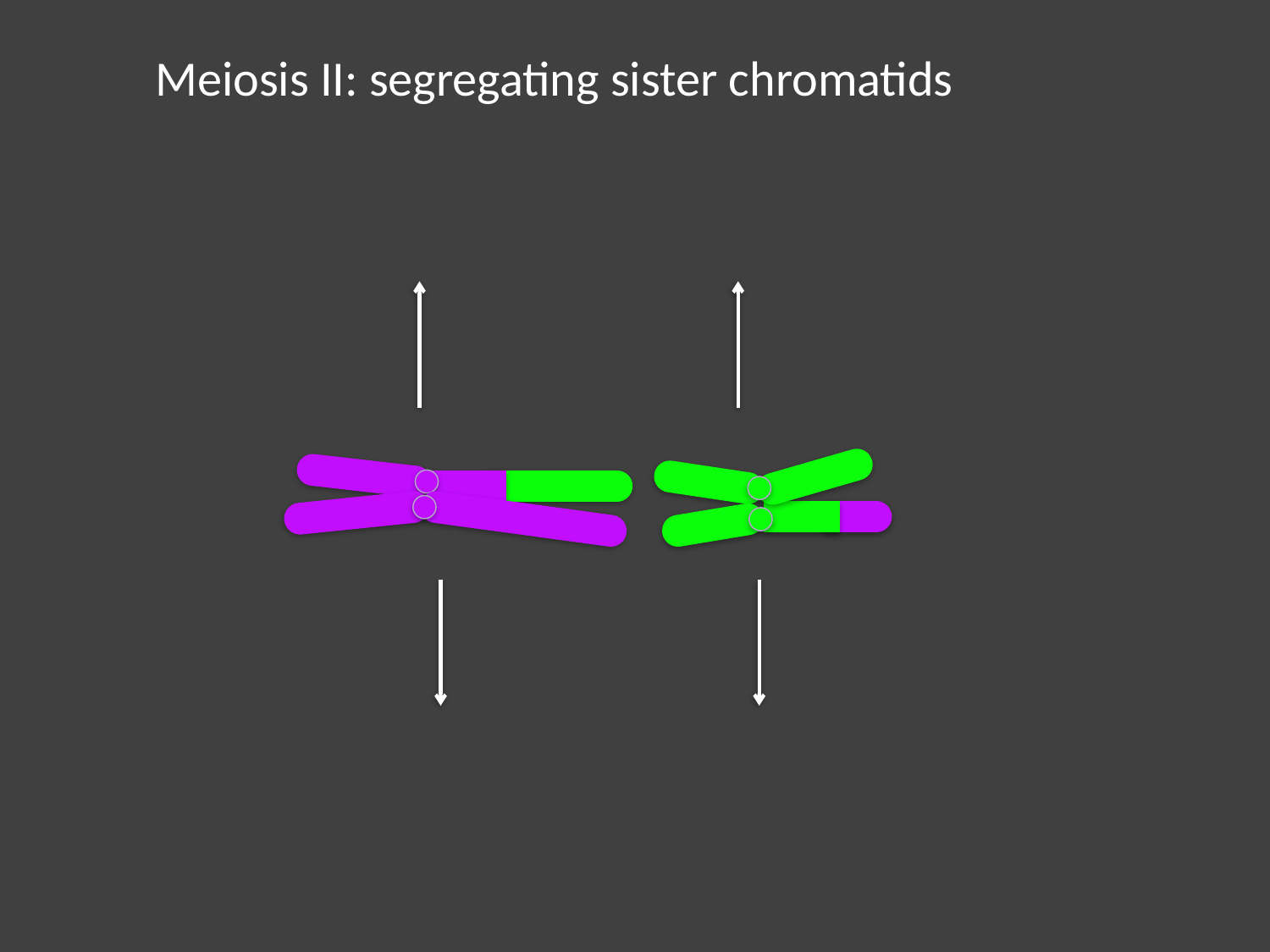

Meiosis II: segregating sister chromatids

## Slide 16
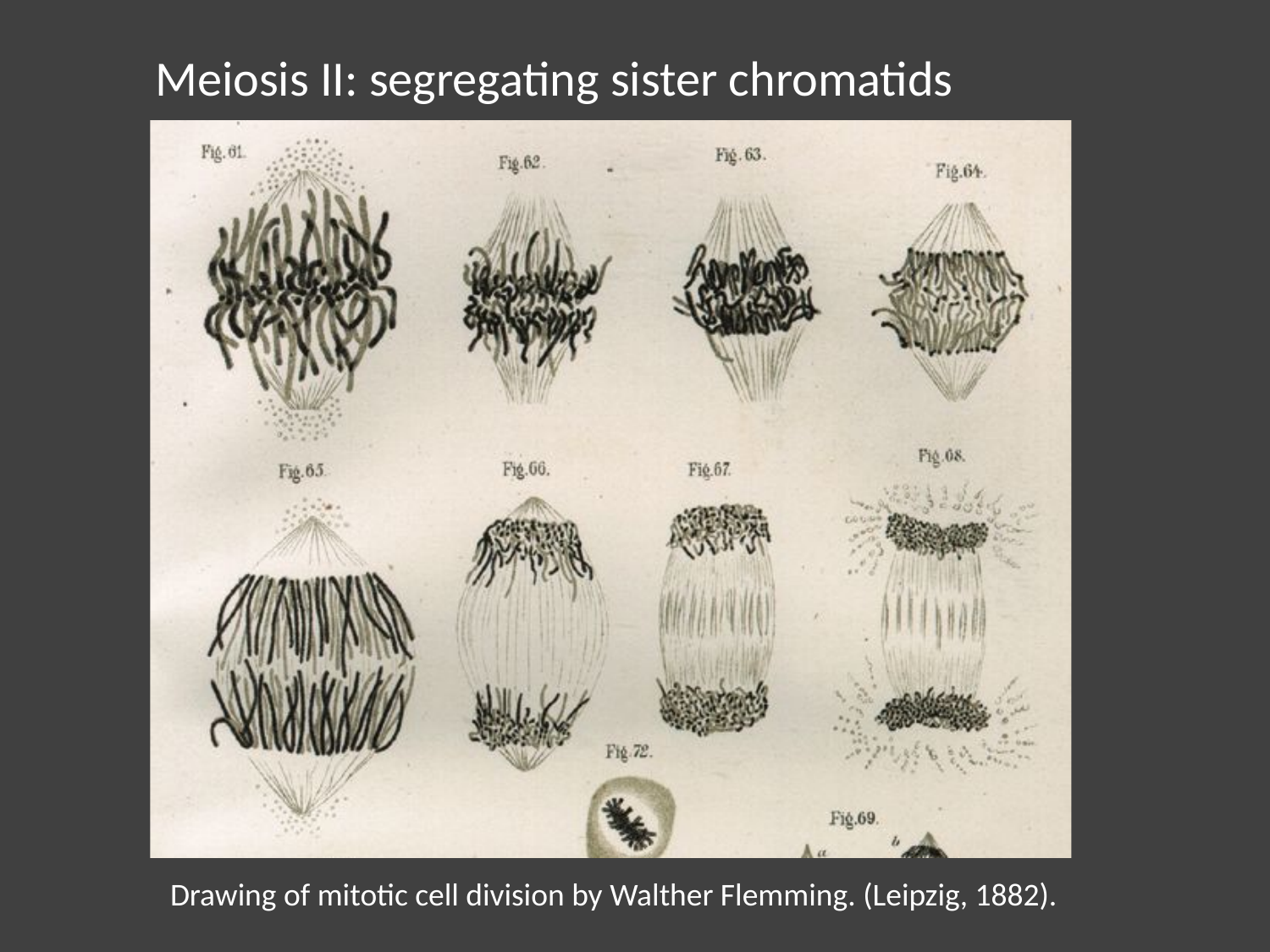

Meiosis II: segregating sister chromatids
 Drawing of mitotic cell division by Walther Flemming. (Leipzig, 1882).

## Slide 17
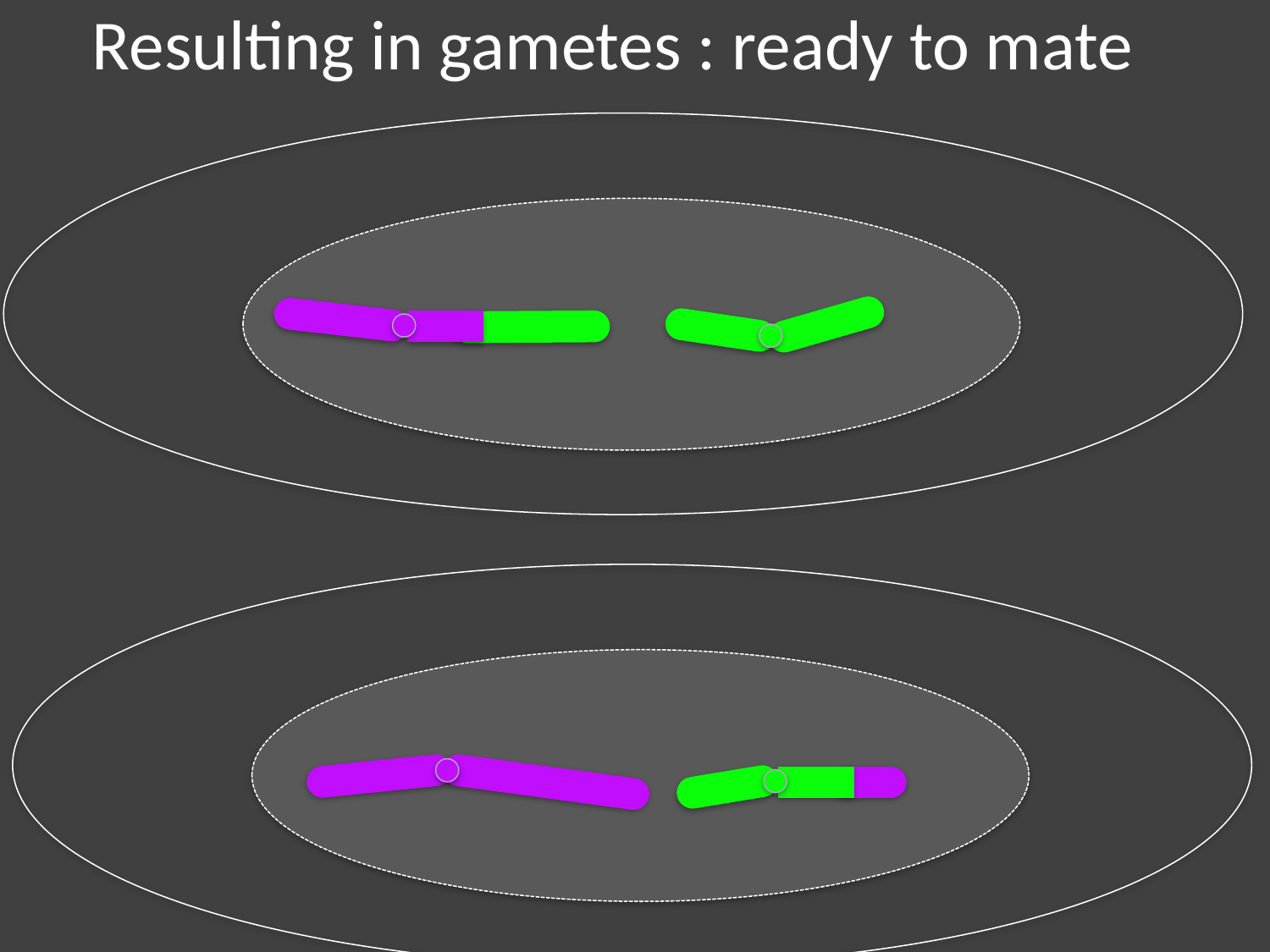

Resulting in gametes : ready to mate

## Slide 18
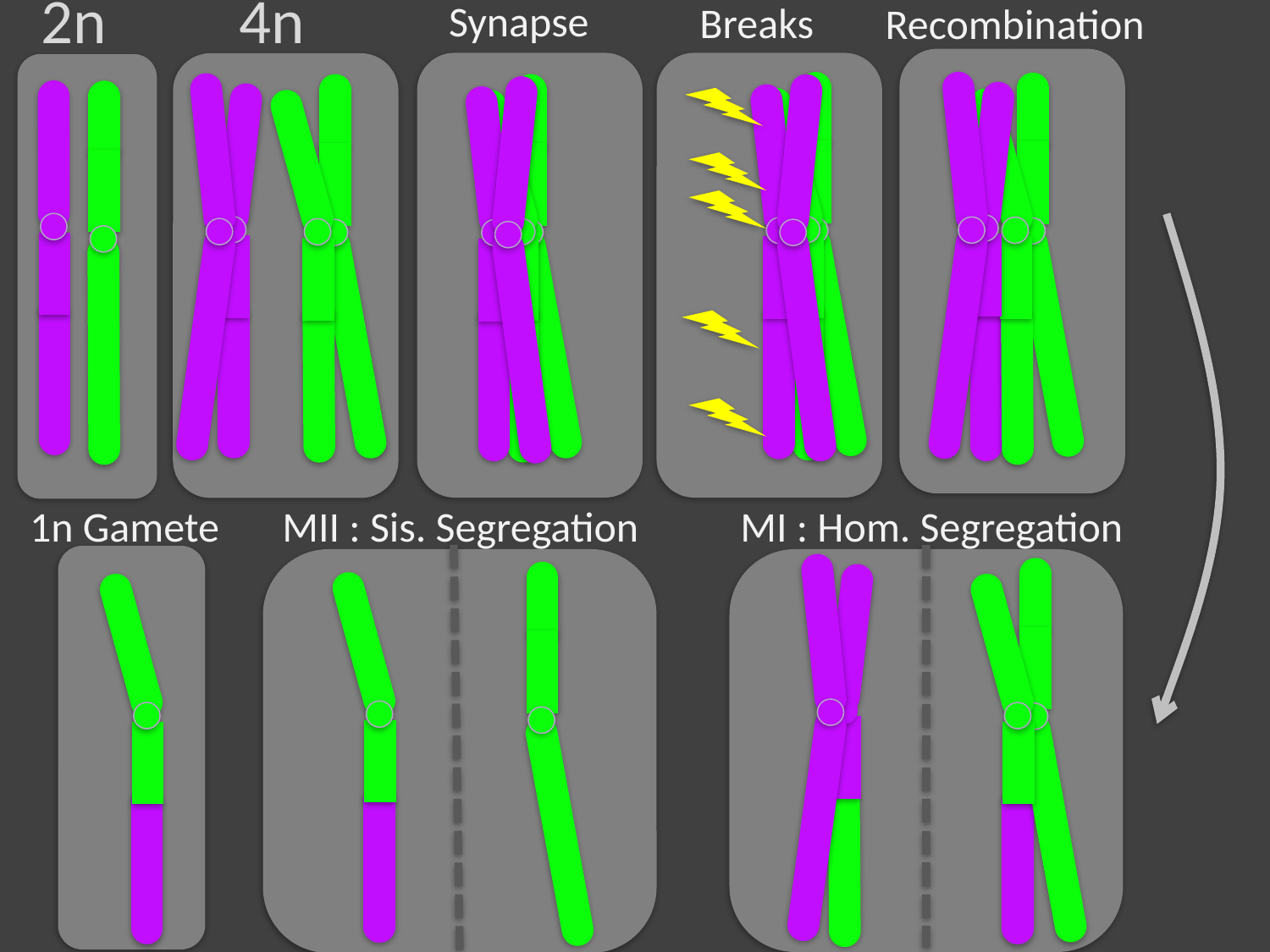

2n
4n
Synapse
Breaks
Recombination
1n Gamete
MII : Sis. Segregation
MI : Hom. Segregation

## Slide 19
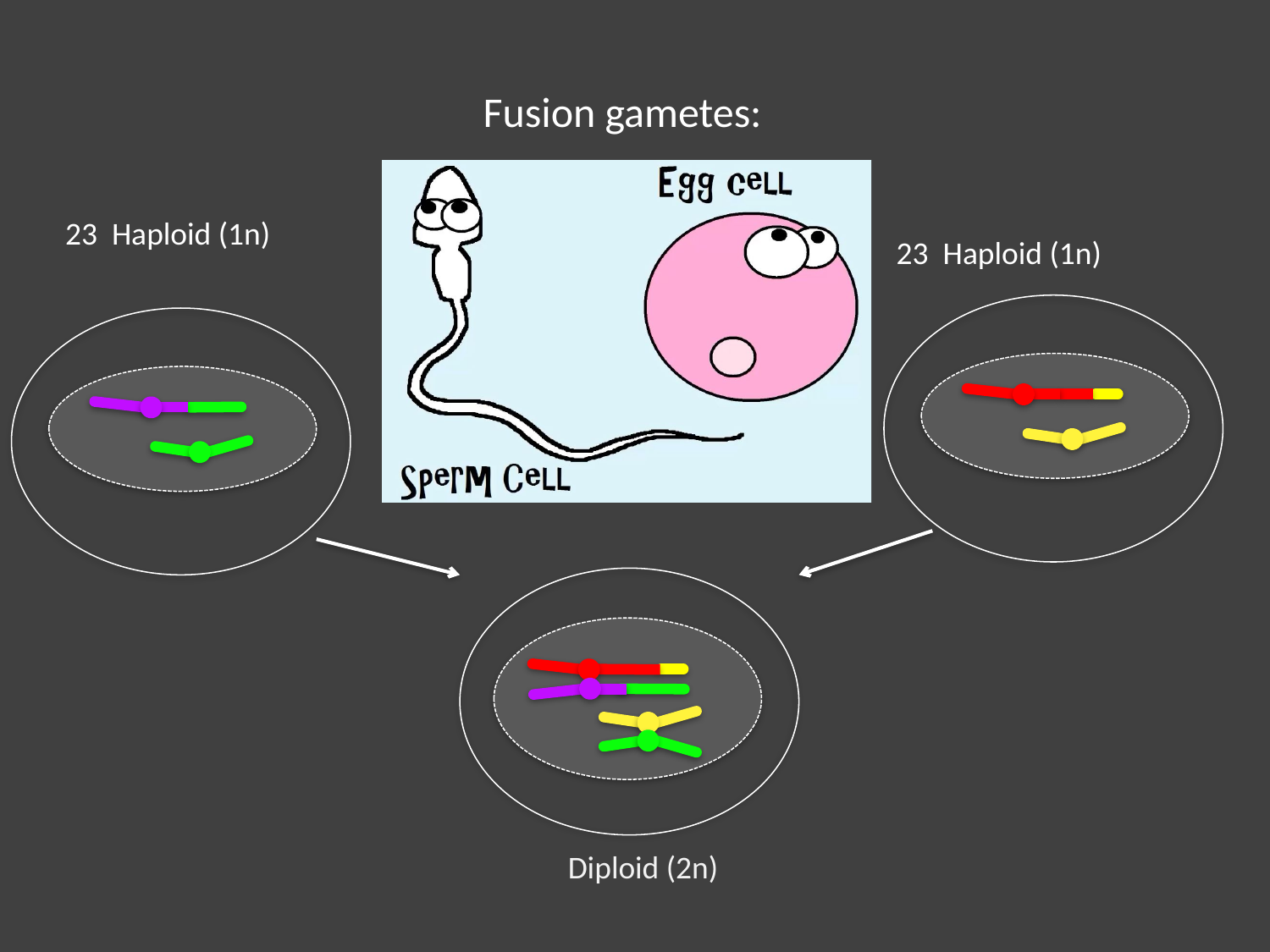

Fusion gametes:
23 Haploid (1n)
23 Haploid (1n)
Diploid (2n)

## Slide 20
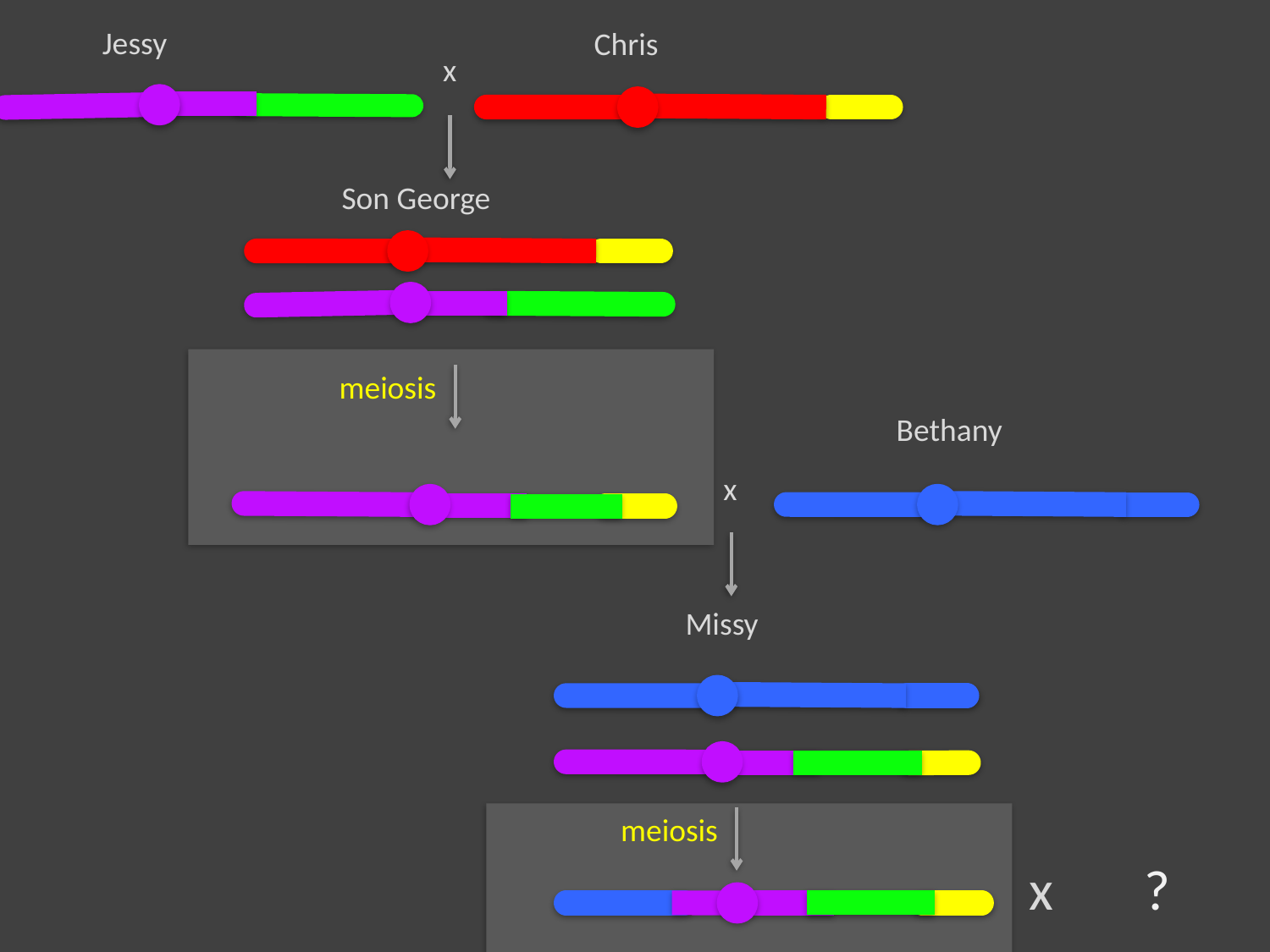

Jessy
Chris
x
Son George
meiosis
Bethany
x
Missy
meiosis
x
?

## Slide 21
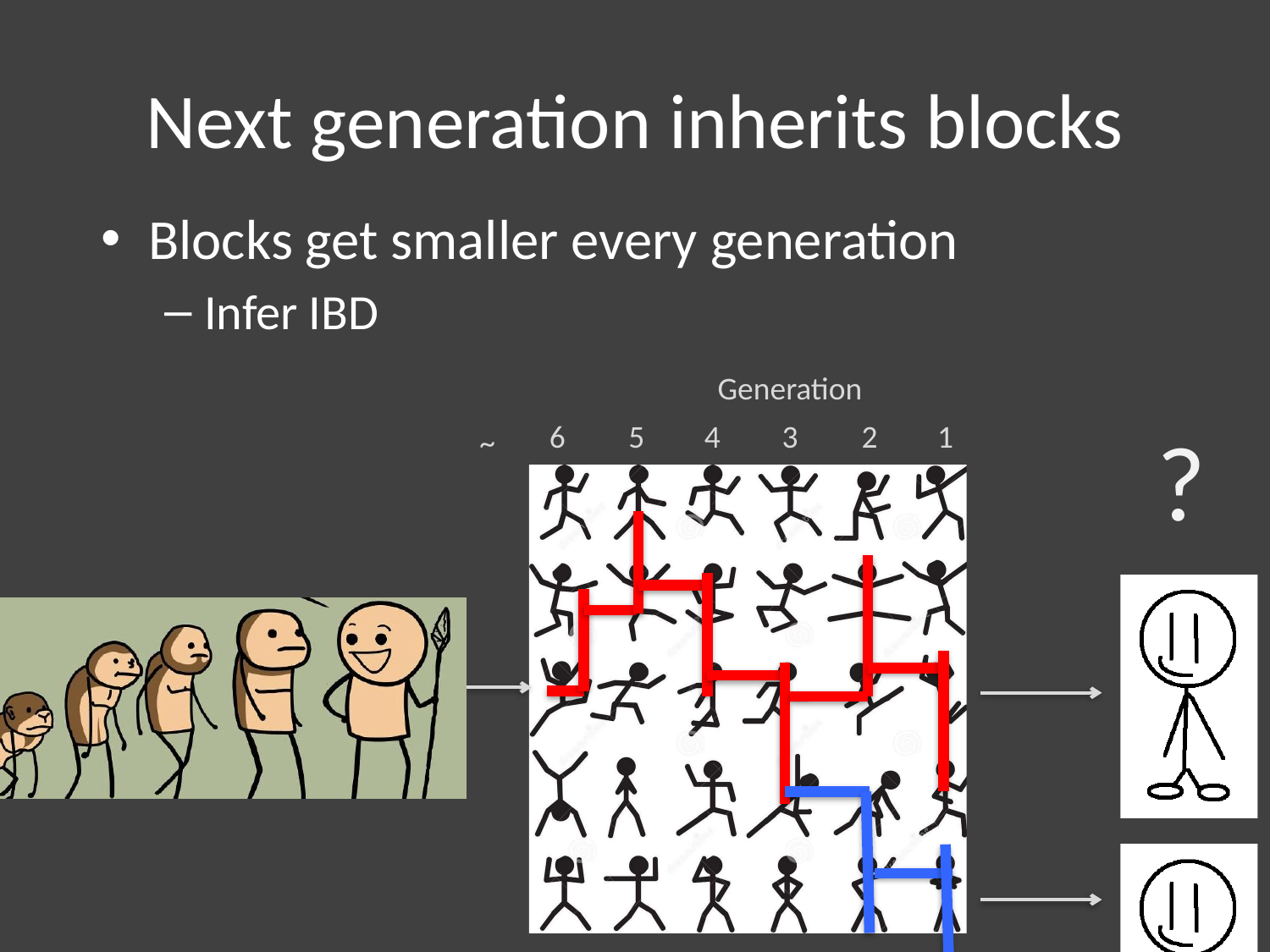

# Next generation inherits blocks
Blocks get smaller every generation
Infer IBD
Generation
?
6
5
4
3
2
1
~

## Slide 22
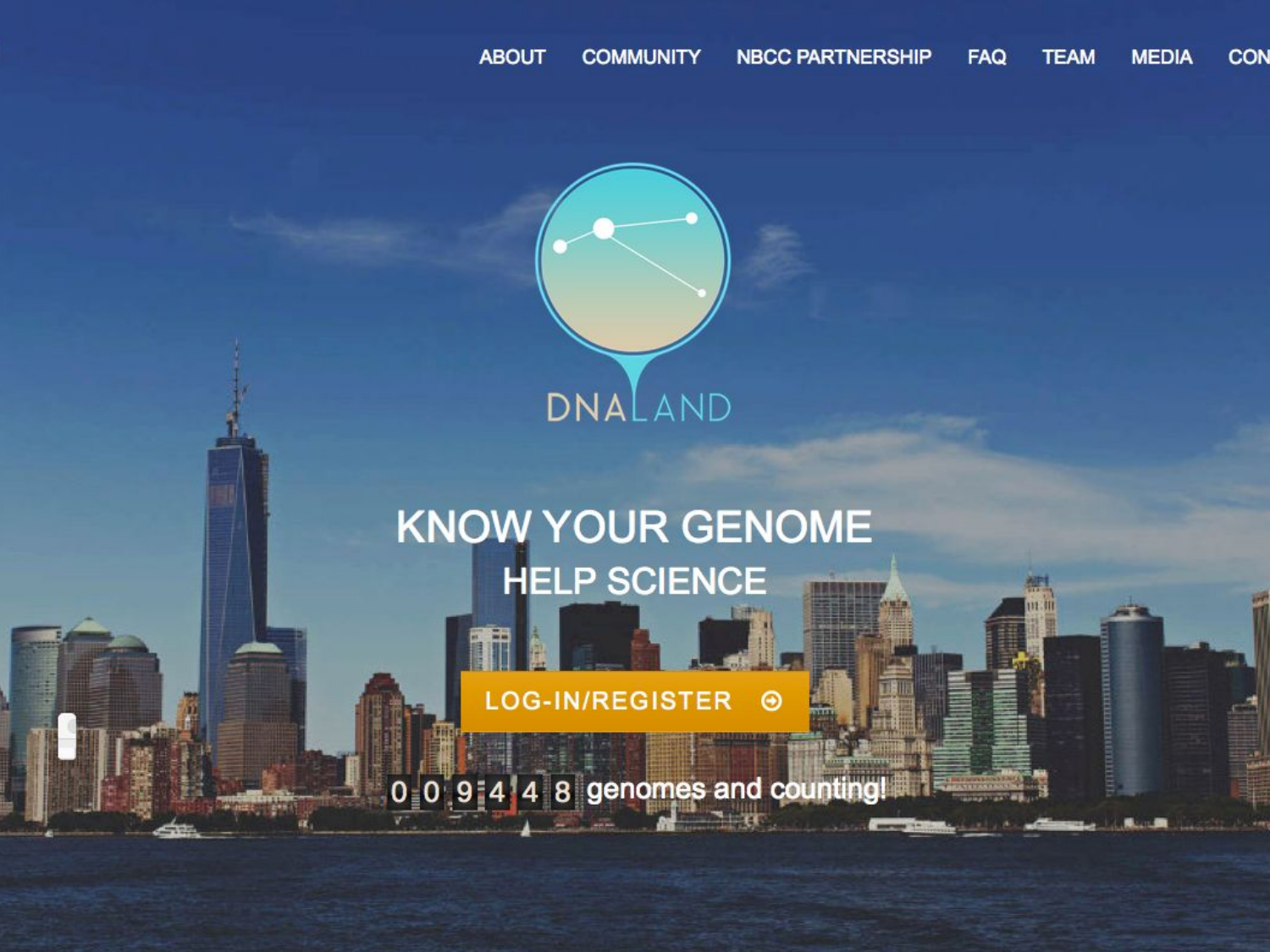

## Slide 23
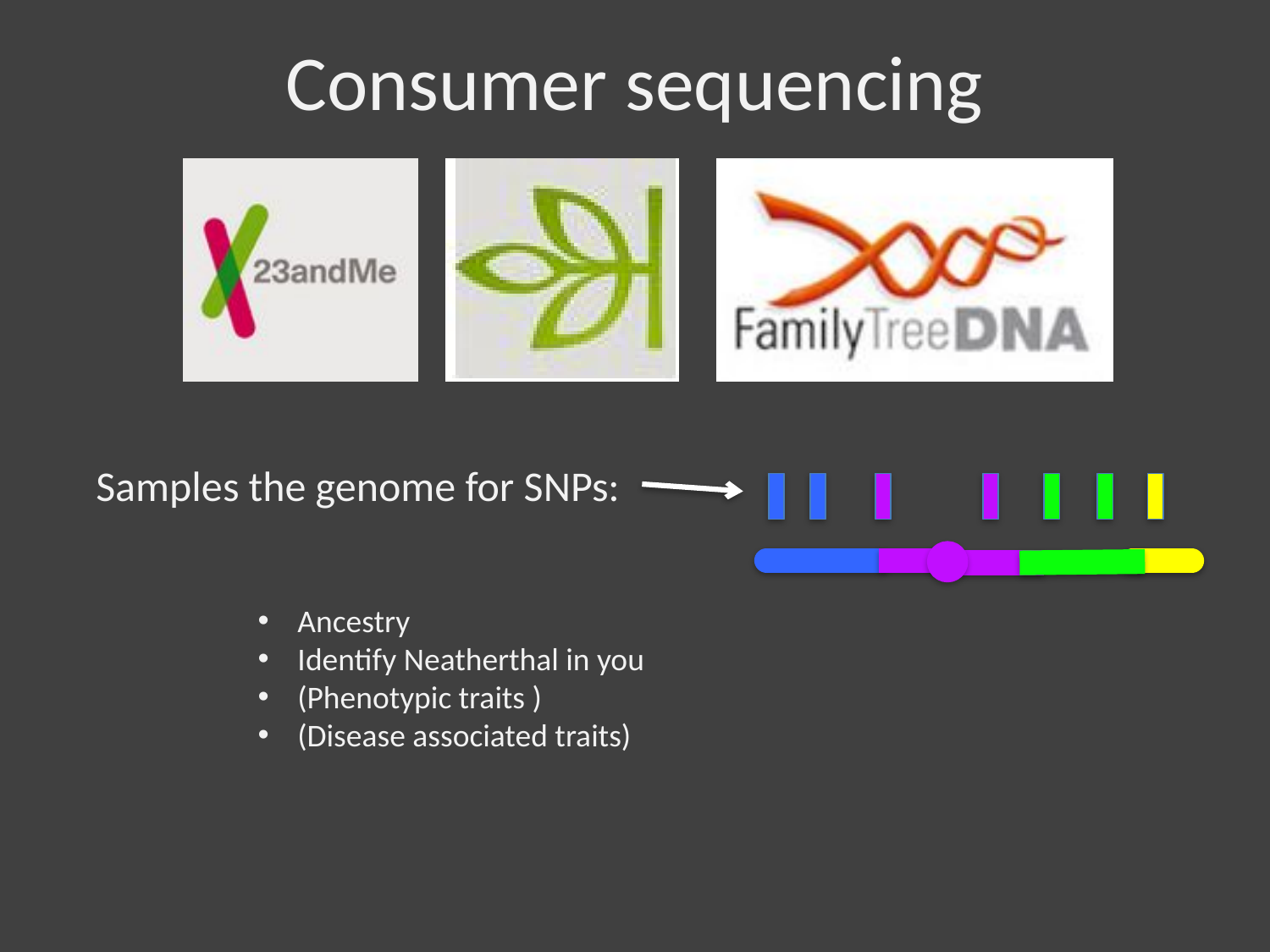

# Consumer sequencing
Samples the genome for SNPs:
Ancestry
Identify Neatherthal in you
(Phenotypic traits )
(Disease associated traits)

## Slide 24
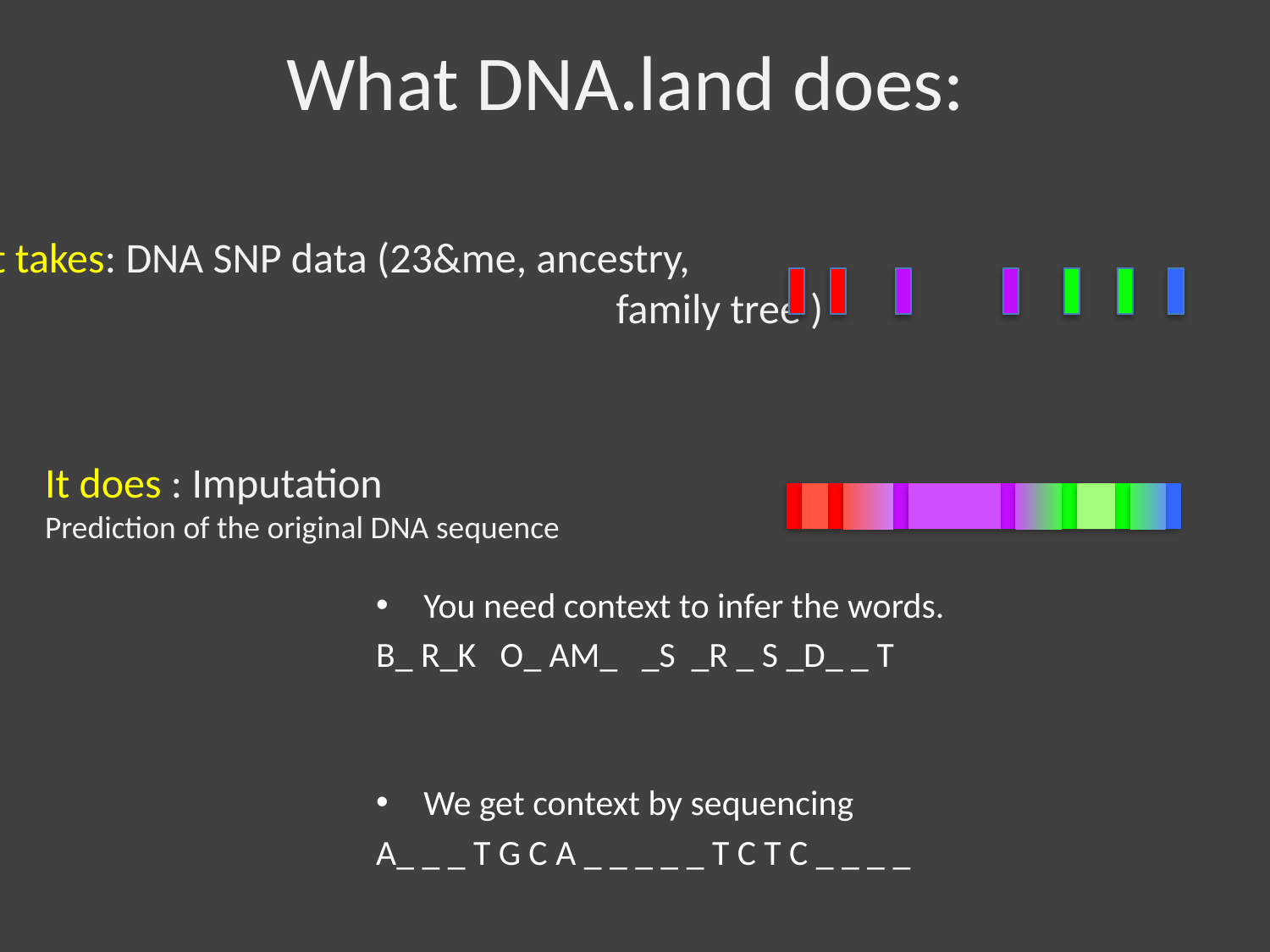

# What DNA.land does:
It takes: DNA SNP data (23&me, ancestry,
					family tree )
It does : Imputation
Prediction of the original DNA sequence
You need context to infer the words.
B_ R_K O_ AM_ _S _R _ S _D_ _ T
We get context by sequencing
A_ _ _ T G C A _ _ _ _ _ T C T C _ _ _ _

## Slide 25
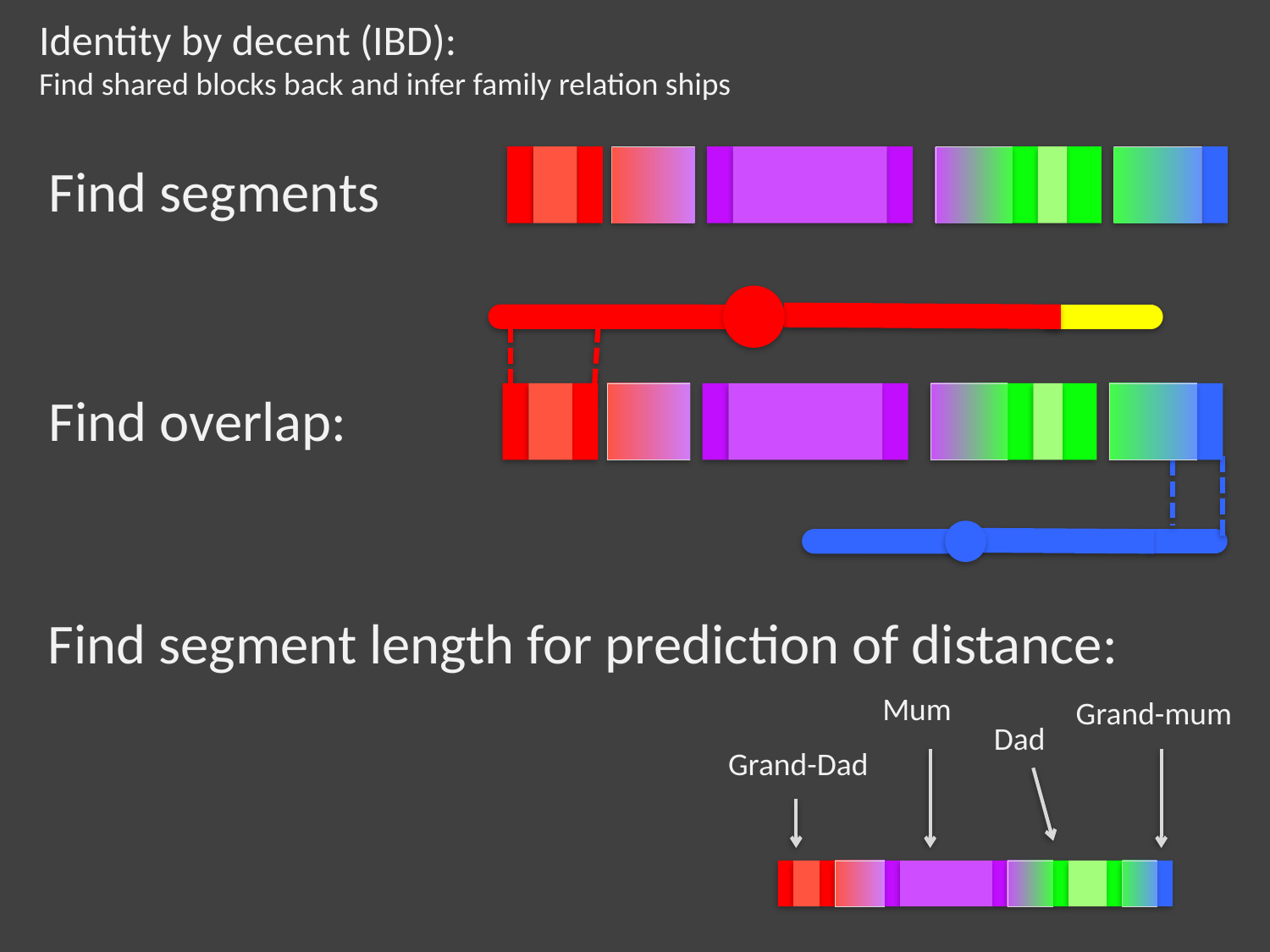

Identity by decent (IBD):
Find shared blocks back and infer family relation ships
Find segments
Find overlap:
Find segment length for prediction of distance:
Mum
Grand-mum
Dad
Grand-Dad

## Slide 26
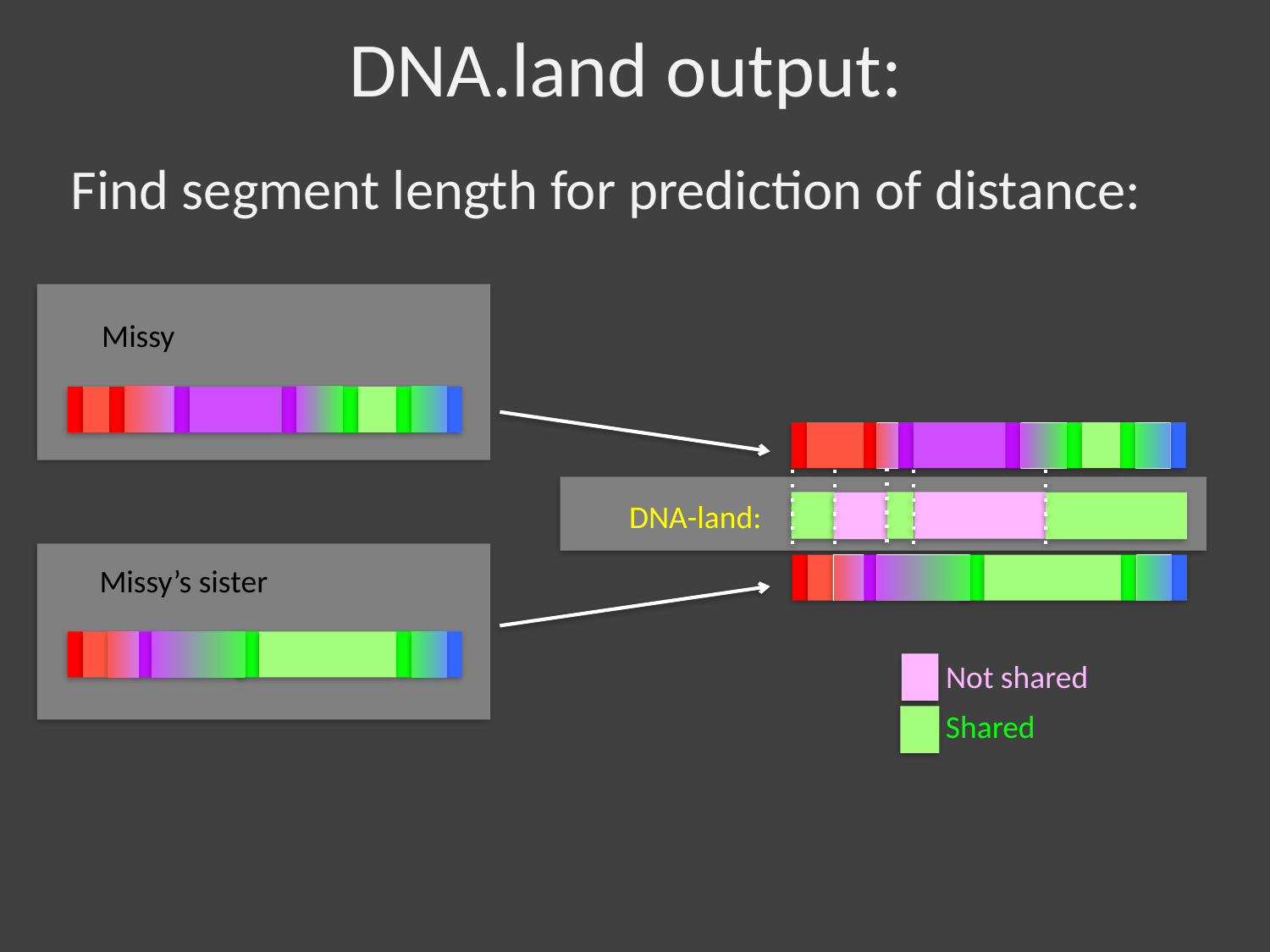

# DNA.land output:
Find segment length for prediction of distance:
Missy
DNA-land:
Missy’s sister
Not shared
Shared

## Slide 27
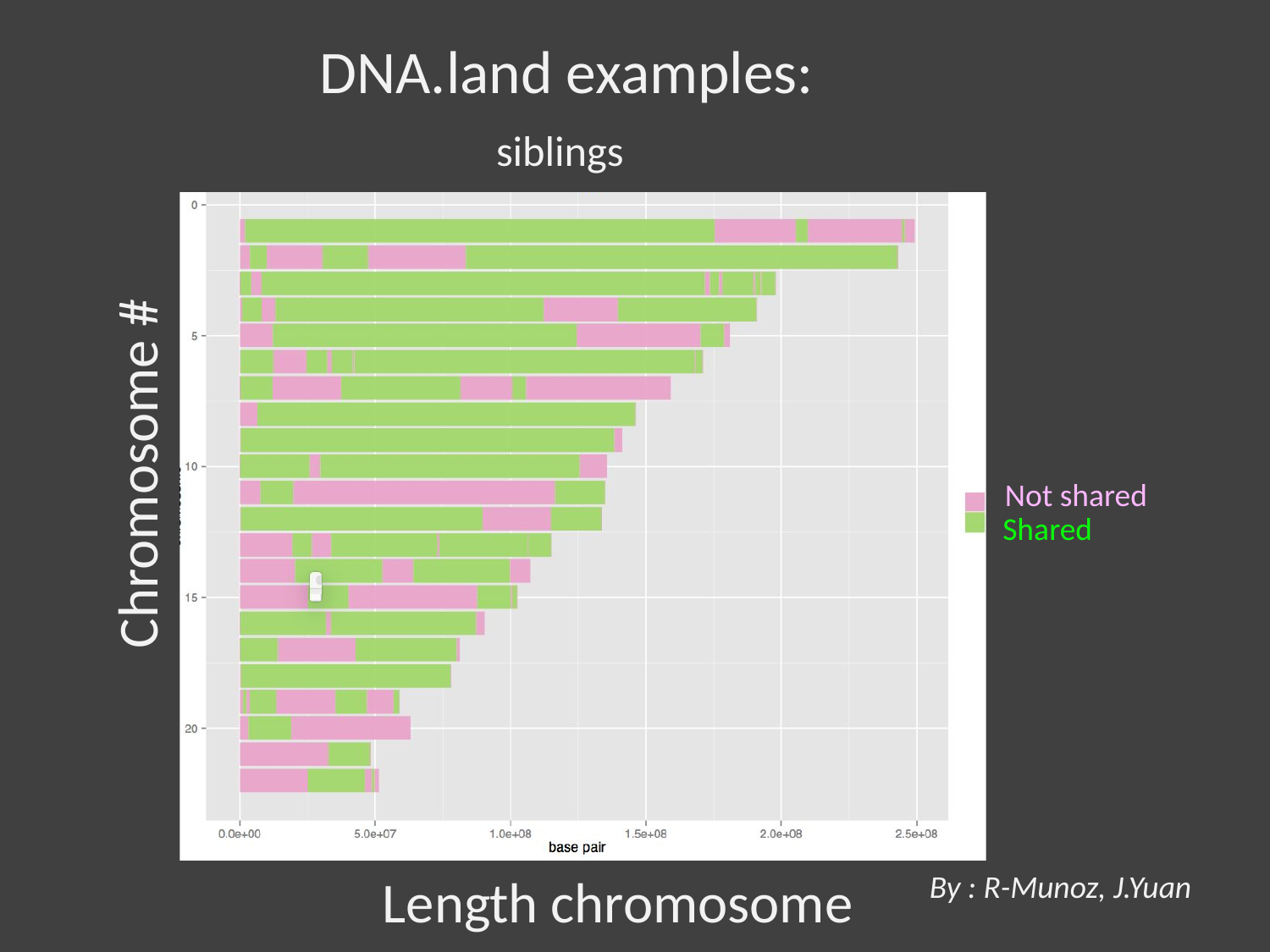

# DNA.land examples: siblings
Chromosome #
Not shared
Shared
Length chromosome
By : R-Munoz, J.Yuan

## Slide 28
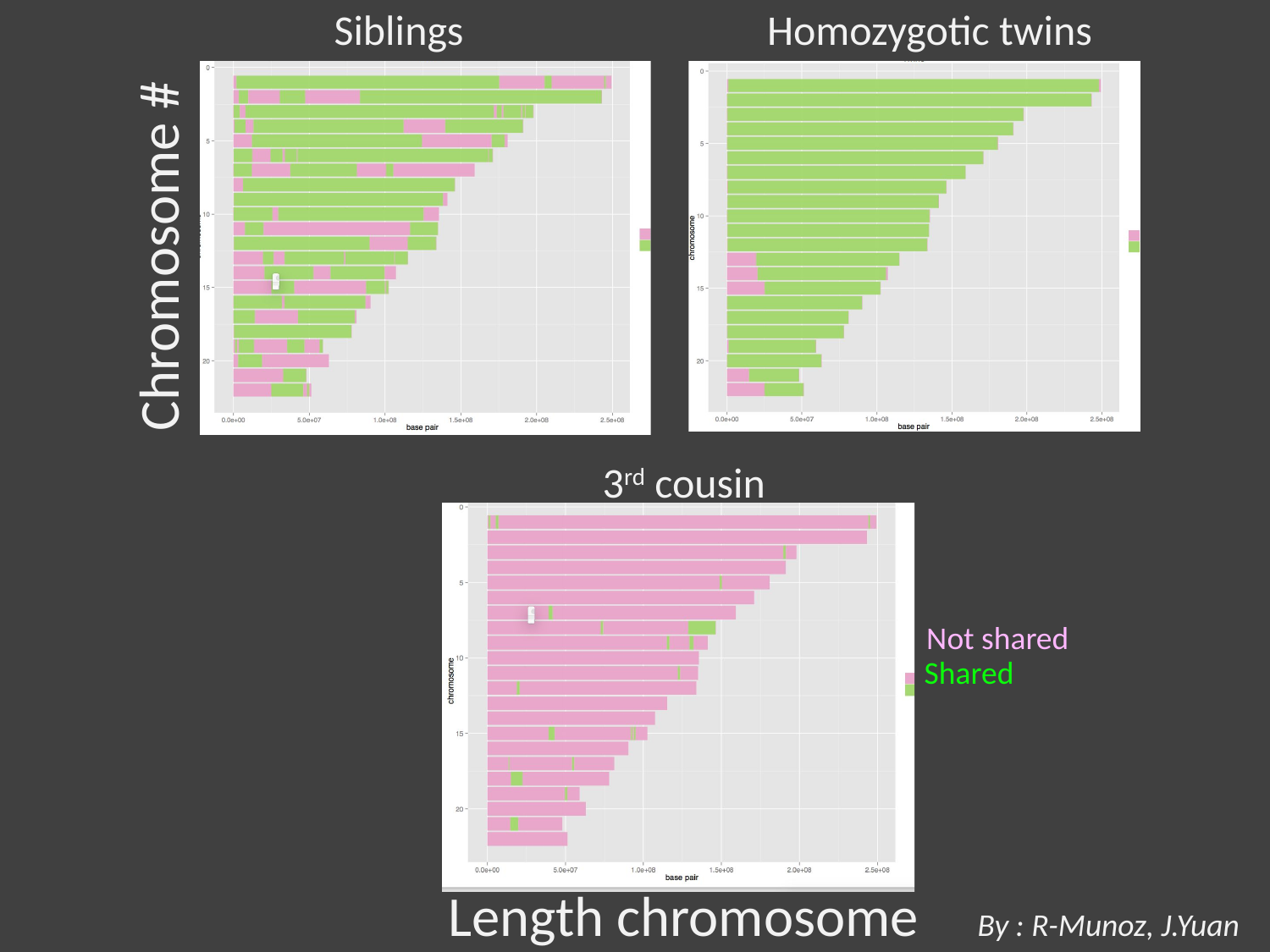

Siblings
Homozygotic twins
Chromosome #
3rd cousin
Not shared
Shared
Length chromosome
By : R-Munoz, J.Yuan

## Slide 29
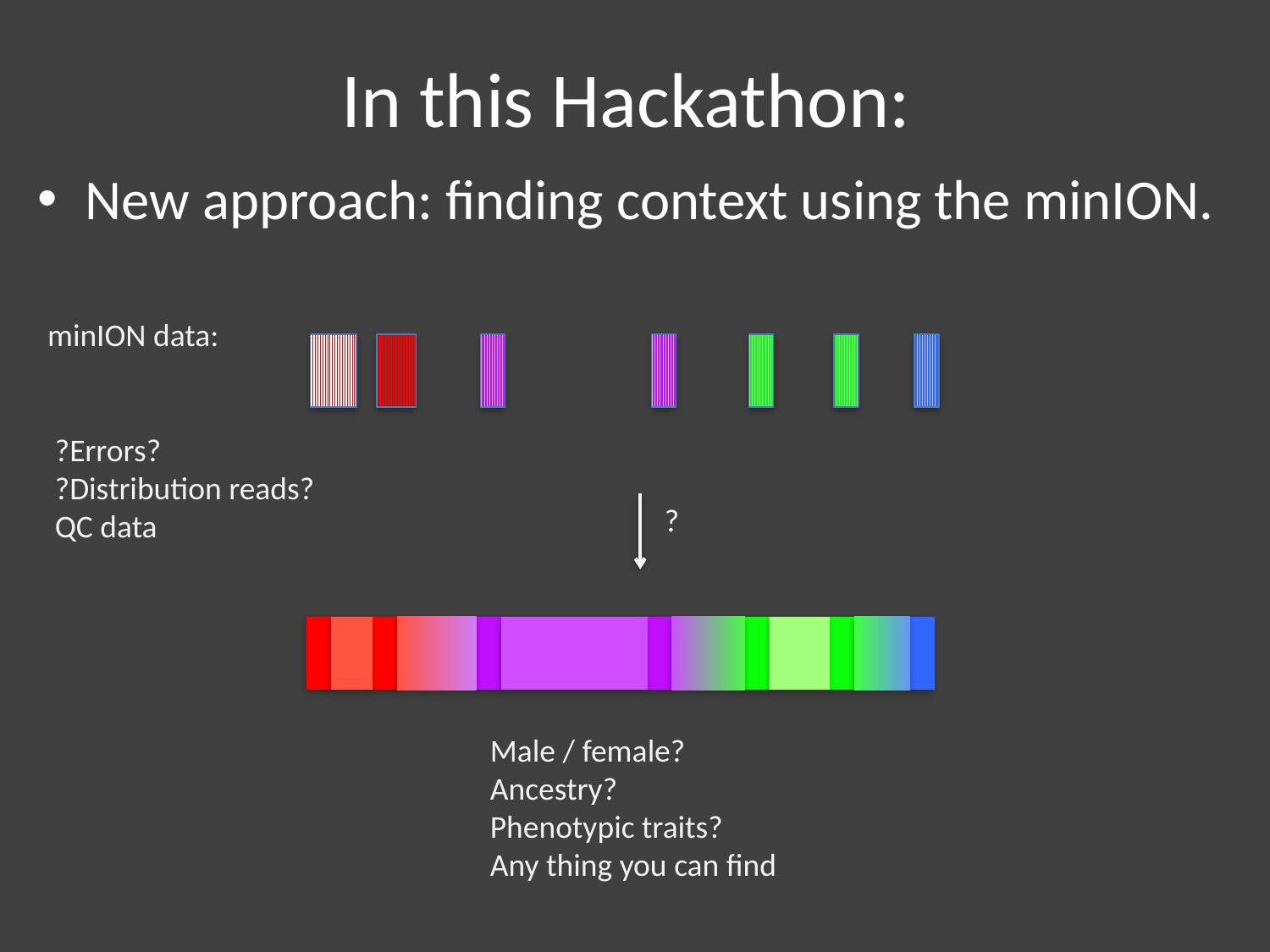

In this Hackathon:
New approach: finding context using the minION.
minION data:
?Errors?
?Distribution reads?
QC data
?
Male / female?
Ancestry?
Phenotypic traits?
Any thing you can find

## Slide 30
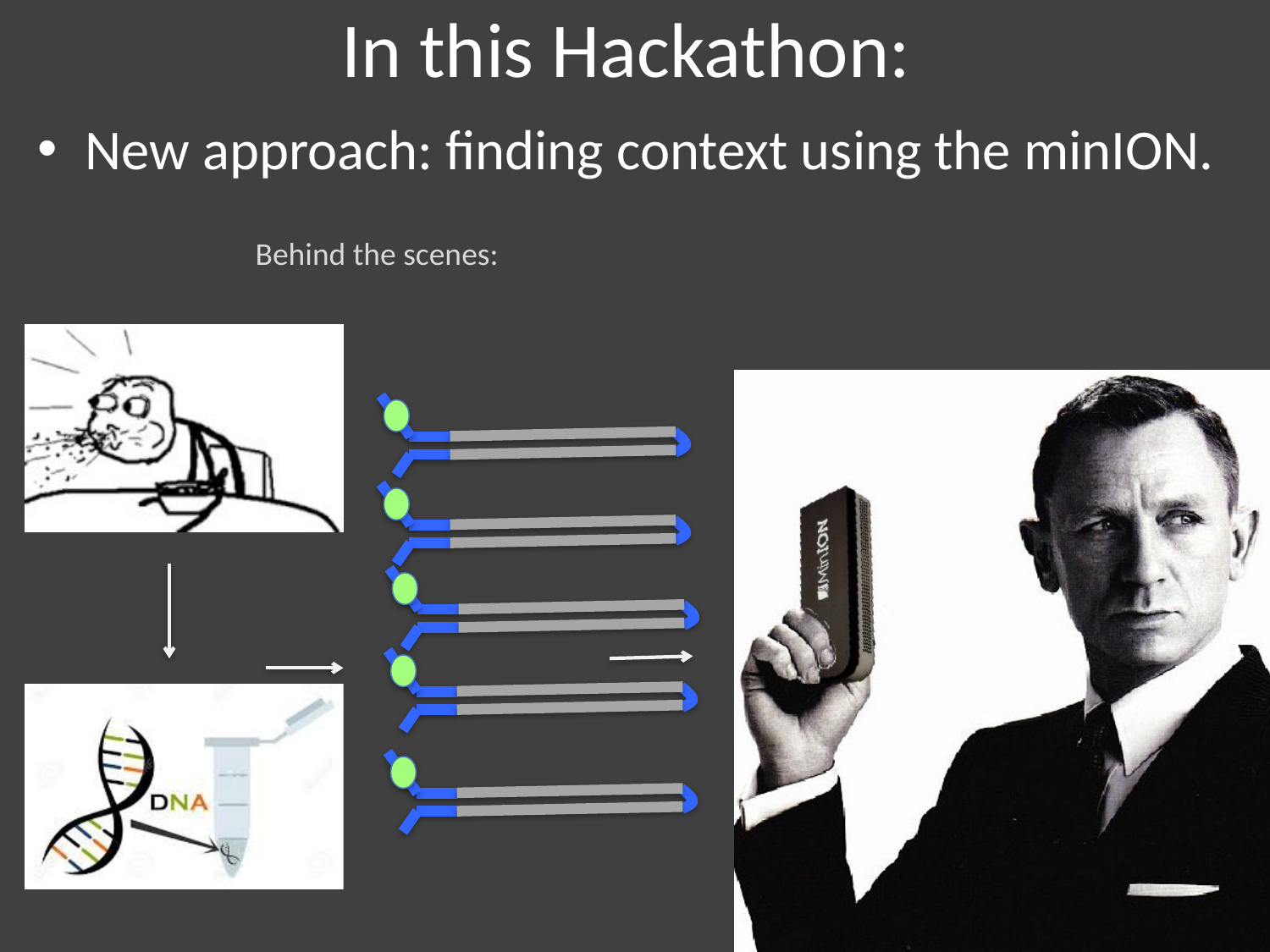

# In this Hackathon:
New approach: finding context using the minION.
Behind the scenes:

## Slide 31
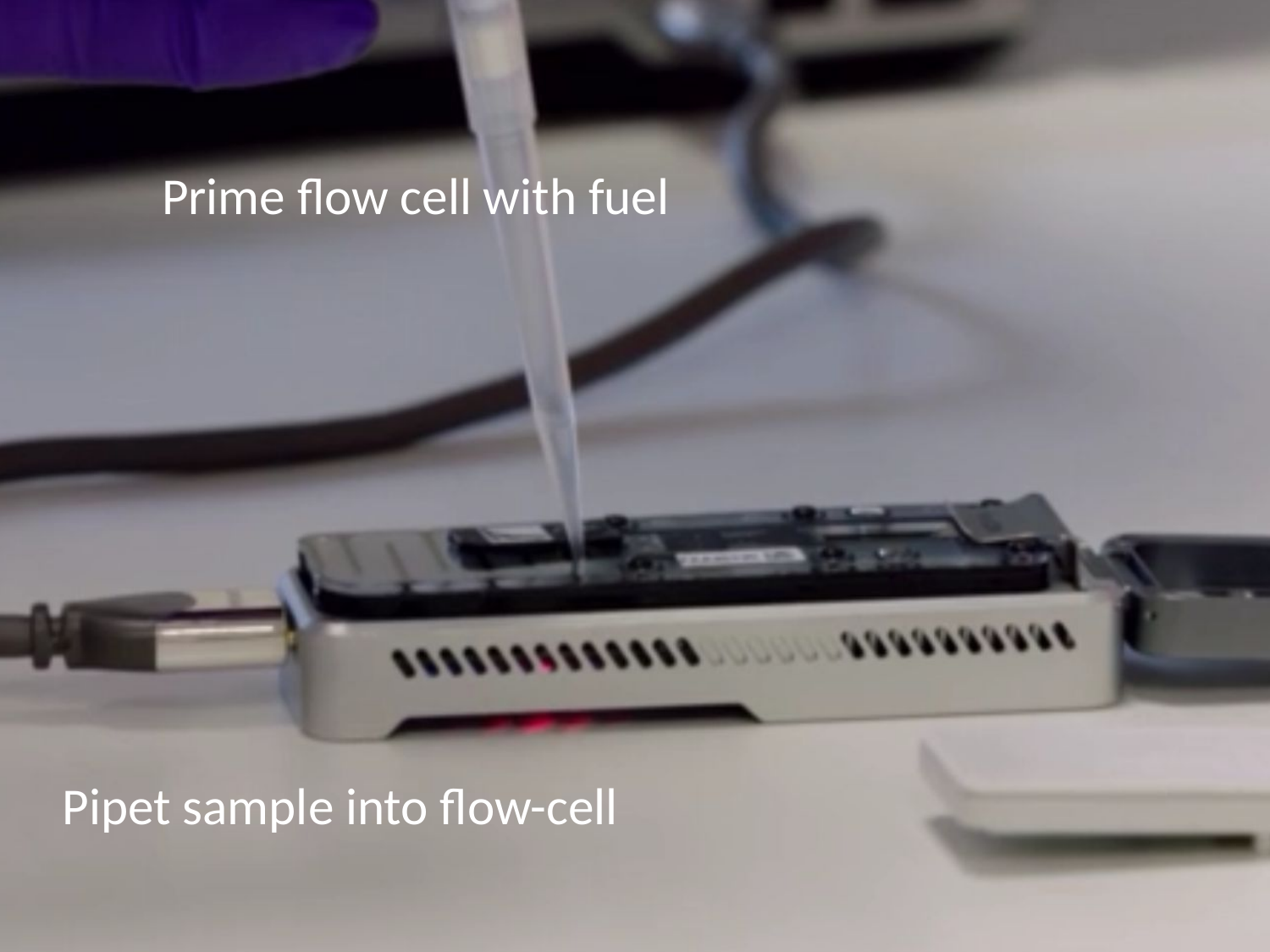

Prime flow cell with fuel
Pipet sample into flow-cell

## Slide 32
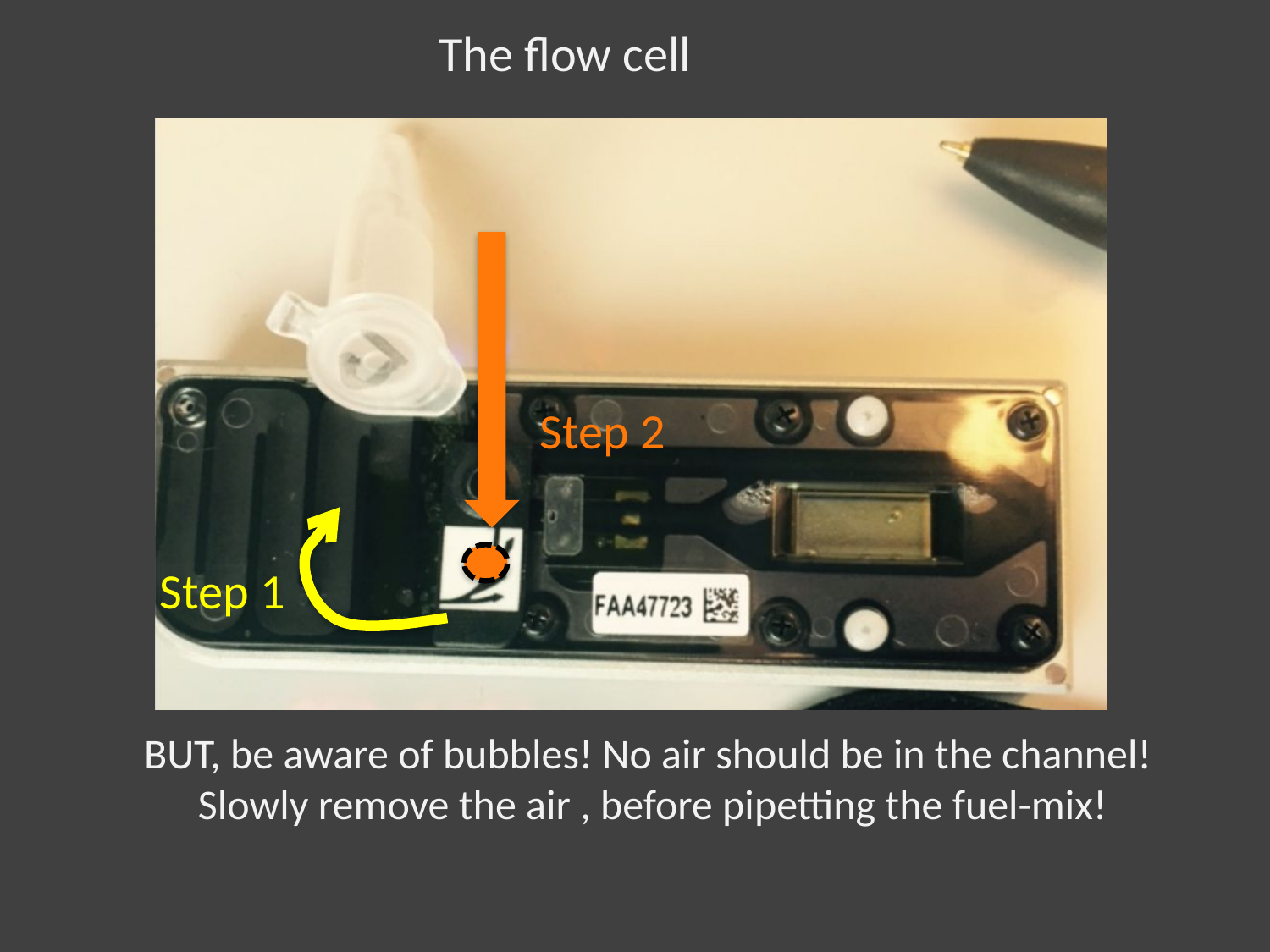

The flow cell
Step 2
Step 1
BUT, be aware of bubbles! No air should be in the channel!
Slowly remove the air , before pipetting the fuel-mix!

## Slide 33
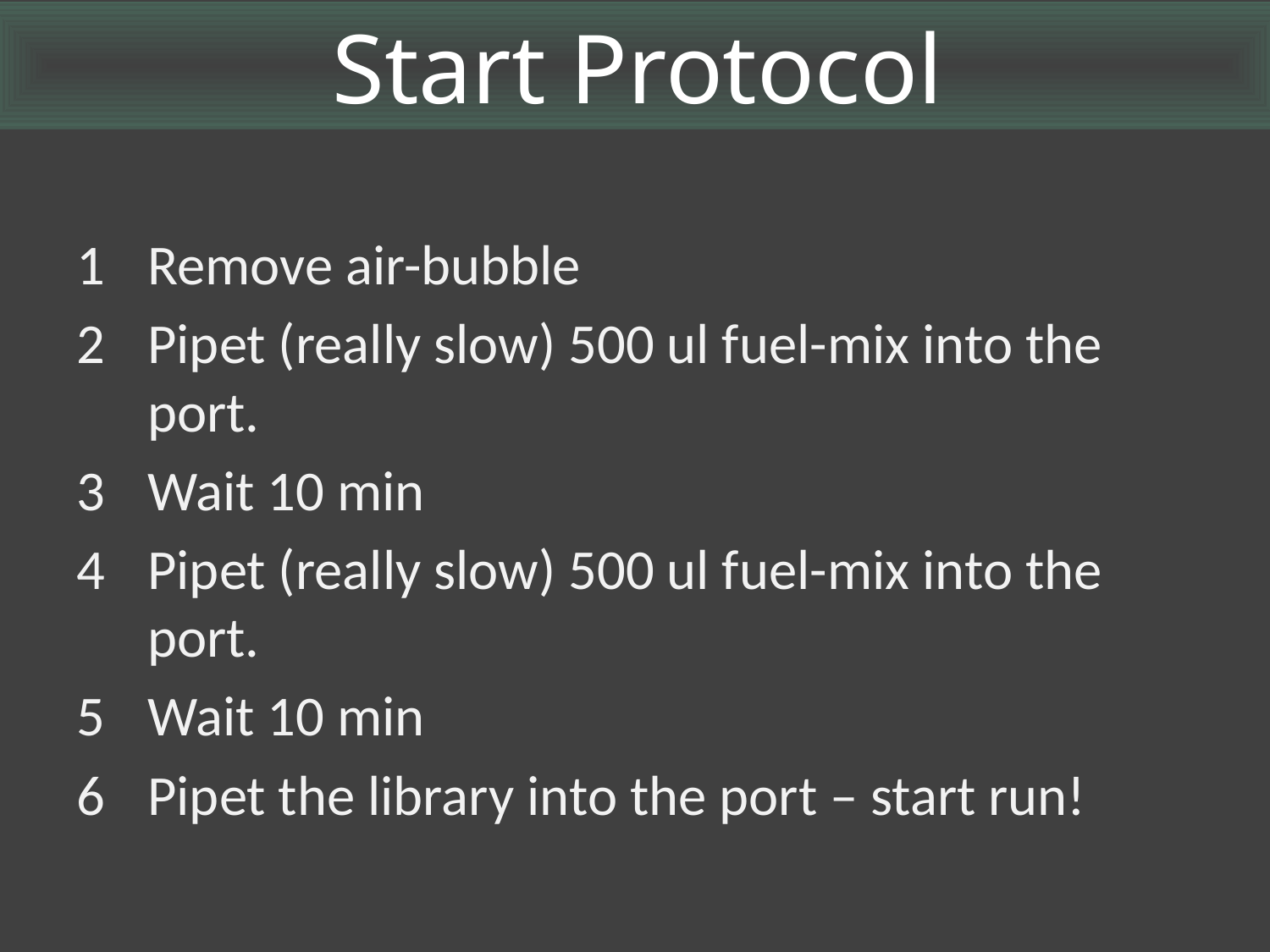

Start Protocol
Remove air-bubble
Pipet (really slow) 500 ul fuel-mix into the port.
Wait 10 min
Pipet (really slow) 500 ul fuel-mix into the port.
Wait 10 min
Pipet the library into the port – start run!

## Slide 34
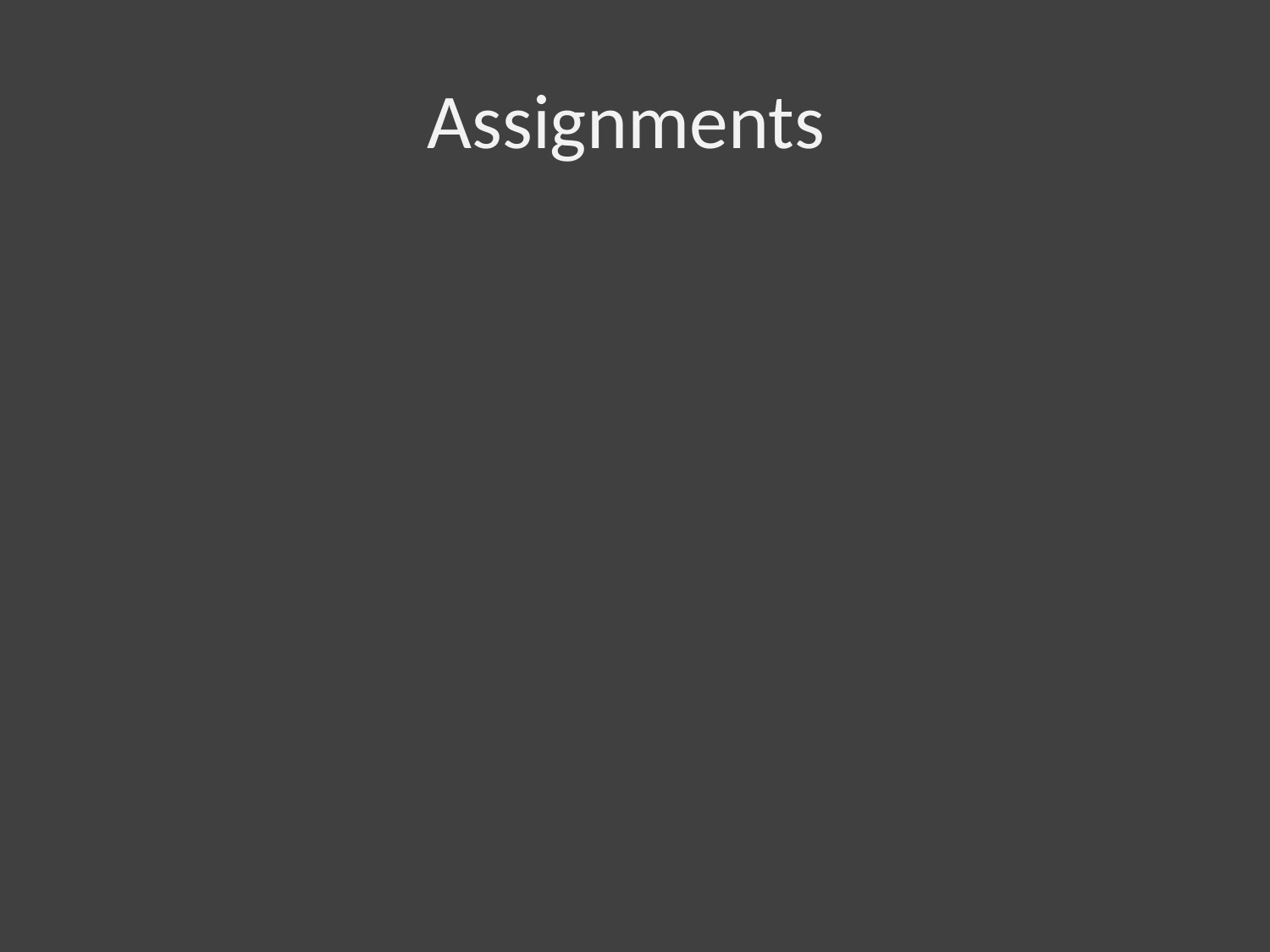

# Assignments
